# Early Antiretroviral Therapy Reduces AIDS Progression/Death in Individuals with Acute Opportunistic Infections: A Multicenter Randomized Strategy Trial

**DOI:** 10.1371/journal.pone.0005575

**Published:** 2009-05-18

**Authors:** Andrew R. Zolopa, Janet Andersen, Lauren Komarow, Ian Sanne, Alejandro Sanchez, Evelyn Hogg, Carol Suckow, William Powderly, for the ACTG A5164 study team

**Affiliations:** 1 Stanford University AIDS Clinical Trials Unit, Stanford University, Stanford, California, United States of America; 2 Statistical and Data Analysis Center, Harvard School of Public Health, Boston, Massachusetts, United States of America; 3 University College Dublin, Belfield, Ireland; 4 University of Southern California, Los Angeles, California, United States of America; 5 Wits Health Consortium, Helen Joseph Hospital, Johannesburg, South Africa; 6 Frontier Science & Technology Research Foundation, Amherst, New York, United States of America; 7 Social & Scientific Systems, Inc., Silver Spring, Maryland, United States of America; 8 Statistical and Data Analysis Center, Harvard School of Public Health, Boston, Massachusetts, United States of America; St. Vincent's Hospital, Australia

## Abstract

**Background:**

Optimal timing of ART initiation for individuals presenting with AIDS-related OIs has not been defined.

**Methods and Findings:**

A5164 was a randomized strategy trial of “early ART” - given within 14 days of starting acute OI treatment versus “deferred ART” - given after acute OI treatment is completed. Randomization was stratified by presenting OI and entry CD4 count. The primary week 48 endpoint was 3-level ordered categorical variable: 1. Death/AIDS progression; 2. No progression with incomplete viral suppression (ie HIV viral load (VL) ≥50 copies/ml); 3. No progression with optimal viral suppression (ie HIV VL <50 copies/ml). Secondary endpoints included: AIDS progression/death; plasma HIV RNA and CD4 responses and safety parameters including IRIS.

282 subjects were evaluable; 141 per arm. Entry OIs included *Pneumocytis jirovecii* pneumonia 63%, cryptococcal meningitis 12%, and bacterial infections 12%. The early and deferred arms started ART a median of 12 and 45 days after start of OI treatment, respectively.

The difference in the primary endpoint did not reach statistical significance: AIDS progression/death was seen in 20 (14%) vs. 34 (24%); whereas no progression but with incomplete viral suppression was seen in 54 (38%) vs. 44 (31%); and no progression with optimal viral suppression in 67 (48%) vs 63 (45%) in the early vs. deferred arm, respectively (p = 0.22). However, the early ART arm had fewer AIDS progression/deaths (OR = 0.51; 95% CI = 0.27–0.94) and a longer time to AIDS progression/death (stratified HR = 0.53; 95% CI = 0.30–0.92). The early ART had shorter time to achieving a CD4 count above 50 cells/mL (p<0.001) and no increase in adverse events.

**Conclusions:**

Early ART resulted in less AIDS progression/death with no increase in adverse events or loss of virologic response compared to deferred ART. These results support the early initiation of ART in patients presenting with acute AIDS-related OIs, absent major contraindications.

**Trial Registration:**

ClinicalTrials.gov NCT00055120

## Introduction

Over the past decade there has been remarkable progress in the treatment of HIV-1 infection. As a result of potent combination antiretroviral therapy (ART), HIV-infected individuals are living longer and healthier lives. [1]–[3] Even patients who initiate treatment relatively late in the disease course have been shown to benefit from ART treatment. [4]–[6]. Despite these advances, mortality rates remain unacceptably high in populations with poor access to health care services such as communities of color, young adults, and poor rural and inner-city dwellers [7]–[12] who frequently first enter HIV care with acute AIDS-related opportunistic infections (OIs). In addition, many thousands of HIV-infected individuals are accessing ART in Resource-poor settings, often with advanced AIDS.

As we enter the second decade of effective ART, an important clinical question has remained unanswered: when should ART be started in the management of a patient with an acute OI? Concurrent treatment of the OI and HIV might result in higher morbidity and/or mortality by increasing toxicity of treatment, increasing drug-drug interactions, decreasing adherence to the OI regimen, and increasing the frequency of immune reconstitution and inflammatory syndrome (IRIS) reactions. Alternatively, concurrent treatment might decrease patient morbidity/mortality by restoring pathogen-specific immune responses and speeding immune reconstitution.

In this randomized trial, we address the optimal timing of ART in the setting of an acute OI by evaluating two clinical approaches or strategies; “early ART” intended to be initiated during OI treatment, and “deferred ART” intended to be initiated after treatment of the acute OI is completed.

## Methods

The protocol for this trial and supporting CONSORT checklist are available as supporting information; see Checklist S1 and Protocol S1.

### Trial Design

AIDS Clinical Trials Group (ACTG) A5164 was an open-label, randomized, phase IV, strategy study of early-versus-deferred ART in subjects who presented with acute AIDS-related OIs or serious bacterial infections (BIs) for which effective antimicrobial therapies were available. The OIs were a subset of the 1999 CDC's AIDS-defining conditions or treatable AIDS-related OIs [13]. (See Appendix S1.)

After eligibility checklist (including safety laboratories) was completed, randomized treatment assignment was generated by central computer using permuted blocks within strata. Neither the size of the blocks nor treatment assignments to other sites were public, preventing individual investigators from deducing the assignment pattern. Randomization was stratified by CD4 cell count (< or ≥50 cells/mm^3^) and by first treated OI/BI at study entry (PCP, vs. BIs, vs. all other OIs).

Subjects had to be randomized within 14 days of starting therapy for the OI/BI that determined study eligibility. Subjects in the early arm (also referred to as “immediate arm” in study protocol) were expected to start ART within 48 hours of study enrollment. Subjects in the deferred arm were encouraged to start ART between week 6 (day 42) and week 12 (day 84) of the study; to receive study-provided ART, they were required to start treatment between week 4 (day 28) and week 32 (day 224). Subjects in the deferred arm who started ART outside this window were not offered study-provided ART but were included in analyses. All subjects in both arms were followed for 48 weeks, regardless of whether they started or continued ART and were included in the analyses.

The primary endpoint of the study was a 3-level, ordered, categorical variable: alive without AIDS progression and with HIV viral load <50 copies/mL (best outcome) at week 48; alive without AIDS progression and with detectable HIV viral load (i.e. VL > = 50 copies/ml) at week 48 (intermediate outcome); and AIDS progression or death (worst outcome) at any time. New AIDS-defining events diagnosed in the first 30 days of the study were not considered to be endpoints because it was highly likely they existed at entry but were not diagnosed until a more thorough work-up after the acute event was under control. Sensitivity analyses were conducted with a 15 day window to confirm results. Endpoints were also evaluated without the 30-day window. Several of the planned secondary outcomes reported here include clinical outcomes: death/AIDS progression independent of virologic response; virologic response independent of clinical endpoints; changes in CD4 counts from baseline over 48 weeks; safety and tolerability of ART; hospitalizations; self-reported ART adherence; and incidence of IRIS. Other secondary endpoints on incidence of drug resistance, quality of life and functional status as well as a formal cost-effectiveness analysis will be the focus of future manuscripts.

### Subjects

Eligible subjects were HIV-infected men or women >13 years old, presenting with an AIDS-defining OI or serious BI for which effective antimicrobial therapy was available and prescribed. To reflect clinical practice, the trial allowed presumptive and confirmed diagnoses as long as appropriate treatment for the OI/BI had been initiated (cryptococcal disease was required to be confirmed). Subjects with BIs must have had a CD4 count confirmed of <200 cells/mm^3^. Subjects with or on treatment for tuberculosis (TB) were excluded. Subjects in whom TB was diagnosed after randomization remained in the study. Subjects were ineligible if they had received ART within 8 weeks prior to study entry, more than 31 days of any ART within 6 months prior to study entry, or more than one ART regimen on which they experienced treatment failure. Recruitment was conducted at 39 AIDS Clinical Trials Units in the United States (including Puerto Rico), and Johannesburg, South Africa (which was limited to enrolling 20 subjects by the study sponsor). (see Appendix S2 for complete investigator listing)

#### Ethics statement

All participating sites had local Institutional Review Board approval. All subjects provided written informed consent.

### Treatment Regimens

The study provided lopinavir/ritonavir [LPV/r], emtricitabine [FTC], tenofovir disoproxil fumarate [TDF], and stavudine [d4T]. However, any antiretroviral agent approved by the FDA for the initial treatment of HIV was allowed. The study recommended that ART include a ritonavir-boosted protease inhibitor (PI/r) or non-nucleoside reverse transcriptase inhibitor (NNRTI) in combination with 2 nucleoside reverse transcriptase inhibitors (nRTIs) that included lamivudine [3TC] or emtricitabine [FTC], but the choice of ART was left to the judgment of the clinician to better reflect common clinical practice.

### Study Schedule

Subjects were seen at weeks 4, 8, 12, 16, and every 8 weeks thereafter through week 48 for clinical assessments and routine laboratory monitoring. Subjects in the deferred arm shifted to follow-up at weeks 4, 8, 12, and 16 after initiation of ART and every 8 weeks thereafter until week 48. Adverse events were graded from Grade 2 (moderate) to Grade 4 (worst) according to the Division of AIDS Table for Grading the Severity of Adult and Pediatric Adverse Events: December 2004. Grade 2 events were only reported if they lead to a modification or discontinuation of ART. They are presented here for completeness, as ART management is an important consideration of this study. Clinical assessments and laboratories were also measured at time of suspected IRIS and premature ART or study discontinuation. Adherence documenting missed doses over the prior four days and within 3 months was assessed by self-report at weeks 8, 16, 32 and 48. The National Institute of Allergy and Infectious Diseases' Data and Safety Monitoring Board (DSMB) reviewed the study annually.

### IRIS Case Definition

The definition of IRIS was: evidence of an increase in CD4+ cell counts and/or decrease in plasma viral load in response to ART; and symptoms consistent with an infectious/inflammatory condition, temporally related to the initiation of ART, that could not be explained by a newly acquired infection, the expected clinical course of another agent, or side effects of ART itself. All cases of IRIS required review by a study chair, blinded to treatment group and site. One case that could not be blinded was reviewed by a third reviewer who was not part of the study team.

### Statistical Considerations

Analyses of the primary ordered endpoint and related secondary endpoints were conducted with exact Wilcoxon tests stratified by entry CD4 count and OI/BI and logistic regression adjusted for entry CD4 count and OI/BI. Analyses of related censored data were conducted with stratified Cox regression. Unstratified tests were conducted as appropriate, including: dichotomous covariates with Fisher's exact tests; ordered and continuous data with Wilcoxon and Kruskall-Wallis tests; logistic regression and censored data with unstratified logrank tests. Baseline in all analyses was defined as the date of randomization. Loss to follow-up and, where not the endpoint, deaths were censored in failure-time analyses. Cumulative incidence models with death as a competing risk were evaluated. Curves depicting censored data are based on the method of Kaplan and Meier and do not reflect stratification.

The target sample size was 141 per arm to provide at least 80% power for a range of plausible alternatives for the distribution of the primary endpoint, based on improving the proportion in the best outcome by approximately 20% (e.g. 40% to 60%) and a reduction by 1/3 in the worst outcome (e.g., 15% to 10%). The expected lost to follow up rate was 10%–15%. The DSMB reviewed one formal interim analysis and thus the primary and related secondary endpoints should be evaluated with respect to a two-sided 0.0448 p-value.

## Results

**Figure 1:**
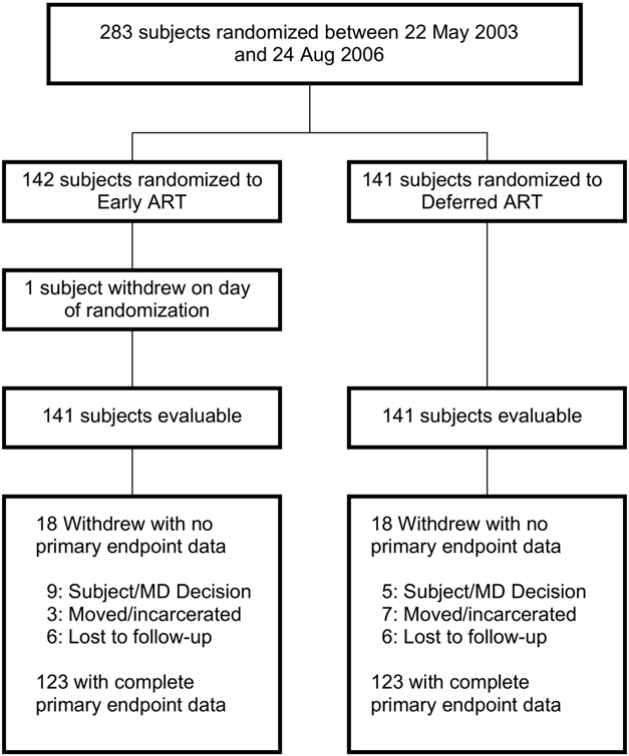
Study Subject Enrollment and Discontinuation.

A5164 enrolled 283 subjects between May 2003 and August 2006. The last subject completed the study in August of 2007 and data entry was completed by January 2008. One subject withdrew consent on the day of randomization and is not included in the analysis. (Figure 1) Baseline demographic and clinical characteristics for the 282 evaluable subjects were well balanced across arms (Table 1). Subjects were primarily men (85%), with a median age of 38 years. The ethnicity of subjects was diverse: 37% Black, 36% Hispanic, and 23% White. Most subjects reported never used injection drugs. The baseline median CD4 count was 29 cells/mm^3^, 70% of subjects entered the study with a CD4 count of <50 cells/ml. The baseline plasma log_10_ HIV VL was 5.07 copies/mL. Over 90% of subjects were ART naïve.

**Table 1:** Baseline Demographic and Clinical Characteristics by Strategy Arm*.

Characteristic	Total	Early	Deferred
Number	282	141	141
Men (%)	241 (85)	120 (85)	121 (86)
Women (%)	41 (15)	21 (15)	20 (14)
Black (%)	103 (37)	51 (36)	52 (37)
Hispanic (%)	101 (36)	52 (37)	49 (35)
White (%)	64 (23)	29 (21)	35 (25)
Asian (%)	13 (5)	8 (6)	5 (4)
Age [median yrs] (IQR)	38 (32–44)	39 (33–44)	38 (32–44)
IDU never (%)	246 (87)	124 (88)	122 (87)
CD4 (cells/mm^3^) Median (IQR)	29 (10–55)	31 (12–54)	28 (10–56)
HIV RNA (log10) Median (IQR)	5.07 (4.71–5.63)	5.07 (4.74–5.59)	5.08 (4.64–5.64)
No Prior ART	259 (92)	131 (93)	128 (91)
PCP	177 (63)	88 (62)	89 (63)
BI	34 (12)	17 (12)	17 (12)
Other OI	71 (25)	36 (26)	35 (25)
Cryptococcus	35 (12)	13 (9)	22 (16)
Toxoplasmosis	13 (5)	9 (6)	4 (3)
Histoplasmosis	10 (4)	7 (5)	3 (2)
CMV	6 (2)	4 (3)	2 (1)
MAC	6 (2)	3 (2)	3 (2)
Multiple OI/BI (w/in 30 days)	92 (33%)	45 (32%)	47 (33%)

* No statistically significant differences were noted for the various comparisons.

The most common entry OIs included PCP (63%), cryptococcal meningitis (12%), and BIs (12%). (Table 1) One-third of subjects either entered the study with multiple OIs or BIs or developed additional ones within the first 30 days of study entry.

### ART Management

**Figure 2:**
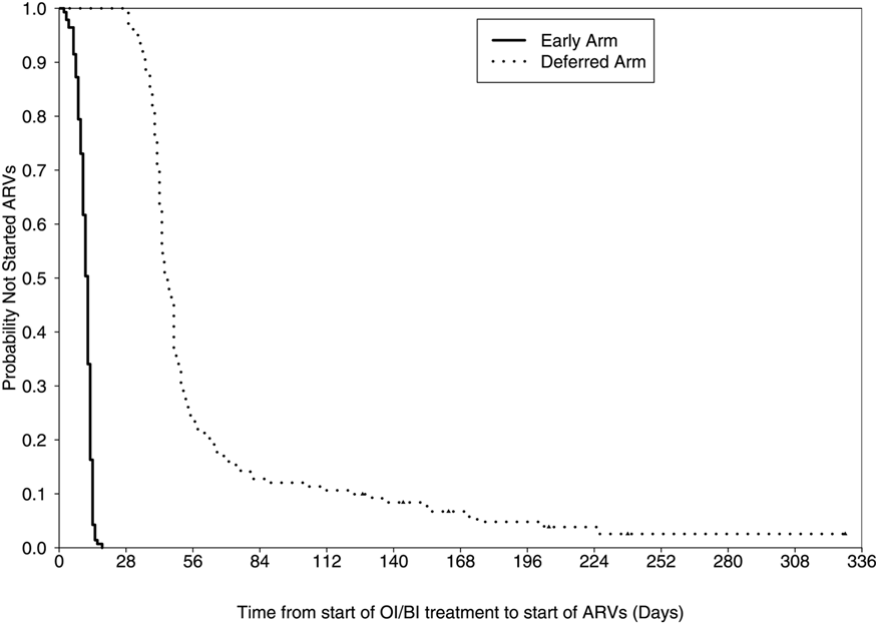
Time to ART initiation from the start of OI/BI treatment. Early arm median 12 days [IQR 9–13 days]; Deferred arm median 45 days [IQR 41–55 days].

All subjects in the early arm and 129 (91%) of subjects in the deferred arm initiated ART. Figure 2 shows that the early arm initiated ART a median of 12 days [IQR, 9–13 days] after OI treatment initiation while the deferred arm started ART a median of 45 days [IQR, 41–55 days] after initiation of OI treatment. The median time to start of ART from randomization was day 0 [IQR 0–1 days] in the early arm vs. day 35 [IQR, 29–44 days] in the deferred arm. There was no overlap in the time to initiation of ART between the study arms – even though the protocol allowed for clinician discretion in ART start times outside of the targeted windows.

There were no significant differences between the arms in initial ART regimens used. A boosted PI with two nRTIs was used in 89% of subjects in the immediate arm and 85% of subjects in the deferred arm while NNRTI-based regimens with two nRTIs were used in 11% and 16%, respectively. Only one subject was prescribed an all nRTI regimen (trizivir+TDF). Lopinavir/r was the boosted PI used in 82% of regimens in the immediate arm and 71% of regimens in the deferred arm. There were somewhat more ART changes in the immediate arm (p = 0.20). The reasons for treatment changes were not different across the two arms (data not shown). At week 48, for subjects on ART –over 90% of subjects who completed questionnaires in both arms reported taking ART within the last four days ‘most’ or ‘all’ of the time, and 59% of subjects in both arms reported never having missed a dose in the prior three months.

### Study Outcomes

Eighty-seven percent of subjects, 123 in each arm, were evaluable for the primary endpoint. (Table 2) There was no statistically significant difference in the ordered 3 category primary endpoint at 48 weeks: AIDS progression/death in 20 (14%) vs. 34 (24%); 54 (38%) vs. 44 (31%) had no progression or death but viral load ≥50 copies/ml; and 67 (48%) vs. 63 (45%) had no progression or death and viral load of <50 copies/ml at week 48 in the early versus the deferred arms, respectively (p = 0.22). Per the study design, the 18 subjects in each arm with no endpoint information were included in the intermediate outcome group. However, excluding these subjects or assigning them to the other categories of outcome did not alter the result (data not shown). Moreover, sensitivity analyses including AIDS defining endpoints that occurred in the first 30 days after study entry did not alter the study results (data not shown).

**Table 2:** Study Efficacy and Safety Outcomes over 48 weeks by Strategy Arm.

Outcome	Total	Early	Deferred	p-value
**No Endpoint Information**	36 (12.8%)	18 (12.8%)	18 (12.8%)	
**Primary Endpoint**				
AIDS Progression/Death	54 (19.1%)	20 (14.2%)	34 (24.1%)	
Plasma Viral Load >50 copies; no progression*	98 (34.8%)	54 (38.3%)	44 (31.2%)	
Plasma Viral Load <50 copies; no progression	130 (46.1%)	67 (47.5%)	63 (44.7%)	
				0.215^a^
**Secondary Endpoints**				
AIDS Progression/Death	54 (19.1%)	20 (14.2%)	34 (24.1%)	0.035^b^
HIV VL % <50 copies at 48 wks (ITT analysis)	143 (51%)	71 (50%)	72 (51%)	0.48^c^
CD4 count at 24 weeks (median change from baseline) (IQR )	+115 (+71–+180)	+118 (+75–+186)	+104 (+66–+171)	0.22^c^
CD4 count at 48 weeks [median change from baseline] (IQR)	+187 (+106–+269)	+187 (+95–+268)	+187 (+124–+271)	0.50^c^
**Safety Outcomes**				
Had at least one ART Switch or interruptions,	104 (39%)	59 (42%)	45 (35%)	0.26^d^
IRIS Confirmed	20 (7.1%)	8 (5.7%)	12 (8.5%)	0.49^d^
Laboratory Adverse Events Grades 2–4	192 (68%)	90 (64%)	102 (72%)	0.16^d^
Clinical Adverse Events Grades 2–4	130 (46%)	61 (43%)	69 (50%)	0.40^d^
Subjects with Hospitalization	106 (38%)	55 (39%)	51 (36%)	0.71^d^
Median Hospital Days (among hospitalizations)	5 (2–10)	5 (2–10)	6 (2–10)	0.79^c^

* Includes subjects with missing outcomes.

a Stratified Wilcoxon Rank Sum test.

b Stratified exact test.

c Wilcoxon Rank Sum.

d Fisher's Exact Test.

**Figure 3:**
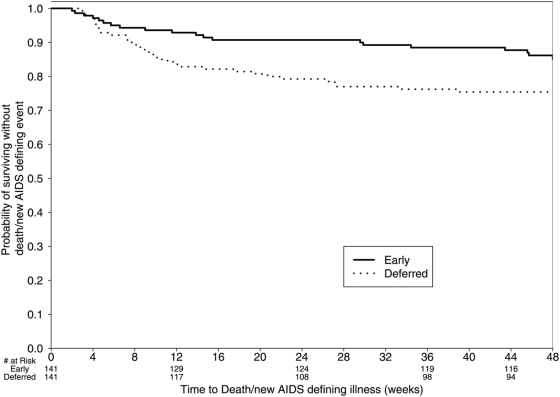
Time to AIDS progression or death. HR = 0.53 Early versus Deferred ART [95%CI 0.30–0.92 p = 0.023].

Importantly, a difference in clinical outcomes between the study arms was observed in a secondary analysis that evaluated the rates of AIDS progression or death. Twenty subjects (14.2%) in the early arm died or had progression of disease compared to 34 subjects (24.1%) in the deferred arm (stratified p = 0.035; OR = 0.51; 95%CI: 0.27–0.94). Furthermore, time to AIDS progression/death also favored the early ART arm (p = 0.02; HR = 0.53; 95%CI: 0.30–0.92). (Figure 3) AIDS progression events were, for the most part, significantly morbid AIDS-related conditions, and deaths were primarily AIDS-related opportunistic infections or malignancies. (Table 3) The differences between the study arms were maintained when AIDS-defining events within the first 30 days of randomization were included as endpoints (data not shown).

**Table 3:** Death and AIDS Progression Events (after day 30) by Strategy Arm.

AIDS Diagnosis	Early ART		Deferred ART	
	Fatal	Non-Fatal	Fatal	Non-Fatal
Esophageal Candidiasis	0	2	0	3
Cryptococcal meninigitis	0	1	2	1
Disseminated Histoplasmosis	1	0	0	2
Invasive Aspergillosis	0	0	1	0
CMV	0	1(1)	0	3(2)
KS	0	1	0	1
Lymphoma	4	0	0	0
Disseminated MAC (or other atypical mycobacterial infection)	0	3	3	3(1)
TB	1	2	0	3
PCP	1	0	3	1(1)
Pneumonia, recurrent	0	1	0	2(1)
Toxoplasmosis or Cryptosporidiosis	0	0	0	2
Wasting Syndrome	0	0	0	1
Sepsis	0	0	3	0
Other*	1	1	1	1
No Information	2	0	1	0
TOTAL Events	10	12(1)	14	23(5)
TOTAL Subjects	10	10	14	20

* Fatal events: Cocaine Intoxication @ week 54 in Immediate arm, Cirrhosis death week 21 in deferred; Non-fatal events 1 case of HSV >1 mo in each study arm.

Events in parentheses are additional non-fatal events which occurred in people who died.

**Figure 4:**
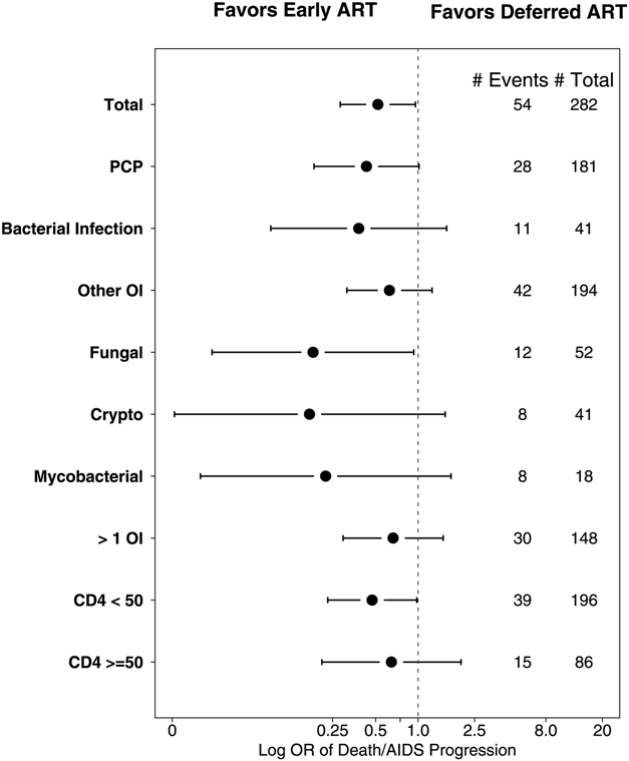
AIDS Progression/Death by entry diagnoses given as log Odds ratios (with 95%CI) with OR <1.0 favoring early versus deferred ART. Total, fungal and CD4<50 categories represent significance at p<0.05. (Fungal Infections include cryptococcal infections and histoplasmosis).

Clinical events by study arm were evaluated by confirmed entry diagnoses in a post-hoc analysis. (Figure 4). Entry diagnoses were confirmed independently by two study team members after the study was closed and all required clinical data was completed and submitted by the sites. Since subjects could have more than 1 confirmed OI/BI, some subjects were included in more than one category. The figure reveals that for all categories of confirmed diagnoses early ART appears to be associated with less clinical progression (i.e. OR <1.0) compared to deferred ART. However, many of the categories lack appropriate power to be reach a standard level of statistical significance but we see statistically significant results for overall, fungal infections (includes cryptococcal infections and histoplasmosis) and those entering with CD4 count of less than 50 cells/mm^3^.

**Figure 5:**
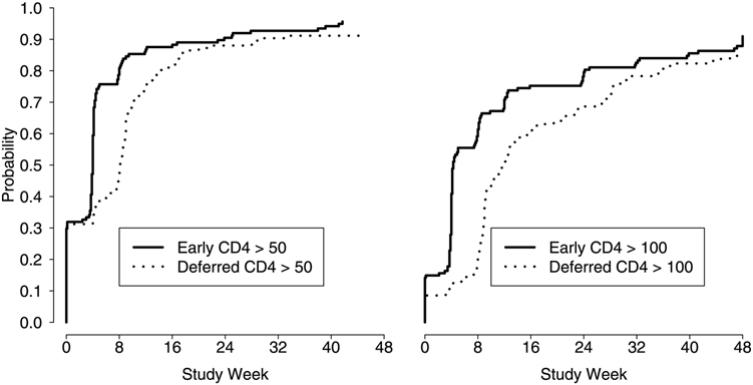
Time to CD4>50 cells/mm3; Early ART median time 4.0 weeks (IQR 0–5.0 weeks) versus deferred ART median time 8.1 weeks (IQR 0–12.7 weeks). Right side graph: Time to CD4>100 cells/mm3; Early ART median time 4.3 weeks (IQR 4.0–23.6 weeks) versus Deferred ART median time 12.1 weeks (IQR 8.6–28.1 weeks) (p<0.001 for both comparisons).

The time to a CD4+ count >50 cells/mm^3^ was 4.0 weeks versus 8.6 weeks and time to CD4+ count greater than 100 cells/mm^3^ was 4.3 weeks vs. 12.0 weeks in the early-versus-deferred arm, respectively (p<0.001 for both comparisons). (Figure 5) However, both arms eventually reached similar CD4+ counts by week 24, and median CD4+ count at week 48 was 220 cells/mm^3^ in the immediate arm compared to 233 cells/mm^3^ in the deferred arm. Furthermore, virologic control (independent of AIDS progression events) was nearly identical in the two arms; 71 immediate ART and 72 deferred ART subjects achieved a viral load of <50 copies/mL at week 48 based on a standard ITT analysis (lost or death by week 48 = fail). (Table 2)

### Adverse Events

The number of subjects reporting moderate or worse (Grades 2, 3 and 4) new signs and symptoms were 14, 40, and 7 subjects in the immediate arm and 34, 29, and 6 subjects in the deferred arm (p = 0.87; Grade 2 reported only for ART regimen change). Table 4 shows the types of signs and symptoms did not differ across the arms. No significant differences were observed in the rates of laboratory abnormalities across the study arms with the exception of more Grade 3 absolute neutrophil count depression in the deferred arm (p = 0.05). (Table 5) Hospitalizations and number of hospital days were also not different in the two arms. (Table 2)

**Table 4:** ACTG A5164 Clinical Adverse Events (New Signs and Symptoms of Grade 2 or Higher over 48 weeks).

	Treatment Group														
	Early (N = 141)					Deferred (N = 141)					All (N = 282)				
	Toxicity Grade					Toxicity Grade					Toxicity Grade				
Toxicities	2	3	4	5	Number subjects	2	3	4	5	Number subjects	2	3	4	5	Number subjects
Any General Body	10	23	2	0	35	17	13	1	0	31	27	36	3	0	66
Any Respiratory	7	8	2	0	17	7	6	2	0	15	14	14	4	0	32
Any Circulatory/Cardiac	3	1	0	0	4	2	3	0	0	5	5	4	0	0	9
Any Hematology	1	1	0	0	2	4	0	1	0	5	5	1	1	0	7
Any Hematology, Signs and Symptoms	1	1	0	0	2	4	0	1	0	5	5	1	1	0	7
Any Liver/Hepatic	1	1	0	0	2	0	0	0	0	0	1	1	0	0	2
Any Gastro-Intestinal	9	9	2	0	20	15	9	0	0	24	24	18	2	0	44
Any Renal	0	1	0	0	1	0	0	0	0	0	0	1	0	0	1
Any Reproductive	3	0	0	0	3	2	1	1	0	4	5	1	1	0	7
Any Skin	7	8	1	0	16	15	5	0	0	20	22	13	1	0	36
Any Neurological	11	13	1	0	25	18	13	4	0	35	29	26	5	0	60
Any Special Senses	2	0	0	0	2	0	2	0	0	2	2	2	0	0	4
Any Other	1	1	1	0	3	2	1	0	0	3	3	2	1	0	6
ANY SIGN/SYMPTOM	14	40	7	0	61	34	29	6	0	69	48	69	13	0	130

Table reflects the number of subjects reporting each symptom/toxicity.

Grade 0 = Normal, 1 = Mild, 2 = Moderate, 3 = Severe, 4 = Life-Threatening, 5 = Death.

The worst grade for each Symptom category is reported.

**Table 5:** A5164 Laboratory Adverse Events (New Laboratory Abnormalities of Grade 2 or Higher over 48 weeks).

	Treatment Group														
	Early (N = 141)					Deferred (N = 141)					All (N = 282)				
	Toxicity Grade					Toxicity Grade					Toxicity Grade				
Toxicities	2	3	4	5	Number subjects	2	3	4	5	Number subjects	2	3	4	5	Number subjects
Any Chemistry	20	10	8	0	38	23	12	5	0	40	43	22	13	0	78
Any Chemistry, General	20	10	8	0	38	23	12	5	0	40	43	22	13	0	78
Any Hematology	13	9	7	0	29	11	22	10	0	43	24	31	17	0	72
Any Hematology, Coagulation	4	1	3	0	8	4	3	1	0	8	8	4	4	0	16
Any Hematology, RBC	4	2	1	0	7	4	4	3	0	11	8	6	4	0	18
Any Hematology, WBC/Differential	5	8	6	0	19	7	18	7	0	32	12	26	13	0	51
Any Metabolic	13	8	3	0	24	19	5	1	0	25	32	13	4	0	49
Any Liver/Hepatic	8	14	5	0	27	23	10	6	0	39	31	24	11	0	66
Any Renal	3	1	2	0	6	4	4	1	0	9	7	5	3	0	15
Any Pancreatic	6	17	0	0	23	11	13	5	0	29	17	30	5	0	52
ANY TOXICITY	31	39	20	0	90	36	45	21	0	102	67	84	41	0	192

IRIS was reported in 23 cases and confirmed in 20: 8 subjects in the immediate arm and 12 in the deferred arm. There was no evidence of an association of IRIS with the entry OI/BI: 13 (65%) IRIS cases were in subjects with PCP who comprised 63% of the study population. IRIS developed a median of 33 days [IQR: 26, 72 days] after initiation of ART. There was no significant difference in the frequency of IRIS between subjects who received corticosteroids during the treatment of their OI and those who did not receive corticosteroids; 9/150 (6%) versus 11/112 (9.8%), respectively (p = 0.35).

## Discussion

This strategy trial did not demonstrate a significant result in the primary outcome: an ordered categorical endpoint at one year that combines clinical and virologic outcomes. This combined outcome was chosen because we did not believe we would see a significant difference in the incidence of AIDS progression and/or death alone given our sample size and best estimates of progression rates in the HAART era. However, because the composite endpoint incorporated an assessment of viral load at 1 year, which was essentially identical in both arms, the differences between the groups were not significant on our primary combined outcome. In effect, the equivalent virologic outcomes at 1 year “diluted” the larger than anticipated positive impact of early ART on clinical outcomes.

There was a significant difference favoring the early treatment group in the secondary outcome of AIDS progression/death. The impact is seen primarily on morbidity and mortality events in the first 6 months (see Figure 3), suggesting that early improvement in immune responsiveness is critical to prevent clinical progression. Subjects in the early ART arm spent less time with CD4 counts <50 or <100 and therefore the “window of vulnerability” to additional AIDS-related complications was shortened by early use of ART. The idea that ART seems to have an early effect on the clinical course following an OI is supported by our finding that early ART was beneficial even though the difference in the time to initiation of ART following the OI was only about 1 month. The difference in outcomes could not be explained by differences in ART regimens used, adherence, adverse events or number of hospitalizations (which were similar in the two arms).

Because AIDS progression/death was not the primary outcome of our study, we recognize that our results should be interpreted cautiously. On the other hand, AIDS progression/death is an important (it could be argued the most important) outcome for ART trials, and our results are consistent with the biologic effects of ART treatment. These findings extend the results of landmark clinical trials that ushered in the first decade of effective ART in patients with advanced immunodeficiency but without active OIs. [4]–[6] In retrospect, one would not expect that a 1 month difference in ART treatment would significantly impact viral load response at 1 year if the patient survives the period immediately following OI without further complications.

Surprisingly, IRIS was uncommon in this study (7%) although subjects had very advanced immunodeficiency, were receiving ART early in their OI treatment course, and had good increases in CD4 counts - all conditions that have been associated with IRIS. From published case series and clinical cohorts, the incidence of IRIS after ART initiation has ranged from 15% to 45% [14]–[19]. It is not clear why the rate of IRIS was low in this trial. However, we do not believe it is because cases were being missed given the special efforts that the study team made to find cases including a team review of all subjects who received corticosteroids that identified no unreported IRIS cases. The use of predefined criteria and real-time review in this prospective study may have reduced case finding bias. In support of this is another recently published prospective study reporting a similar rate of IRIS in a South African population. [20]

Although IRIS has been reported for many AIDS-related OIs, the risk is likely to vary by OI and corticosteroid use. We conclude that IRIS is less frequently a complication of ART in the setting of the OIs included in this study– most particularly PCP - than has been the impression from retrospective studies to date. We don't believe that corticosteroid use influenced the overall incidence of IRIS but may have deferred its onset. However, other infections like TB may have higher rates of IRIS. TB at entry was excluded in this study but is being studied in a second ACTG study. At least for the spectrum of OIs included in this study, fear of IRIS should not be a reason to defer ART.

Initiating ART early required slightly more ART changes but this did not appear to have a deleterious impact on outcomes. On the positive side, starting ART early may better assure that patients have an opportunity to benefit from this life-saving treatment. In this study, all subjects in the early arm initiated ART, but 9% of the subjects in the deferred arm never did. This can be seen as another potential benefit to the strategy of initiating ART early. If ART is deferred there may be a “lost opportunity” to intervene (either because patients become progressively more ill or died or are lost to follow up medical care) and this effect may be even larger in the context of clinical practice as opposed to a clinical trial wherein additional resources are available and additional efforts are made to follow and treat subjects. Individuals who present to care with late stage AIDS without benefit of prior ART are often seen as being at high risk for non-adherence and poor medical follow up. However, we believe that given these study results practitioners should at least offer ART early in the treatment course of the OI to these patients.

An important strength of this strategy study is that it was designed to mirror most aspects of clinical practice. Physicians often treat on the basis of presumptive diagnoses. Had we limited our study to only the treatment of definitive diagnoses, it may not have generalized to the care of patients treated presumptively. Also, we allowed for a range of starting points in ART treatment within both arms because that too reflects the realities of clinical practice. Although AIDS progression/death was not the primary outcome of our study, it is an important standard by which to judge the outcome of ART trials. Our findings most directly reflect the spectrum of opportunistic and serious bacterial infections seen in the resource-rich countries where the majority of the patients were enrolled. These results also reflect a primarily ART naïve population, in patients with prior ART therapy and drug resistance the impact of early ART may be more attenuated. Additional randomized controlled studies are required for TB (ongoing) and cryptococcal infections given our lack of power to provide a definitive result in this study.

Our study can be viewed as demonstrating the efficacy of early ART since there was selection by treating clinicians for patients that were likely to be “good” clinical trial candidates. We have no way to know how much “pre-screening” of patients occurred but treating clinicians were instructed to use “best clinical judgment” in selecting patients for the study. Although there was likely some “pre-selection” of patients, this is no different than any other randomized clinical trial and since there were 40 participating sites across a wide spectrum of clinical settings in the US (and South Africa) we feel the study results are fairly generalizable. Studies of the effectiveness of this strategy in other populations who might be less-ideal clinical trial candidates -like injection drug users - may be required to further generalize our findings but even more challenging to conduct.

We would be cautious to generalize these results to resource-limited countries, even though patients in those countries are accessing ART with advanced AIDS and with some of these same OIs and BIs as seen in our study. None-the–less, it is reassuring that the results favoring early ART seem to be consistent across a variety of clinical categories (Figure 4)

On the basis of these results, and although logistically challenging, we recommend that ART be started early in the setting of acute AIDS-related OIs if there are no major contraindications to doing so. Waiting to complete OI treatment before initiating ART appears to be associated with a higher risk of AIDS-related disease progression and/or death without any significant benefit in terms of safety or virological response. Initiation of ART in the setting of an acute AIDS-related OI will require a multi-disciplinary team approach with expertise in ART management to be sure that care for these critical ill patients is well-coordinated and effective. Although there are many potential clinical challenges in initiating ART early in patients with acute AIDS-related opportunistic infections the potential benefits to such patients appears to be substantial.

## Supporting Information

Appendix S1 A5164 Criteria for AIDS-related Opportunistic Infections or Bacterial Infections
(0.03 MB DOC)

Appendix S2 Supported in part by the AIDS Clinical Trials Group funded by the National Institute of Allergy and Infectious Diseases,” AI38858, and AI68636, and AI68634. Also supported in part by the General Clinical Research Center Units funded by the National Center for Research Resources.
(0.04 MB DOC)

Checklist S1 CONSORT checklist
(0.07 MB DOC)

Protocol S1 Trial Protocol
(0.82 MB DOC)
