# Supplementary material for: Early Antiretroviral Therapy Reduces AIDS Progression/Death in Individuals with Acute Opportunistic Infections: A Multicenter Randomized Strategy Trial
Source: PLoS One. 2009 May 18;4(5):e5575. doi: 10.1371/journal.pone.0005575 (PMC2680972; doi:10.1371/journal.pone.0005575)
Supplement: Appendix S1 — A5164 Criteria for AIDS-related Opportunistic Infections or Bacterial Infections (0.03 MB DOC) [file pone.0005575.s001.doc]

#### APPENDIX I: A5164 CRITERIA FOR AIDS-RELATED OPPORTUNISTIC INFECTIONS OR BACTERIAL INFECTIONS

The definitions below are only to be used for study eligibility, and are not to be used to define events after study entry.

**(Confirmed and probable except as noted)**

- *Pneumocystis jirovecii* pneumonia (PCP)
- Bacterial pneumonia (subjects must also have CD4^+^ count < 200 cells/mm^3^)
- Cryptococcal meningitis (confirmed only)
- Disseminated histoplasmosis
- Disseminated *Mycobacterium avium* complex
- Cytomegalovirus retinitis
- Cytomegalovirus encephalitis
- Toxoplasmic encephalitis
- Other atypical mycobacterial infections (non-tuberculous, non-MAC)
- Other serious, invasive bacterial infections (bacterial infection of deep tissue, body cavity, or other normally sterile site) (accompanied by CD4+ cell count < 200 cells/mm^3^)
- Other serious OIs, including other AIDS-defining and AIDS-related OIs, for which appropriate therapy other than ART exists, with permission of the protocol chair or vice chair.
