# Supplementary material for: Early Antiretroviral Therapy Reduces AIDS Progression/Death in Individuals with Acute Opportunistic Infections: A Multicenter Randomized Strategy Trial
Source: PLoS One. 2009 May 18;4(5):e5575. doi: 10.1371/journal.pone.0005575 (PMC2680972; doi:10.1371/journal.pone.0005575)
Supplement: Appendix S2 — Supported in part by the AIDS Clinical Trials Group funded by the National Institute of Allergy and Infectious Diseases,” AI38858, and AI68636, and AI68634. Also supported in part by the General Clinical Research Center Units funded by the National Center for Research Resources. (0.04 MB DOC) [file pone.0005575.s002.doc]

Appendix

Supported in part by the AIDS Clinical Trials Group funded by the National Institute of Allergy and Infectious Diseases," AI38858, and AI68636, and AI68634. Also supported in part by the General Clinical Research Center Units funded by the National Center for Research Resources.

Other members of the ACTG A5164 team were B. Alston-Smith (Division of AIDS), M. Dorosh (University of Colorado), (Awny Farajallah (Bristol-Myers Squibb), K. Freedberg (Massachusetts General Hospital), H. Gutzman (ACTG Data Management Center), M. Harvey (ACTG Data Management Center), L. Miller (UCLA School of Medicine), M. Poblenz (Gilead Sciences), J. Rooney (Gilead Sciences), P. Sax (Brigham and Women’s Hospital), R. Shafer (Stanford University), R. Stryker (Abbott Laboratories), L. Thompson (University of Miami), K. Tooley (University of Buffalo), P. Tran (Division of AIDS).

#### The following persons and institutions participated in the conduct of this trial: H. Edmondson, L. Mendez (University of Southern California), J. Eron Jr., C. Zelasky (University of North Carolina at Chapel Hill), M. John, C. Firnhaber (University of Witwatersrand), B. Putnam, S. Johnson (University of Colorado Hospital), S. Valle, D. Slamowitz (Stanford University AIDS Clinical Trials Unit), R. Lopez, M. Witt (Harbor-UCLA Medical Center), M. Laverty, R. Hutt (New York University/NYC HHC at Bellevue Hospital Center), D. Havlir, C.B. Hare (University of California San Francisco), K. Tashima, D. Perez (Miriam Hospital/Brown University), C. Fichtenbaum, D. Daria (University of Cincinnati), R. Redfield, C. Davis (University of Maryland), A. Sbrolla, T. Flynn (Massachusetts General Hospital), M. Albrecht, N. Kim Ling (Beth Israel-Deaconess Medical Center), S. Yawtez, J. Gothing (Brigham and Women's Hospital), H. Rominger, M. Goldman (Indiana University), D. McGregor, L. Williams (Northwestern University), O. Adeyemi, J. Depotes (CORE Center), J. Hoffman, S. Cahill (University of California, San Diego), M. Rodriguez, D. Demarco (Washington University in St. Louis), M. Lisgaris, D. Gebhardt (Case Western Reserve University), K. Whitely, Ann Conrad (MetroHealth), R. Pollard, A. Olusanya (University of California, Davis), W. Maher, D. Gochnour (Ohio State University), C. Greisberger, M. Adams, (University of Rochester), M. Palmore, F. Derson (Emory University HIV/AIDS Clinical Trials Unit), M. Fischl, J. Clerville (University of Miami), G. Cox, L. Reed (Duke University), J.Santana, S. Marrero (Puerto Rico) (University of Texas), (Columbia University), (University of Washington, Harborview), K. Henry, B. Bordenave (Hennepin County Medical Center), D. Mildvan, T. Gomez (Beth Israel Medical Center), E.Race, T. Petersen (University of Texas, Southwestern), R.R. MacGregor, K. Maffei (University of Pennsylvania)
