# Supplementary material for: Early Antiretroviral Therapy Reduces AIDS Progression/Death in Individuals with Acute Opportunistic Infections: A Multicenter Randomized Strategy Trial
Source: PLoS One. 2009 May 18;4(5):e5575. doi: 10.1371/journal.pone.0005575 (PMC2680972; doi:10.1371/journal.pone.0005575)
Supplement: Protocol S1 — Trial Protocol (0.82 MB DOC) [file pone.0005575.s004.doc]

# A5164

**A Phase IV Study of Antiretroviral Therapy for HIV-Infected Adults Presenting with Acute Opportunistic Infections: Immediate versus Deferred Antiretroviral Treatment**

**A Multicenter Trial of the AIDS Clinical Trials Group (ACTG)**

**Sponsored by:**

**The National Institute of Allergy and Infectious Diseases**

**Pharmaceutical Support Provided by:**

**Abbott Laboratories**

**Bristol-Myers Squibb**

**Gilead Sciences**

**The ACTG Complications of HIV Disease**

**Research Agenda Committee: Judith A. Aberg, M.D., Chair**

Protocol Chair: Andrew R. Zolopa, M.D.

**Protocol Vice Chair: William Powderly, M.D.**

**DAIDS Clinical Representative: Karen Near, M.D., M.Sc.**

Clinical Trials Specialist: Evelyn Hogg, B.A.

**FINAL Version 3.0**

**August 30, 2005**

TABLE OF CONTENTS

Page

SITES PARTICIPATING IN THE STUDY [5](#__RefHeading___Toc112637346)

PROTOCOL TEAM ROSTER [6](#__RefHeading___Toc112637347)

STUDY MANAGEMENT [9](#__RefHeading___Toc112637348)

GLOSSARY [12](#__RefHeading___Toc112637349)

SCHEMA [14](#__RefHeading___Toc112637350)

1.0 STUDY OBJECTIVES [17](#__RefHeading___Toc112637351)

1.1 Primary Objective [17](#__RefHeading___Toc112637352)

1.2 Secondary Objectives [17](#__RefHeading___Toc112637353)

2.0 INTRODUCTION [18](#__RefHeading___Toc112637354)

2.1 Background [18](#__RefHeading___Toc112637355)

2.2 Rationale [26](#__RefHeading___Toc112637356)

3.0 STUDY DESIGN [30](#__RefHeading___Toc112637357)

4.0 SELECTION AND ENROLLMENT OF SUBJECTS [32](#__RefHeading___Toc112637358)

4.1 Step 1 Inclusion Criteria [32](#__RefHeading___Toc112637359)

4.2 Step 1 Exclusion Criteria [35](#__RefHeading___Toc112637360)

4.3 Step 2 Inclusion Criteria [37](#__RefHeading___Toc112637361)

4.4 Step 2 Exclusion Criteria [37](#__RefHeading___Toc112637362)

4.5 Study Enrollment Procedures [37](#__RefHeading___Toc112637363)

4.6 Coenrollment Guidelines [38](#__RefHeading___Toc112637364)

5.0 STUDY TREATMENT [38](#__RefHeading___Toc112637365)

5.1 Regimens, Administration, and Duration [38](#__RefHeading___Toc112637366)

5.2 Dose Adjustments: Step 1, Arm A and Step 2 Arm B [41](#__RefHeading___Toc112637367)

5.3 Product Formulation and Preparation [43](#__RefHeading___Toc112637368)

5.4 Product Supply, Distribution, and Pharmacy [43](#__RefHeading___Toc112637369)

5.5 Concomitant Medications [44](#__RefHeading___Toc112637370)

5.6 Adherence Assessment [46](#__RefHeading___Toc112637371)

6.0 CLINICAL AND LABORATORY EVALUATIONS [47](#__RefHeading___Toc112637372)

6.1 Schedule of Events [47](#__RefHeading___Toc112637373)

6.2 Timing of Evaluations [49](#__RefHeading___Toc112637374)

6.3 Special Instructions and Definitions of Evaluations [51](#__RefHeading___Toc112637375)

6.4 Off-Study Drug Requirements [58](#__RefHeading___Toc112637376)

7.0 GENERAL PRINCIPLES OF ANTIRETROVIRAL THERAPY MANAGEMENT [59](#__RefHeading___Toc112637377)

7.1 Immune Reconstitution Inflammatory Syndrome [59](#__RefHeading___Toc112637378)

7.2 General Guidelines for Toxicity Management [60](#__RefHeading___Toc112637379)

7.3 Dose Modification Instructions [61](#__RefHeading___Toc112637380)

7.4 Toxicity Management Criteria and Procedures for Modification

of Symptoms [61](#__RefHeading___Toc112637381)

7.5 Toxicity Management for Laboratory Abnormalities [65](#__RefHeading___Toc112637382)

8.0 CRITERIA FOR PREMATURE TREATMENT OR STUDY DISCONTINUATION [69](#__RefHeading___Toc112637383)

8.1 Premature Discontinuation of ART [69](#__RefHeading___Toc112637384)

8.2 **Premature** Study Discontinuation [69](#__RefHeading___Toc112637385)

9.0 STATISTICAL CONSIDERATIONS [70](#__RefHeading___Toc112637386)

9.1 General Design Issues [70](#__RefHeading___Toc112637387)

9.2 Endpoints [70](#__RefHeading___Toc112637388)

9.3 Sample Size and Accrual [71](#__RefHeading___Toc112637389)

9.4 Monitoring [73](#__RefHeading___Toc112637390)

9.5 Analyses [74](#__RefHeading___Toc112637391)

10.0 DATA COLLECTION AND MONITORING AND ADVERSE EXPERIENCE REPORTING [75](#__RefHeading___Toc112637392)

10.1 Records to Be Kept [75](#__RefHeading___Toc112637393)

10.2 Role of Data Management [75](#__RefHeading___Toc112637394)

10.3 Clinical Site Monitoring and Record Availability [76](#__RefHeading___Toc112637395)

10.4 Serious Adverse Experience (SAE) Reporting [76](#__RefHeading___Toc112637396)

11.0 HUMAN SUBJECT**S** [76](#__RefHeading___Toc112637397)

11.1 Institutional Review Board (IRB) Review and Informed Consent [77](#__RefHeading___Toc112637398)

11.2 Subject Confidentiality [77](#__RefHeading___Toc112637399)

11.3 Study Discontinuation [77](#__RefHeading___Toc112637400)

12.0 PUBLICATION OF RESEARCH FINDINGS [77](#__RefHeading___Toc112637401)

13.0 BIOHAZARD CONTAINMENT [77](#__RefHeading___Toc112637402)

14.0 REFERENCES [79](#__RefHeading___Toc112637403)

APPENDIX I: A5164 CRITERIA FOR AIDS-RELATED OPPORTUNISTIC INFECTIONS OR BACTERIAL INFECTIONS [83](#__RefHeading___Toc112637404)

APPENDIX II: GUIDELINES FOR SUBJECT FOLLOW UP [91](#__RefHeading___Toc112637405)

APPENDIX III: PROCEDURES AND INSTRUCTIONS FOR VIROLOGIC AND IMMUNOLOGIC SPECIMENS [93](#__RefHeading___Toc112637406)

APPENDIX IV: SAMPLE INFORMED CONSENT

APPENDIX V: PREGNANCY SAMPLE INFORMED CONSENT

# SITES PARTICIPATING IN THE STUDY

A5164 is open to all ACTG units and subunits and to ICTUs listed on the A5164 protocol-specific web page.

All sites will be asked to maintain a record of the reasons prospective subjects do not enroll in A5164 (see section 3.0).

# PROTOCOL TEAM ROSTER

Protocol Chair

Andrew R. Zolopa, M.D.

**Division of Infectious Diseases**

Stanford University

300 Pasteur Dr., Room S1**69**

Stanford, CA 94305**-5107**

Phone: (650) **354-8107**

FAX: (650) **354-8102**

E-mail: [azolopa@stanford.edu](mailto:azolopa@stanford.edu)

Vice Chair

William G. Powderly, M.D.

**Mater University Hospital**

**Medical Professional Unit**

**44 Eccles Street**

**Dublin, MO 7**

**Ireland**

Phone: **(353) 183-07419**

FAX: **(353) 183-08404**

E-mail: [**bpowderly@mater.ie**](mailto:bpowderly@mater.ie)

DAIDS Clinical Representative

**Karen Near, M.D., M.Sc.**

**TRP, DAIDS, NIAID, CCRB, NIH**

**Room 5111**

**6700-B Rockledge Drive-MSC 7624**

**Bethesda, MD 20892-7624**

**Phone: (301) 435-3770**

**FAX: (301) 402-3171**

**E-mail:** [**knear@niaid.nih.gov**](mailto:knear@niaid.nih.gov)

Clinical Trials Specialist

Evelyn Hogg, B.A.

Social & Scientific Systems, Inc.

ACTG Operations Center

8757 Georgia Avenue, 12th Floor

Silver Spring, MD 20910-3714

Phone: (301) 628-3337

FAX: (301) 628-3302

E-mail: [**ehogg@s-3.com**](mailto:ehogg@s-3.com)

Statisticians

Robert A. Parker, Sc.D.

Statistical and Data Analysis Center

Harvard School of Public Health

651 Huntington Ave. FXB-609

Boston, MA 02115

Phone: (617) 432-3266

FAX: **(617) 432-2843**

E-mail: [rparker@sdac.harvard.edu](mailto:rparker@sdac.harvard.edu)

**Lauren Komarow, M.S.**

**SDAC/Harvard School of Public Health**

**651 Huntington Avenue**

**FXB Room 508**

**Boston, MA 02115**

**Phone: (617) 432-3233**

**FAX: (617) 432-3163**

**E-mail: lkomarow@sdac.harvard.edu**

Data Manager

Carol Suckow, B.S.N.

Complications Section

Frontier Science & Technology

Research Foundation

4033 Maple Road

Amherst, NY 14226-1056

Phone: (716) 834-0900, **x7277**

FAX: (716) 834-8432

E-mail: [suckow.carol@fstrf.org](mailto:suckow.carol@fstrf.org)

DAIDS Pharmacist

Paul Tran, R.Ph.

Pharmaceutical Affairs Branch

DAIDS, NIAID, NIH

Room 4223

6700-B Rockledge Drive, MSC 7620

Bethesda, MD 20892-7620

Phone: (301) 402-0128

FAX: (301) 402-1506

E-mail: [ptran@niaid.nih.gov](mailto:ptran@niaid.nih.gov)

Virologist

Robert Shafer, M.D.

Stanford University

Medical Center, S-156

300 Pasteur Drive

Stanford, CA 94305

Phone: (650) 725-2946

FAX: (650) 723-8596

E-mail: [rshafer@cmgm.stanford.edu](mailto:rshafer@cmgm.stanford.edu)

Investigators

John G. Gerber, M.D.

University of Colorado Health Sciences

Center

4200 East 9^th^ Avenue, Box C237

Denver, CO 80262

Phone: (303) 315-6923

FAX: (303) 315-3272

E-mail: [john.gerber@uchsc.edu](mailto:john.gerber@uchsc.edu)

Loren G. Miller, M.D., M.P.H.

UCLA School of Medicine

Division of Infectious Diseases

Harbor-UCLA Medical Center

1124 W. Carson Street, Box 466

Torrance, CA 90509

Phone: (310) 222-5623

fAX: (310) 782-2016

E-mail: [lgmiller@ucla.edu](mailto:lgmiller@ucla.edu)

Investigators (Cont’d)

Ian M. Sanne, M.D.

University of Witwatersrand

Infectious Diseases Syndicate

Clinical Trial Unit

17 Eton Road, Parktown

Johannesburg, 2193

South Africa

Phone: 27 11 7172810

Other Phone: 27 11 7172813

FAX: 27 11 482 5554

**E-mail:** [imsanne@yebo.co.za](mailto:imsanne@yebo.co.za)

Paul E. Sax, M.D.

Infectious Disease Unit

Brigham and Women’s Hospital

75 Francis Street

Boston, MA 02115

##### Phone: (617) 732-5885

FAX: (617) 732-6829

E-mail: [psax@partners.org](mailto:psax@partners.org)

Field Representative

Leslie Thompson, R.N., B.S.N.

University of Miami School of Medicine

Department of Medicine

Elliot Building, Room 60A

1800 Northwest 10^th^ Avenue

Miami, FL 33136-1013

Phone: (305) 243-3838

FAX: (305) 243-5765

E-mail: [lthomps@gate.net](mailto:lthomps@gate.net)

Laboratory Technologist

Kelly Tooley, MSEd.

University at Buffalo

Pharmacotherapy Research Center

Core Analytical Laboratory

247 Cooke Hall

Buffalo, NY 14260

Phone: (716) 645-3635 x263

FAX: (716) 645-2001

E-mail: [kmtooley@buffalo.edu](mailto:kmtooley@buffalo.edu)

CCG Representative

Michael Dorosh, M.A.

CAB Liaison

University of Colorado Health Sciences

Center

38 West Maple Avenue

Denver, CO 80223-1839

Phone: (303) 777-5737

FAX: (303) 372-5552

E-mail: [orbit38@juno.com](mailto:orbit38@juno.com)

Industry Representatives

Tania George, Pharm.D.

Bristol-Myers Squibb

Virology Medical Affairs

777 Scudders Mill Road

Mail Stop P11-14

Plainsboro, NJ 08536

Phone: (609) 897-5872

FAX: (609) 897-6068

E-mail: [tania.george@bms.com](mailto:tania.george@bms.com)

Industry Representatives (Cont’d)

James F. Rooney, M.D.

Gilead Sciences

333 Lakeside Drive

Foster City, CA 94404

Phone: (650) 522-5708

FAX: (650) 522-5854

E-mail: [jim_rooney@gilead.com](mailto:jim_rooney@gilead.com)

Stephen R. Smith

Gilead Sciences

Medical Affairs

333 Lakeside Drive

Foster City, CA 94404

Phone: (650) 522-6330

FAX: (650) 522-5782

E-mail: [stephen_smith@gilead.com](mailto:stephen_smith@gilead.com)

Richard Stryker, M.D., M.P.H.
Abbott Laboratories
2738 Westshire Drive
Los Angeles, CA 90068
Phone:
FAX: (323) 856-5814

E-mail: [Richard.stryker@abbott.com](mailto:Richard.stryker@abbott.com)

Laboratory Data Coordinator

Nancy Webb, M.S.

Frontier Science & Technology Research

Foundation, Inc.

4033 Maple Road

Amherst, NY 14226-1056

Phone: (716) 834-0900, x227

FAX: (716) 834-8432

E-mail: [webb.nancy@fstrf.org](mailto:webb.nancy@fstrf.org)

# STUDY MANAGEMENT

All questions concerning this protocol should be sent via e-mail to [actg.teamA5164@fstrf](mailto:actg.teamA5164@fstrf).org. The appropriate team member will respond via e-mail with a “cc” to the team. A response should generally be received within 24 hours (Monday-Friday).

Protocol email group: Sites registering to this study should contact the Computer Support Group at the Data Management Center via e-mail [[actg.user.support@fstrf.org](mailto:actg.user.support@fstrf.org) **for sites in the U.S. (ACTUs**) **or** [**aactg.support@fstrf.org**](mailto:aactg.support@fstrf.org) **for sites outside the U.S. (ICTUs)]** to have the relevant personnel at the site added to the actg.protA5164 e-mail group as soon as possible. Inclusion in the protocol e-mail group will ensure that sites receive important information about the study during its implementation and conduct.

Clinical Medical Management

For questions concerning clinical medical management, including entry criteria, toxicity management, concomitant medication, and co-enrollment, the protocol chair/ vice chair will respond.

- Send an e-mail message to [actg.teamA5164@fstrf.org](mailto:actg.teamA5164@fstrf.org) (ATTN: Andrew Zolopa and William Powderly).
- Include the protocol number (A5164), patient identification # (PID), and a brief relevant history.

For questions specifically related to virologic laboratory tests, the protocol virologist will respond.

- Send an e-mail message to [actg.teamA5164@fstrf.org](mailto:actg.teamA5164@fstrf.org) (ATTN: Robert Shafer, Virologist).
- Include the protocol number (A5164), PID, and a detailed question.

Nonclinical Questions

For nonclinical questions about inclusion/exclusion criteria, the case report forms (CRF), CRF schedule of events, randomization/registration, transfers, delinquencies, and other data management issues, the data managers will respond.

- Send an e-mail message to [actg.teamA5164@fstrf.org](mailto:actg.teamA5164@fstrf.org) (ATTN: Carol Suckow).
- Include the protocol number (A5164), PID, and a detailed question.

Randomization Questions

For randomization questions or problems and SID lists, the SDAC/DMC programmers will respond.

- Call the SDAC/DMC Randomization Desk at (716) **898-7301** or
- E-mail [**sdac.random.desk@fstrf.org**](mailto:sdac.random.desk@fstrf.org) **(ICTUs should use** [**aactg.support@fstrf.org**](mailto:aactg.support@fstrf.org)**).**

Computer and Screen Problems

For computer and screen problems, the SDAC/DMC programmers will respond.

- Send an e-mail message to [actg.user.support@fstrf.org](mailto:actg.user.support@fstrf.org) **(ICTUs should use** [**aactg.support@fstrf.org**](mailto:aactg.support@fstrf.org)**) or**
- **Call (716) 834-0900 x7302**

Protocol Questions

For protocol document questions, the clinical trials specialist will respond.

- Send an e-mail message to [actg.teamA5164@fstrf.org](mailto:actg.teamA5164@fstrf.org) (ATTN: Evelyn Hogg).

Copies of the Protocol

To request copies of the protocol:

- Hard copies: Send an e-mail message to [**ADULT.OPS@fstrf.org**](mailto:ADULT.OPS@fstrf.org) (ATTN: Diane Delgado).
- Electronic copies can be downloaded from the Members area of the ACTG Web site ([http://aactg.s-3.com](http://aactg.s-3.com/)).

Protocol Registration (ICTUs only)

A preliminary review for completeness of an ICTU’s registration packet will be performed at the ACTG Operations Center. ICTUs must send their registration packet via e-mail to [ICTUprotocol@s-3.com](mailto:ICTUprotocol@s-3.com) or call the international program coordinator at the ACTG Operations Center, (301) 628-3474, with any questions about this preliminary review. The original protocol registration packet will be submitted to the DAIDS Regulatory Compliance Center (RCC) for approval within 24 hours of receipt at the ACTG Operations Center, unless omissions are noted during the review.

Protocol Registration (ACTUs only)

For protocol registration:

- Send an e-mail message to [protocol@tech-res.com](mailto:actg.protreg@fstrf.org) **or**
- **Call (301) 897-1707**

**Registration Approval Questions**

- **Send an e-mail message to** [**protocol@tech-res.com**](mailto:protocol@tech-res.com) **or**
- **Call (301) 897-1707**

Study Drug Questions

For questions or problems regarding study drug, dose, supplies, records, or returns, contact Paul Tran, Protocol Pharmacist:

- **Phone** (301) 496-8213 **or**
- **E-mail** [**ptran@niaid.nih.gov**](mailto:ptran@niaid.nih.gov)

Antiretroviral Drug Information

To request copies of antiretroviral drug package inserts or investigator brochures:

- Contact the RCC Safety Information Center at [**RIC@tech-res.com**](mailto:RIC@tech-res.com).

Ordering study-provided drug

- Call the Clinical Research Products Management Center at (301) 294-0741.

Serious Adverse Experiences (SAE)
For SAE questions, contact DAIDS through the RCC Safety Office at:

- Send an e-mail message to [RCCSafetyOffice@tech-res.com](mailto:RCCSafetyOffice@tech-res.com) or
- **Fax: 1-301-897-1710 or 1-800-275-7619**
- Call 1-800-537-9979 **or 301-897-1709**

Any phone calls must be documented by e-mail to [**actg.teamA5164@fstrf.org**](mailto:actg.teamA5164@fstrf.org). This will be the site’s responsibility.

General information concerning study management of ACTG studies can be found on the ACTG WEB page at [http://aactg.s-3.com](http://aactg.s-3.com/).

Additional protocol-related information is located on the A5164 protocol-specific Web page ([*http://aactg.s-3.com/members/ps/5164/ps5164.htm*](http://aactg.s-3.com/members/ps/5164/ps5164.htm)).

# GLOSSARY

AE: adverse experience

ART: antiretroviral therapy or treatment

ARV: antiretroviral (drug/s)

BI: bacterial infection

Deferred ART: ART that is initiated between week 4 and week 32 after enrollment to A5164.

Immediate ART: ART that is initiated within 48 hours after enrollment to A5164 and within 14 days after initiating treatment for the presenting opportunistic infection or bacterial infection.

IRIS: immune reconstitution inflammatory syndrome, defined by the following:

- evidence of an increase in CD4+ cell count and/or a decrease in the HIV-1 viral load in response to starting ART

AND

- symptoms that are consistent with an infectious/inflammatory condition and temporally related to initiation of ART

AND

- - symptoms that cannot be explained by a newly acquired infection, the expected clinical course of a previously recognized infectious agent, or the side effects of ART itself.

OI: any 1999 CDC AIDS-defining condition (MMWR, March 12, 1999) or treatable AIDS related OI, with chair or vice chair approval

OI/BI: first treated opportunistic infection or serious bacterial infection, as specified in Appendix I, which leads to enrollment in the study

PSWP: protocol-specific Web page

Study-provided drugs: lopinavir/ritonavir (LPV/RTV), emtricitabine/tenofovir DF (FTC/TDF), and stavudine (d4T)

Study-recommended drug**s**: lamivudine (3TC) and emtricitabine (FTC)

Virologic failure: virologic failure is defined as any of the following:

1. failure to decrease 1 log by 8 weeks after initiation of **ART**. (Confirmed within 2-4 weeks after the value obtained after 8 weeks of **ART**.)

OR

1. an increase of 1 log from nadir at any time in the study after 8 weeks of **ART**. (Confirmed within 2-4 weeks after the first elevated value.) (See the A5164 Protocol Specific Web Page [PSWP] for instructions on calculating the change in viral load.)

OR

1. failure to achieve < 500 copies/mL after 24 weeks of **ART**. (Confirmed within 2-4 weeks after the value obtained after 24 weeks of therapy.)

# SCHEMA

A5164

A Phase IV Study of Antiretroviral Therapy for HIV-Infected Adults Presenting with Acute Opportunistic Infections: Immediate versus Deferred Antiretroviral Treatment

DESIGN: A5164 is a randomized, phase IV study of the strategy of immediate versus deferred antiretroviral (ARV) therapy or treatment (ART) in subjects with advanced immunodeficiency syndrome who are presenting with acute AIDS-related opportunistic infection**s** (OIs) or serious bacterial infections (BIs). The OI/BIs included are defined in Appendix I with specific criteria used to determine study eligibility.

There will be two steps and two arms in this study. All subjects will enter Step 1 within 14 days after starting therapy for the acute OI/BI and be randomized to receive ART immediately (“immediate” group or Arm A) or to have ART deferred (“deferred” group or Arm B).

Step 2: Subjects in Arm A will not enter Step 2. Subjects in Arm B will enter Step 2 when they are about to initiate ART. Although the initiation of ART may be between week 4 (day 28) and week 32 (day 224) for subjects in Arm B, very strong consideration should be given to starting ART between week 6 (day 42) and week 12 (day 84).

All subjects, in either arm, will be followed **for** 48 weeks, regardless of whether they start ART or whether they are still receiving ART.

The primary endpoint will be a combined endpoint of survival, AIDS progression, and virologic control at week 48. The success of the ART will be evaluated by virologic and immunologic response at weeks 12 and 24, and 24 weeks after initiation of ART. The success of the OI/BI treatment will be evaluated by duration of hospitalization, complication rates, adverse events, and serious toxicities that occurred over the course of treatment.

DURATION: 48 weeks

SAMPLE SIZE: 282 **(**141 subjects per arm**)**

POPULATION: Male and female subjects, at least 13 years old, presenting with an acute AIDS-defining OI or serious BI for which effective antimicrobial therapy is available and prescribed (see Appendix I for a list of eligible OI/BIs and their definition for purposes of enrollment). Subjects with BI must have a CD4+ count <200 cells/mm^3^.

STRATIFICATION: Subjects will be stratified according to CD4^+^ cell count at screening as follows: CD4^+^ count < 50 cells/mm^3^ versus ≥ 50 cells/mm^3^.

Subjects will be stratified on the basis of the first treated OI/BI of the current presentation. Stratification by OI/BI will be as follows:

- PCP
- All BIs (as defined in Appendix I)
- All other OIs (as defined in Appendix I)

REGIMEN: Step 1

All subjects enter Step 1 when they are enrolled in the study. They are randomized either to Arm A (immediate ART) or Arm B (deferred ART).

Arm A (Immediate Treatment):

For Arm A, ART should be initiated within 48 hours after randomization, and within 14 days after the start of treatment for the OI/BI.

Arm B (Deferred Treatment):

For Arm B, no study-provided drugs are provided in Step 1.

Step 2

Subjects in Arm A will not enter Step 2. Subjects randomized to Arm B in Step 1 will enter Step 2 when they initiate ART between week 4 (day 28) and week 32 (day 224). If subjects in Arm B initiate ART outside of this timeframe, they will NOT register to Step 2 and will not receive study-provided drugs, but will continue follow-up on Step 1 through week 48.

Study-provided drugs

The study-provided drugs that will be available for constructing an ARV regimen are:

- Lopinavir/ritonavir (LPV/RTV)
- Emtricitabine/tenofovir disoproxil fumarate (FTC/TDF)
- Stavudine (d4T)

See section 5.2.1 for d4T dose adjustments to be made based on creatinine clearance values.

Study-provided drugs will be offered to subjects on Arm A in Step 1 and to Arm B subjects when they enter Step 2. Although the choice of a third or fourth ARV drug (other than ddI or ZDV if d4T is included in the regimen) is at the discretion of the site investigator, either lamivudine (3TC) **or** emtricitabine (FTC) is strongly recommended as the third ARV drug in an LPV/RTV plus d4T regimen. Substitutions for any of the study-provided drugs and/or additions can be made on a case-by-case basis using FDA-approved drugs and at the discretion of the site investigator. However, only LPV/RTV, FTC/TDF, and d4T will be supplied by the study.

## 1.0 STUDY OBJECTIVES

### 1.1 Primary Objective

To compare in subjects presenting with an acute opportunistic infection (OI) or severe bacterial infection (BI) the outcome of immediate ART versus deferred ART, with outcome at week 48 classified into one of three ordered categories:

1. Survival without AIDS progression with an undetectable plasma HIV-1 viral load (<50 copies/mL).

2. Survival without AIDS progression with detectable plasma HIV-1 viral load (≥ 50 copies/mL).

3. AIDS progression and/or death.

### 1.2 Secondary Objectives

- - 1. Compare the efficacy and effectiveness of ART in subjects in the immediate ART arm versus the deferred ART arm as measured by viral load and CD4+ count over time, and treatment changes.
    2. Compare efficacy of ARV regimen in the two groups as percent < 50 copies/mL at 24 weeks after initiating ART (as opposed to 24 weeks **after** study entry).
    3. Compare the clinical outcomes in subjects in the immediate ART arm versus the deferred ART arm.
    4. Compare safety and tolerability of ART between the two arms to include adverse events, dose-limiting toxicities, and treatment/dose changes related to managing drug toxicities.
    5. Evaluate the incidence and evolution of HIV drug resistance over 48 weeks.
    6. Evaluate quality of life and functional status outcomes at week 48.
    7. Evaluate resource use over the 48-week study period.
    8. Evaluate the impact of self-reported adherence to ART on primary outcome measures.
    9. Compare the incidence of immune reconstitution inflammatory syndrome and the immune correlates in the two arms.

## 2.0 INTRODUCTION

### 2.1 Background

Beginning in the mid-1990s, there has been remarkable progress in the treatment of HIV-1 infection. As a result of new potent combinations of antiretroviral drugs, HIV-infected individuals are living longer and healthier lives. What was once a uniformly fatal disease is now emerging as a chronic manageable infection. The clinical benefits of so-called highly active antiretroviral therapy (“HAART”) regimens have been conclusively demonstrated for patients who initiate HAART relatively late in the HIV disease progression. In fact, it is in relatively advanced disease groups with advanced immunodeficiency in whom the salutary effects of HAART on AIDS progression and preventing death have been most clearly demonstrated [1, 2].

Despite these impressive advances in the medical management of HIV-1 infection, there are many HIV-infected adults who enter into care relatively late in their disease course. In fact, this trend seems to be increasing as the epidemic moves into populations that traditionally have had poor access to health care services such as communities of color, young adults, and poor rural and inner-city dwellers [3]. In many centers, an increasing percentage of patients first present with HIV infection when they are admitted to the hospital with acute OIs such as *Pneumocystis* pneumonia or cryptococcal disease.

The HAART regimens **to be** used for this trial are standard FDA-approved therapies. The following are recommended for the initial antiretroviral regimen, but additional drugs or substitutions with other FDA-approved drugs can be made in the ARV regimens for any of the study-provided drugs (lopinavir/ritonavir [LPV/RTV], emtricitabine/tenofovir disoproxil fumarate [FTC/TDF], and stavudine [d4T]) or study-recommended drug (lamivudine [3TC] or emtricitabine [FTC]) based on the judgment of the site investigator on a case-by-case basis.

Lopinavir/ritonavir (LPV/RTV) (KALETRA**®**)

LPV (formerly ABT-378) is a potent in vitro inhibitor of HIV-1 protease [4]. As a commercial preparation, it is co-formulated with RTV in a 133**.3**-mg LPV/33**.3**-mg RTV ratio. The addition of RTV to LPV significantly impairs clearance of LPV by CYP3A [5], results in 50x higher C_min_ concentrations than LPV alone, and allows for BID dosing.

The favorable in vivo effects of LPV/RTV in combination with reverse transcriptase inhibitors have been demonstrated [6, 7]. A regimen of LPV/RTV, efavirenz (EFV), and a new nucleoside was studied in non-nucleoside reverse transcriptase inhibitor (NNRTI)-naïve patients who had failed two or more protease inhibitor (PI)-containing regimens [6]. At 24 weeks, 69% and 82% (intent-to-treat [ITT]) and 80% and 96% (as treated) of patients randomized to 400 mg/100 mg or 533 mg/133 mg of LPV/RTV, respectively, achieved HIV-1 RNA < 400 copies/mL. The regimens were well tolerated. While the study design makes it impossible to assess the independent contributions of the LPV/RTV and EFV, this degree of suppression is greater than other studies of PI salvage therapy with combinations of dual PIs, an NNRTI, and nucleoside reverse transcriptase inhibitor (NRTI)(s) [8, 9, and ACTG 398]. Although the viral resistance data from this LPV study showed decreased rates of viral suppression in patients with increasing levels of phenotypic resistance to LPV, HIV-1 RNA < 400 copies/mL was achieved in 78%, 67%, and 50% of patients with HIV-1 having 10-20-fold, 20-40-fold, and > 40-fold increase, respectively, in LPV IC_50_ versus wild-type [10]. It is important to investigate whether this phenomenon of viral suppression in the face of decreased phenotypic susceptibility can be achieved with LPV or other PIs in patients who have had prior NNRTI treatment.

Reference should be made to the most recent Kaletra package insert for complete information relating to possible adverse reactions and toxicities.

Emtricitabine and Tenofovir Disoproxil Fumarate Fixed Dose Combination Tablet (FTC/TDF) (Truvada®)

Gilead Sciences has developed Truvada, a new product containing emtricitabine (FTC) 200 mg and tenofovir disoproxil fumarate (TDF) 300 mg in a fixed dose combination (FDC) tablet formulation. A New Drug Application (NDA) for the FDC was filed with the U.S. FDA on March 12, 2004, and was approved on August 2, 2004. As a component of the NDA, two phase I studies evaluating the pharmacokinetics (PK) of coadministered emtricitabine and tenofovir DF tablet formulation have been completed.

Clinical Pharmacokinetics

Study FTC-114 was a 21-day, open-label, randomized, three-way crossover study to evaluate the steady-state PK of FTC (200 mg capsules given once-daily for 7 days) and tenofovir (administered as TDF tablets 300 mg once-daily for 7 days) when administered alone and together in healthy volunteers [16]. A total of 19 healthy volunteers received in a randomized order each of the following three 7-day treatments over a 21-day treatment period:

- - Treatment A: FTC 200 mg QD x 7 days
  - Treatment B: TDF 300 mg QD x 7 days
  - Treatment C: FTC 200 mg + TDF 300 mg QD x 7 days

Drug was administered 30 minutes after a standardized breakfast on days 1, 5, 6, and 7 of each treatment period. Plasma PK parameters at steady-state were assessed over a 24-hour dosing interval following the last dose of each treatment using a non-compartmental analysis. Results based on 17 participants who completed the study showed that FTC had no effect on the PK of tenofovir when administered with TDF on the basis of statistical analyses of steady state AUC, C_max_, and C_min_ values. Tenofovir DF also had no clinically significant effect on the PK of FTC on the basis of AUC, C_max_, and C_min_ values. Although C_min_ of FTC increased approximately 20% with TDF coadministration, AUC and C_max_ were not affected. FTC and TDF were generally well tolerated when administered alone or together for periods of up to 7 days in this healthy volunteer population.

Bioequivalence and Food Effect

Study GS-US-104-172 was a phase I, 28-day, randomized, four-way crossover, PK study in healthy volunteers designed to evaluate the bioequivalence of the FTC/TDF combination tablet compared with the FTC capsule and TDF tablet administered concurrently and also the effect of food (high-fat meal and light meal) on PK [17]. A total of 44 healthy volunteers received in a randomized order each of the following single treatments either in a fasted state, high-fat meal, or light meal over a 28-day treatment period; drug was administered on days 1, 8, 15, and 22 with a 7-day washout period separating study treatments:

- - Treatment A: Single 200 mg FTC capsule co-administered with a single 300 mg TDF tablet administered under fasting conditions
  - Treatment B: FTC 200 mg/TDF 300 mg combination tablet administered under fasting conditions
  - Treatment C: FTC 200 mg/TDF 300 mg combination tablet administered under fed (high-fat meal) conditions
  - Treatment D: FTC 200 mg/TDF 300 mg combination tablet administered under fed (light meal) conditions

Plasma PK parameters at steady-state were assessed over a 48-hour dosing interval following the last dose of each treatment using a non-compartmental analysis. Results based on 39 participants who completed the study revealed the ratios for both the rate and extent (C_max_ and AUC) of tenofovir bioavailability after its administration as TDF or as the combination tablet were contained within the bounds of 80% and 125%, demonstrating bioequivalence of tenofovir between the two treatments. Similarly, bioequivalence was demonstrated between the FTC capsule and the FTC/TDF combination tablet. The 90% confidence intervals for the geometric least squares means ratios of FTC C_max_ and AUC for FTC capsules and the combination tablet were contained within the 80% and 125% interval [17].

Administration of the FTC/TDF combination tablet after either a high-fat or a light meal was associated with a delay in the time to maximum plasma tenofovir concentration (T_max_) relative to T_max_ for the fasted state. Intake of a high-fat meal (784 kcal and 58% fat) or a light meal (373 kcal and 20% fat) resulted in an altered PK profile for tenofovir. The C_max_ of tenofovir increased by approximately 16% and 13.5%, respectively, compared with the fasted-state administration. Likewise, an increase of approximately 35% or 34% in tenofovir AUC_0-∞_ was observed after administration of a high-fat or light meal, respectively, compared with the fasted state. Emtricitabine PK parameters after ingestion of food (high-fat or light meal) were essentially the same as those for the fasting state, and the 90% confidence intervals for the ratios of treatment geometric means for C_max_ and AUC_0_-∞ after either a high-fat or light meal were contained within 80% to 120%, indicating no food effect on the PK profile of FTC.

Overall, study GS-US-104-172 demonstrated bioequivalence between the FTC/TDF combination tablet and the FTC capsule and TDF tablet formulations when administered separately. Administration of the FTC/TDF combination tablet with either a high-fat meal or light meal increased tenofovir exposure by approximately 30% compared with fasted-state administration. Clinical experience with TDF indicates that the effect of food on tenofovir exposure is not of clinical relevance. FTC and TDF, either administered as a combination tablet (FTC 200 mg/TDF 300 mg) or coadministered as FTC 200-mg capsule and TDF 300-mg tablet, were well tolerated.

Clinical Studies

Study GS-01-934 is an ongoing, phase III, multicenter, randomized, open-label, active controlled study designed to compare the dual-NRTI backbones of FTC 200 mg QD and TDF 300 mg QD to Combivir® (ZDV/3TC) BID both in combination with EFV in ARV-naïve participants with baseline HIV-1 RNA >10,000 copies/mL [18]. The primary efficacy endpoint of this study is achievement and maintenance of confirmed HIV-1 RNA < 400 copies/mL through week 48, as defined by the U.S. FDA time-to-loss-of-virologic-response (TLOVR) algorithm. The ITT population (n=509) had a mean age of 38 years, mean HIV-1 RNA of 5.0 log_10_ copies/mL, mean CD4+ cell count of 246 cells/mm^3^, and 86% were male. Using the TLOVR algorithm, 87% in the FTC/TDF group (n=255) versus 78% in the Combivir group (n=254) achieved HIV-1 RNA < 400 copies/mL through week 24, (95% CI: +1.9%,+14.9%, p=0.01). Similarly, 73% in the FTC/TDF group versus 65% in the Combivir group achieved HIV-1 RNA < 50 copies/mL through week 24 (95% CI: +0.5%,+16.2%, p=0.038). More participants with baseline HIV-1 RNA > 100,000 copies/mL achieved HIV-1 RNA < 50 copies/mL in the FTC/TDF group (67%) versus the Combivir group (54%) (95% CI: +2.9%,+25.6%, p=0.021). Mean changes from baseline to week 24 in CD4+ cell counts were 129 and 111 cells/mm^3^ for the FTC/TDF and Combivir groups, respectively.

Resistance testing results were available in 18 participants (FTC/TDF, n=10; Combivir, n=8) at week 24. EFV resistance and M184V/I developed similarly in both treatment groups, no participants developed K65R or thymidine analogue mutations.

The most common AEs in the FTC/TDF group were dizziness, nausea, and diarrhea, while in the Combivir control group the most frequent AEs were nausea, dizziness, and insomnia. Compared with the Combivir group, the FTC/TDF group had a ≥ 5 % higher incidence of diarrhea (18% vs. 10%) and nasopharyngitis (12% vs. 5%). Compared with FTC/TDF group, the Combivir group had a ≥ 5 % higher incidence of nausea (22% vs. 30 %), vomiting (4% vs.10%), and anemia (<1% vs. 7%). AEs leading to study regimen discontinuation (most common: anemia, nausea, fatigue, vomiting) were fewer in the FTC/TDF group (3%) versus Combivir group (9%), p=0.008. There were no clinically significant renal events in the FTC/TDF group during 24 weeks of treatment. The overall incidence and severity of graded toxicities for renal laboratory parameters were similar between the 2 treatment groups.

Study M02-418 is a phase III, randomized, open-label, multicenter study designed to compare LPV 800 mg/RTV 200 mg QD vs. LPV 400 mg/RTV 100 mg BID with the background regimen of FTC 200 mg QD and TDF 300 mg QD in ARV-naïve participants with HIV-1 RNA >1000 copies/mL [19-21]. A total of 190 participants between the ages of 19-75 years were enrolled, 115 to the QD arm and 75 to the BID arm. At baseline, the median HIV-1 RNA levels for participants in the QD and BID arms were 4.8 and 4.6 log_10_ copies/mL and the median CD4+ cell counts were 214 and 232 cells/mm^3^, respectively. Overall, approximately 45% of participants had CD4+ cell count <200 cells/mm^3^ and 38% had HIV-1 RNA >100,000 copies/mL. At Week 48, based on the ITT (NC=F) analysis, 70% of participants in the QD regimen demonstrated HIV-1 RNA <50 copies/mL, compared with 64% of those in the BID group (95% CI: -7%; 20%). In addition, increases in CD4+ cell counts were similar between the 2 groups. Resistance testing results were available from 15 participants at Week 48; 8 in the QD group and 7 in the BID group. Genotypic analysis did not identify any LPV or TDF resistance mutations. Resistance to FTC was identified in a total of 3 participants (2 in the QD group and 1 in the BID group) [20].

A total of 19% of participants in the QD group and 25% in the BID group discontinued the study; 12% and 5% due to AEs, respectively. Gastrointestinal AEs were the most common cause for discontinuation. Overall, the most common AEs (>3%) reported were diarrhea, nausea, and vomiting, with diarrhea being reported more frequently in the QD group (16% vs. 5%; p=0.04). The most common Grade 3/4 laboratory abnormalities (>3%) reported were increased ALT (>5 x ULN), AST (>5 x ULN), triglyceride (>750 mg/dL), and amylase (>2 x ULN) levels; no significant differences between the two groups were observed [19].

Reference should be made to the most recent Truvada package insert for complete information relating to possible adverse reactions and toxicities.

Stavudine (d4T) (ZERIT®)

Stavudine (d4T) is an approved nucleoside analogue that has been evaluated extensively as monotherapy and in combination with other nucleoside analogues and protease inhibitors. Reductions in HIV-1 RNA and improvements in clinical status have been observed with monotherapy and combination therapy. The most common toxicity associated with d4T is peripheral neuropathy and, much less commonly, hepatic damage, pancreatitis, and lactic acidosis (which may have an increased risk when d4T is used with didanosine in pregnancy).

d4T has antiviral activity at doses of 0.5 mg/kg/day and higher [11, 12]. Marked declines in serum p24 antigen levels are observed with doses ≥ 1 mg/kg/day, further demonstrating the antiretroviral activity of d4T, but dosing at higher levels is limited by the development of drug-induced peripheral neuropathy [13, 14]. Improvements in the clinical parameters of body weight, Karnofsky performance score, and Spitzer Quality-of-Life score were observed at all doses, with a suggestion of greater and more prolonged clinical improvement with doses from 0.5-2.0 mg/kg/day. A phase II/III study of d4T versus ZDV demonstrated that d4T is well tolerated and has greater antiretroviral activity as compared with continued ZDV in subjects with > 6 months prior ZDV treatment and 50-500 CD4+ T lymphocytes/mm^3^ [15].

The licensed dose of d4T is 80 mg/day (40 mg 12 hours apart) in an immediate release (IR) capsule formulation.

d4T therapy has rarely been associated with motor weakness, occurring predominantly in the setting of lactic acidosis. If motor weakness develops, d4T should be discontinued.

Reference should be made to the most recent **Zerit** package insert for complete information relating to possible adverse reactions and toxicities.

Lamivudine (3TC) (EPIVIR®)

Lamivudine (3TC) is an approved, potent NRTI that is widely used for treating patients with HIV infection. Although 3TC is an effective NRTI, virus with a resistance mutation at codon 184 rapidly emerges within 2 weeks of monotherapy and is also seen with dual-nucleoside regimens.

3TC is an extremely well tolerated nucleoside analogue. Side effects include anemia, neutropenia, increase in liver function enzymes, peripheral neuropathy, rash, nausea, vomiting, diarrhea, headache, insomnia, malaise, fatigue, dizziness, and allergic reactions. Rare side effects include psychosis, mania, and thrombocytopenia.

3TC is phosphorylated to its active 5’-triphosphate metabolite (3TC-TP). 3TC-TP inhibits HIV-1 reverse transcriptase by terminating viral DNA chains and also directly inhibits the RNA- and DNA-dependent DNA polymerase activities of reverse transcriptase. In all acute virus-cell infections (including monocytes and fresh, human peripheral blood lymphocytes) tested with 3TC, anti-HIV-1 activity was demonstrated. In HIV-1-infected cells, 3TC in combination with ZDV has synergistic antiretroviral activity. Due to its potency and favorable safety profile, 3TC, in combination with ZDV and/or other agents, is a widely used component of antiretroviral regimens.

The currently recommended dose of 3TC is 150 mg BID. However, the PK profile of 3TC suggests that it may be successfully dosed on a once daily basis. The observed mean elimination half-life in HIV-infected subjects ranged from 5 to 7 hours. However, 3TC 5’-triphosphate has an intracellular half-life of 10.5 to 15.5 hours in HIV-infected and uninfected human peripheral blood lymphocytes. The intracellular anabolic pathway for 3TC has a saturable formation step from the diphosphate to the active triphosphate. This allows pooling of 3TC-DP, which is the immediate precursor of 3TC-TP. Thus, 3TC-TP can still be formed when plasma concentrations are low long after dosing. The active moiety, 3TC-TP, is slowly eliminated from cells with an intracellular half-life of 11 to 13 hours in HIV-infected cells in vitro.

These findings have been supported by clinical data. A PK study of 3TC phosphorylation in peripheral blood mononuclear cells (PBMCs) from HIV-infected subjects found the 3TC-TP intracellular half-life to be approximately 15-16 hours, irrespective of dose, either 150 mg BID or 300 mg BID [22]. Another study demonstrated the PK equivalence of 3TC 300 QD to 3TC 150 mg BID in healthy volunteers with respect to plasma AUC_24,ss_, C_ave,ss_, and intracellular AUC_24,ss_, C_max,ss_, and C_ave,ss_ [23]. Thus, the relatively long plasma and intracellular half-lives, pooling of the immediate precursor (3TC-DP) of the active moiety (3TC-TP), and the PK equivalence of both dosing schedules support the ongoing investigation of 3TC 300 mg QD as a component of a multiple drug-dosing regimen.

In addition to PK data, several clinical studies support the further evaluation of once-daily 3TC administration. Preliminary 24-week data from one clinical study suggest the combination of QD 3TC + nevirapine + ddI administered to both naïve and experienced patients provides potent immunological and antiviral effects and warrants further evaluation [24]. In ICC-604, an open-label, pilot study evaluating QD 3TC + adefovir + ddI + efavirenz in subjects naïve to all four drugs (N=11), 100% and 90% of subjects achieved <400 and <50 copies/mL plasma HIV-1 RNA at 24 weeks by intent-to-treat (ITT) analyses.

Two clinical studies have compared 3TC BID with 3TC QD. In EPV40001, 159 ARV-naïve adults were randomized to one of three 3TC+ZDV+abacavir regimens (all given BID, 3TC given QD, or ABC given QD). At 48 weeks, both 3TC and ABC given QD demonstrated virologic equivalence compared with the respective twice daily regimens. All regimens were safe and well tolerated [25]. COLA4005 compared switching to 3TC QD versus continuing 3TC BID in virologically suppressed adult subjects on a stable regimen of either 3TC+d4T+indinavir or 3TC+d4T+nelfinavir. At week 24, the proportion of subjects with HIV-1 RNA <50 copies/mL was similar in the subjects who switched to 3TC QD and in those who remained on 3TC BID (95% vs. 90%; p=0.679, ITT M=F) [26].

Based on in vitro and supportive clinical data, the once daily administration of 3TC should pose no safety or efficacy issues and will add to the compact nature of study regimens.

Exacerbations of hepatitis B virus (HBV)-related hepatitis have been detected in HIV-HBV coinfected subjects that discontinued 3TC-containing antiretroviral regimens. Most episodes were self-limited, however fatalities have been reported. Co-infected subjects should be monitored for exacerbation of their hepatitis if 3TC is permanently discontinued.

Reference should be made to the most recent **Epivir** package insert for complete information relating to possible adverse reactions and toxicities.

Emtricitabine (FTC, Emtriva™)

FTC is a synthetic nucleoside analogue with activity against HIV-1 reverse transcriptase. In controlled clinical studies (FTC-301A, FTC-303) the antiviral activity of FTC was superior to d4T and was equivalent to 3TC.

The most common AEs that occurred in patients receiving FTC with other ARVs in clinical trials were headache, diarrhea, nausea, and rash, which were generally of mild to moderate severity. Approximately 1% of patients discontinued participation in the clinical studies due to these events. All AEs were reported with similar frequency in FTC and control treatment groups with the exception of skin discoloration, which was reported with higher frequency in the FTC-treated group. Skin discoloration, manifested by hyperpigmentation on the palms and/or soles was generally mild and asymptomatic. The mechanism and clinical significance are unknown.

*Hepatitis B*

Exacerbations of HBV have been reported in patients after discontinuation of FTC. Patients, who are coinfected with HBV and HIV, may have increased values on liver function tests and exacerbation of hepatitis symptoms when FTC is stopped. Usually these symptoms are self-limiting; however, serious complications have been reported. The causal relationship to FTC discontinuation is unknown. Patients coinfected with HBV and HIV should be closely monitored with both clinical and laboratory follow-up for several months after stopping FTC treatment.

Additional information regarding FTC is available in the most recent Emtriva package insert.

### 2.2 **Rationale**

**Hypothesis: There are two counterpoised hypotheses that will be tested in this study. Given the lack of conclusive clinical data, we are truly in a position of equipoise.**

**1. The negative hypothesis: Initiating antiretroviral therapy during the course of treatment for an acute OI will lead to more adverse drug events, treatment interruptions, and complications to the course of the presenting OI. In turn, this will result in a higher combined rate of death and AIDS-defining OIs, and a higher rate of virologic failure in the “immediate” ARV therapy group at the end of 48 weeks.**

**2. The positive hypothesis: Initiating antiretroviral therapy during the course of treatment of an acute OI will lead to a more rapid recovery of immune function, improve response to OI treatment, and lead to a lower combined rate of death and AIDS-defining OIs, and a lower virologic failure rate at 48 weeks.**

A frequently asked question for patients presenting with an acute OI is: Should HAART regimens be initiated during the treatment of the OI? The rationale for initiating therapy includes the above-noted benefits in terms of immune reconstitution. If the OI is the result of a badly damaged immune system and if HAART can result in rapid improvements in immune function, would the early initiation of HAART improve clinical outcomes in this patient group? On the other hand, starting ARV therapy early in the course of treating an OI clearly complicates the management of these patients and may actually worsen clinical outcomes. Outcomes may be worsened by increased adverse drug-drug interactions and drug-related toxicities. Moreover, for OIs like *Pneumocystis carinii* pneumonia (PCP) and cytomegalovirus (CMV) disease**,** an inflammatory reaction occurs when treatment for those OIs is started, often contributing to the morbidity and mortality from these conditions. Initiation of HAART also has been associated with inflammatory reactions in patients harboring CMV and *Mycobacterium avium* complex (MAC) without signs of acute disease.

The Problem of Immune Reconstitution Inflammatory Syndrome (IRIS)

Almost from the beginning of PI-based ARV availability, an immune reconstitution inflammatory syndrome (IRIS) has been recognized in some patients who initiate ARV. This syndrome is the subject of a review [27] and has been reported in patients with CMV, MAC, tuberculosis, cryptococcal disease, varicella zoster virus (VZV), and PCP. It is not clear what the overall prevalence of this condition is, nor is it clear what the risk factors for this syndrome are. It appears to occur in some patients even after treatment for the underlying OI has been ongoing and the OI appears to be clinically controlled. The management of the syndrome is also largely undefined. How the timing of ARV vis-à-vis the treatment of the OI influences this syndrome is also undefined.

Currently there are limited data from clinical cohorts and no data from clinical trials that help to define the optimal timing for initiating ARV in patients presenting with acute OIs. In a case series report from France [28], investigators describe several cases of patients starting ARV during the acute treatment for PCP. In the three cases described, all patients experienced clinical deterioration with the initiation of ARV marked by increasing hypoxia and/or new pulmonary infiltrates. Unfortunately, the conclusions that can be drawn from these cases are limited by the fact that all of the cases had their corticosteroid therapy withdrawn at the time of initiating ARV, and therefore, it is not clear whether the clinical deterioration was related to ARV.

Other recent case reports have noted complications in the treatment of cryptococcal meningitis and other OIs, which appear to be related to an IRIS associated with early initiation of HAART [29].

Despite these case reports, there is a large body of evidence that HAART improves survival in a variety of clinical settings, including in patients presenting with new or recurrent PCP. In the Adult and Adolescent Spectrum of HIV Disease (ASD), investigators from the CDC have shown that being on concurrent HAART at the time of PCP was independently associated with improved short-term and 12-month mortality rates [30].

It should be pointed out that A5164 will be of important clinical value no matter whether evidence is found for the positive or negative hypothesis. Should the truth be ‘paradoxical’ wherein, for example, the death/AIDS-OI rate is highest in the arm with the lowest virologic failure rate, the chance that either the positive or negative hypotheses will be confirmed is low.

Rationale for Primary Endpoint

The primary endpoint will be a combined endpoint of survival, AIDS progression, and virologic control at week 48 (after study enrollment). The outcome variable is an ordinal variable such that being alive with incomplete virologic control is considered a partial treatment success. There are three possible outcomes: alive without AIDS progression and with viral load < 50 copies/mL (best result), alive without AIDS progression and with detectable viral load (intermediate result), and AIDS progression and/or death (worst result).

A 48-week endpoint was chosen because if the deferred group does not start ART for a protracted period of time (e.g., week 12), then incomplete virologic response in the short term could simply reflect early treatment response and not a failed response. Allowing a 48-week endpoint should allow differences in response related to different start times to “wash out” sufficiently such that any difference seen would be related to actual differences in efficacy of ART between the two groups. In other words, an earlier endpoint (e.g., 24 weeks) has the potential to bias the result in favor of early treatment. Furthermore, a 48-week endpoint is a standard time frame to judge antiretroviral therapy in naïve subjects, and this study parallels this framework.

Rationale for ART Efficacy Estimates

In a recent review of the virologic and immunologic efficacy of HAART regimens in naïve subjects, Bartlett et al. summarized the findings from 23 clinical trials comprising 31 independent treatment groups with 19 unique antiretroviral regimens [31]. The clinical trials reviewed included PI-based triple ARV regimens, NNRTI-based triple therapy, and triple nucleoside regimens. The median CD4+ cell count at entry to the trials reviewed was 375 and no subject had an active OI. The percent of subjects who achieved a plasma viral load of < 50 copies/mL at 48 weeks was 47% using an ITT analysis. The 95% confidence interval around this estimate was 43% to 51%. In a clinical trial of antiretroviral-naïve subjects using a LPV/r-based triple regimen similar to the regimen proposed for this study, the 48-week response was 75 to79% [32]. However, in these trials the patient populations were not acutely ill as compared with the subjects proposed for this study.

There are no clinical trials that have evaluated the efficacy of ART for patients who are experiencing an acute OI. In fact, having an active OI traditionally has been an explicit exclusion criterion for ARV clinical trials. We are therefore required to extrapolate estimates of response from clinical trials of ARV in patients with advanced disease but without an acute OI. In Merck 039, patients with advanced disease were randomized to receive a triple-drug regimen of zidovudine (ZDV), lamivudine (3TC), and indinavir (IDV) versus IDV versus ZDV/3TC. Patients were eligible for the study if their CD4+ cell counts averaged <50 cells/mL on two separate measurements prior to enrollment and subjects also had to have at least 6 months of prior ZDV therapy. In addition, they could have had other NRTI therapy but had to be PI naïve. The median CD4+ cell count at study entry was 15 (range 5 to 63) and 62% had a prior AIDS-defining diagnosis. Subjects had over 2 years of prior treatment with ZDV and 85% had other prior nucleoside therapy. The primary endpoint of the study was 24 weeks, and 45% of the subjects assigned to the triple-drug regimen achieved < 50 copies/mL at this time point using an ITT analysis (noncompleter equals failure). In a subgroup of subjects followed out to 86 weeks, 35% (CI 28-45%) maintained < 50 copies/mL using a strict NC=F ITT analysis, whereas, 59% (CI 46-71%) maintained this level of control at week 84 in the on-treatment analysis.

Using the above data we estimate that in an antiretroviral-naïve patient group with acute OI or pneumonia and CD4+ cell counts of < 200 cells will likely have a response at week 48 in the range of 40 to 60%. This is within the range of response rates seen in the above studies of treatment-naïve subjects or subjects with advanced disease. The factor that favors a better response than that seen in the Merck 039 study is the greater intrinsic potency of a boosted PI regimen over a single PI-based regimen. Furthermore, we believe the proposed regimen will be easier to adhere to compared with IDV as prescribed in the double blind 039 trial. Another factor that should result in a more favorable response in this study compared to the Merck study, is **that** the patients for this study will likely be ART-naïve or will have only minimal pretreatment, unlike subjects in the Merck study who were heavily pretreated with nucleoside inhibitors. These factors should favor a better response than that seen in Merck 039. On the other hand, the subjects for this study obviously will be more acutely ill and this may result in a less favorable response. Balancing these factors leads us to use the estimated range of virologic response (< 50 copies/mL) of 40 to 60% at 48 weeks.

We have powered this study to be able to distinguish a difference of 20% in the virologic response at week 48 with a 10% difference in rates of AIDS progression and or death between the two arms (both favoring the immediate group). [See section 9.0.] We feel that being able to detect a true difference in virologic response that is less than 20% between the two arms would be of questionable clinical relevance. We believe that if the true difference in virologic response was less than 20% at week 48, most practitioners would be inclined to delay the initiation of ART in the setting of an acute OI given the inherent difficulties and complexities of initiating ART immediately.

Rationale for ARV Regimen

Although a ritonavir-boosted PI regimen has the potential for drug-drug interactions and can be difficult for some patients to tolerate, we feel that the LPV/RTV-based regimen proposed for this study is justified for several reasons. First, boosted-PI regimens are considered to be highly potent. Patients presenting with an acute OI and advanced disease require highly potent regimens. Second, PI-based regimens have been much more thoroughly studied in the setting of advanced disease. In fact, it is the only class of ARV in which the positive impact on morbidity and mortality has been conclusively demonstrated for patients with advanced disease. Moreover, it is interesting to note that in the meta-analysis by Bartlett discussed above, a PI-based regimen was associated with a somewhat better CD4+ cell response compared with NNRTI or triple NRTI regimens. Finally, given the nature of the patient population, it is likely that treatment interruptions (unintended interruptions) will occur and so we want to use a regimen with a relatively high genetic barrier to resistance so as to preserve treatment options over the long term for these patients.

We feel that the proposed regimen has many potential advantages over other regimens and would therefore suggest subjects start on a LPV/RTV-based regimen, but we have designed the trial to allow practitioners to change regimens liberally if problems arise.

Reference should be made to the toxicity management section (Section 7.0) for discussion of toxicities associated with the study-provided drugs.

## 3.0 STUDY DESIGN

A5164 is a randomized, phase IV study of the strategy of immediate versus deferred ART in subjects with advanced immunodeficiency syndrome who are presenting with acute AIDS-related opportunistic infection (OIs) or serious bacterial infections (BIs) for which there are recognized effective antimicrobial therapies available. The OIs included are the protocol-specific subset of the 1999 CDC AIDS-defining conditions (MMWR, March 12, 1999) or treatable AIDS related OI, with chair or vice chair approval. (See Appendix I for criteria used to determine study eligibility).

Eligible subjects will have received no ART for 8 weeks prior to study entry, less than 31 days of any ARV in the prior 6 months, and no more than 1 HAART regimen on which they experienced treatment failure (see section 4.2 for details.)

Subjects will enter Step 1 and be randomized to receive ART immediately (“immediate” group, Arm A) OR to have ART deferred (“deferred” group, Arm B). Subjects in Arm B will enter Step 2 only when they are ready to initiate ART and only if ART is initiated between weeks 4 and 32 (days 28 and 224, respectively).

ART

The study-provided drugs that will be available for constructing an ARV regimen are:

- Lopinavir/ritonavir (LPV/RTV)
- Emtricitabine/tenofovir disoproxil fumarate (FTC/TDF)
- Stavudine (d4T)

Study-provided drugs will be offered to subjects on Arm A in Step 1 and to Arm B subjects when they enter Step 2. Although the choice of a third or fourth ARV drug (other than ddI or ZDV if d4T is included in the regimen) is at the discretion of the site investigator, **either** 3TC **or** FTC is strongly recommended as the third ARV drug in **an** LPV/RTV plus d4T regimen. Substitutions for any of the study-provided drugs and/or additions can be made on a case-by-case basis using FDA-approved drugs and at the discretion of the site investigator. However, only LPV/RTV**,** FTC/TDF, and d4T will be supplied by the study.

Subjects will be followed through week 48, regardless of whether they are on ART **or** when they initiate ART. The primary endpoint will be a combined endpoint of survival, AIDS progression, and virologic control at week 48. The success of the ART will also be evaluated by virologic and immunologic response at study weeks 12, 24, and**,** when available**,** at 24 weeks after initiation of ART.

The success of the OI/BI treatment will be evaluated by duration of hospitalization, complication rates, adverse events, and serious toxicities that occurred over the course of the treatment of the OI/BI.

A5164 will be reviewed at least once per year by the NIAID Data Safety and Monitoring Board (DSMB).

ART Initiation

For subjects randomized to Arm A, ART should be initiated within 48 hours after entry, and within 14 days after the start of OI/BI treatment.

Subjects randomized to Arm B will receive study-provided drug only after they enter Step 2. Subjects in Arm B will enter Step 2 only if they initiate ART between week 4 (day 28) and week 32 (day 224). Very strong consideration should be given to starting ART for Arm B subjects between week 6 (day 42) and week 12 (day 84). If Arm B subjects initiate ART outside of the **week 4–32** timeframe, they will not register to Step 2 and will not receive study-provided drugs, but will continue follow-up on Step 1 through week 48.

Screening Log

Because the A5164 protocol team is monitoring enrollment during the study, all sites will be asked to maintain a record of the reasons prospective subjects do not enroll in A5164. Sites will be asked to supply the team with monthly summaries of these data until the study closes to accrual. No individually identifiable information should be included in the monthly summaries. The team will review these data on a regular basis to identify barriers to enrollment and make appropriate changes to the protocol when necessary.

## 4.0 SELECTION AND ENROLLMENT OF SUBJECTS

### 4.1 Step 1 Inclusion Criteria

- - 1. HIV-1 infection, as documented by any licensed ELISA test kit, HIV-1 culture, HIV-1 antigen, or plasma HIV RNA. If not performed prior to study entry, confirmation of ELISA by Western blot, HIV culture, antigen detection, HIV RNA, or another ELISA test must occur within 10 days after entry.
    2. For subjects with bacterial pneumonia or serious bacterial infection, CD4+ cell count <200 cells/mm^3^, obtained within 30 days prior to study entry from a laboratory that possesses a CLIA certification or its equivalent **(for ACTUs)**, or **from** a DAIDS-approved laborator**y (for ICTUs)**.
    3. Laboratory values obtained within 7 days prior to study entry.
- Absolute neutrophil count (ANC) ≥ 500/mm^3^.

NOTE: Cell Stimulating Factor (CSF) therapy should be utilized as needed.

- Hemoglobin ≥ 7.0 g/dL.
- Platelet count ≥ 50,000/mm^3^.
- AST (SGOT), ALT (SGPT), and alkaline phosphatase ≤ 5 × ULN.
- Total bilirubin ≤ 2.5 × ULN.
- Creatinine clearance ≥ 26 mL/min as estimated by the Cockcroft-Gault equation [33].

For serum creatinine concentration in mg/dL:

For men, [(140 – age in years) × (body weight in kg)] ÷ (serum creatinine in mg/dL × 72).*

*For women, multiply the result by 0.85.

NOTE: Calculation program can be found on DMC website ([**https://www.fstrf.org/**](https://www.fstrf.org/)**ACTG/index.html**).

- - 1. All women of reproductive potential (have not reached menopause or undergone hysterectomy, bilateral oophorectomy, or tubal ligation) must have a negative serum or urine β-HCG pregnancy test with a sensitivity of at least 50 mIU/mL performed within 7 days before study entry.
    2. Female subjects who are not of reproductive potential (have reached menopause or undergone hysterectomy, bilateral oophorectomy, or tubal ligation) or whose male partner has undergone successful vasectomy with resultant azoospermia or has azoospermia for any other reason, are eligible without requiring the use of contraception. Documentation of menopause, sterilization (hysterectomy, oophorectomy, tubal ligation, or vasectomy) and azoospermia by patient-reported history is acceptable.

NOTE: Requirements for subjects anticipating taking efavirenz (EFV):

1) Female subjects without reproductive potential are eligible without the use of contraceptives if written or oral documentation communicated by clinician or clinician’s staff confirming lack of reproductive potential is made using one of the following: physician report/letter describing lack of reproductive potential; discharge summary documenting sterilization procedure; or, FSH measurement elevated into the menopausal range as established by the reporting laboratory.

2) If the female subject reports a history of infertility based on one of the above categories but documentation by clinician or clinician’s staff is not available, she must agree to use at least one barrier method of contraception, with a possible second method required at the discretion of the site investigator. Documentation of the subject’s statement regarding infertility must be entered in the medical record.

- - 1. All subjects must not participate in a conception process (i.e., active attempt to become pregnant or to impregnate, sperm donation, in vitro fertilization), and if participating in sexual activity that could lead to pregnancy, the female study volunteer/male partner must use a form of contracepti**ve** as specified in the note below while receiving protocol-specified medication(s) and for 4 weeks following discontinuation of protocol-specific medication(s).

Some study-provided or study-recommended drugs interact with hormonal-based methods. Therefore, hormonal-based methods alone are not sufficient. At least one of the following methods MUST be used appropriately (with or without a hormonal-based method) **while taking study-provided or –recommended drugs and** for at least 4 weeks after stopping study-provided or –recommended drugs unless documentation of menopause, sterilization, or azoospermia is present:

- Condoms (male or female) with or without a spermicidal agent
- Diaphragm or cervical cap with spermicide
- IUD

NOTE **A**: Subjects are not required to begin to use **a** contracepti**ve** until they have resumed sexual activity.

NOTE B: Requirement for subjects who anticipate taking EFV: Two of the above methods (with or without a hormonal-based method) MUST be used appropriately while taking EFV and for at least 4 weeks after stopping EFV unless documentation of menopause, sterilization, or azoospermia is present.

4.1.7 Confirmed or probable acute infections as defined in Appendix I. These definitions are to be used for eligibility criteria only.

The eligible infections include (confirmed or probable except when noted):

- *Pneumocystis carinii* pneumonia (PCP)
- Bacterial pneumonia (plus CD4+ cell count < 200 cells/mm^3^)
- Cryptococcal meningitis (confirmed only)
- Disseminated histoplasmosis
- Disseminated *Mycobacterium avium* complex (MAC)
- Cytomegalovirus (CMV) retinitis
- Cytomegalovirus (CMV) encephalitis
- Toxoplasmic encephalitis
- Other atypical mycobacterial infections (non-tuberculous, non-MAC)
- Other serious, invasive bacterial infections (bacterial infection of deep tissue, body cavity, or other normally sterile site) (plus CD4+ cell count < 200 cells/mm^3^)
- Other serious OIs, including other AIDS-defining and AIDS-related OIs, for which appropriate therapy other than ART exists, with permission of the protocol chair or vice chair.

NOTE: TB alone is not a qualifying OI.

4.1.8 Age ≥ 13 years.

4.1.9 Ability to take oral medications.

- - 1. Ability and willingness of subject or legal guardian/representative to give written informed consent. Local IRBs must specifically allow the use of a guardian's or representative's approval before such approval can be used to enroll subjects who are wards of the state.
    2. Current treatment for OI or bacterial infection listed in 4.1.7.

NOTE: Current treatment must have been started within 14 days prior to entry, but may have been discontinued prior to entry.

### 4.2 Step 1 Exclusion Criteria

- - 1. Receipt of any ART within the 8 weeks prior to study entry.
    2. Receipt of 31 or more days of any ARV within the 6 months prior to study entry.
    3. A history of more than one virologic, immunologic, or clinical treatment failure (i.e., progression of disease while on treatment) on a HAART regimen (see definition below) - or a history of more than one regimen change for unknown reasons.

NOTE A: suspicion of treatment failure on the part of the treating clinician is sufficient to establish history: documentation is not required.

NOTE B: Changes for documented reasons other than failure should not be included in the count of previous failures.

NOTE C: HAART regimen is defined as any of the following:

a. a PI with at least 2 NRTIs

OR

b. a boosted PI with or without NRTIs

OR

c. a NNRTI with at least 2 NRTIs

OR

d. a triple nucleoside regimen

OR

e. PI (or boosted PI) with an NNRTI

4.2.4 Pregnancy or breast-feeding.

4.2.5 Use of the following drugs within 30 days prior to study entry: systemic cancer chemotherapy, and immunomodulators (growth factors, immune globulin, interleukins, interferons [unless for HCV or Kaposi’s sarcoma]). Systemic investigational (not FDA-approved) agents will be allowed at the site investigator’s discretion if thought to be potentially lifesaving. Based on available information and the judgment of the site investigator, investigational agents that may be used should have a low likelihood of a major PK or toxicity interaction with the ARV drugs being given to the subject.

NOTE: Receipt of HIV vaccines or systemic corticosteroids is allowed.

4.2.6 Anticipated receipt of any of the prohibited medications listed in section 5.5.1 if it would be contraindicated for the ARV regimen constructed for the subject. Medications listed in section 5.5.2 also are prohibited if they have been used within 7 days before beginning enrollment to the study and if they would be contraindicated for the ARV regimen constructed for the subject.

4.2.7 Use of investigational antiretroviral agents at study entry.

4.2.8 Renal failure requiring dialysis.

4.2.9 Receipt of therapy for more than 14 days **f**or the first-treated and diagnosed OI/BI (i.e., enrolling diagnosis).

NOTE: If potential subject has more than one current diagnosis of an OI/BI, sites must send a message to the protocol chair and vice chair (providing treatment and diagnosis information) at [actg.teama5164@fstrf.org](mailto:actg.teama5164@fstrf.org) for determination of the qualifying OI/BI. The treatment information must include the medications and their start date for each diagnosis.

4.2.10 Active drug or alcohol use or dependence that, in the opinion of the investigator, would interfere with adherence to study requirements.

4.2.11 A known resistance profile or past history of ART responses that precludes administration of an effective ART of three or four FDA-approved drugs. An effective ART is defined as one that the site investigator expects will produce HIV-1 viral suppression.

4.2.12 The **enrolling diagnosis** is a recurrence of any OI listed in Appendix I that occurred within 90 days prior to study entry.

NOTE: Recurrent BIs are not exclusionary.

### 4.3 Step 2 Inclusion Criteria

4.3.1 Step 1 randomization to Arm B.

4.3.2 Step 2 entry must be on or after week 4 (day 28) and no later than week 32 (day 224, inclusive) after study entry.

4.3.3 Chemistry and hematology laboratory values listed in section 4.1.3 obtained within 7 days prior to entry in Step 2.

- - 1. For women of reproductive potential, a pregnancy test must be performed within 7 days before Step 2 registration.

NOTE A: Pregnancy is not exclusionary.

NOTE B: Site personnel are responsible for ensuring that an appropriate ART is prescribed, based on the results of the pregnancy test.

### 4.4 Step 2 Exclusion Criteria

- - 1. Anticipated receipt of any of the prohibited medications listed in section 5.5.1 if it would be contraindicated for the ARV regimen constructed for the subject. Medications listed in section 5.5.2 also are prohibited if they have been used within 7 days before Step 2 registration and if they would be contraindicated for the ARV regimen constructed for the subject.
    2. Initiation of any ART while on Step 1.

4.4.3 Breast-feeding.

### 4.5 Study Enrollment Procedures

4.5.1 Prior to implementation of this protocol, **ACTUs** must have the protocol and consent form approved by their local institutional review board (IRB). **For** ICTUs, all registration materials should be forwarded to [ICTUprotocol@s-3.com](mailto:ICTUprotocol@s-3.com) at the ACTG Operations Center for a preliminary review for completeness. The ACTG Operations center will forward the original registration packet to the DAIDS Regulatory Compliance Center (RCC). ACTUs and ICTUs must be registered with and approved by the DAIDS Regulatory Compliance Center Protocol Registration Office. Protocol registration must occur before any subjects can be enrolled in this study.

4.5.2 Once a candidate for study entry has been identified, details will be carefully discussed with the subject. The subject (or parent or legal guardian if the subject is younger than **the** age **of consent** or under guardianship) will be asked to read and sign the consent form that was approved by both the ACTU’s IRB and the DAIDS Regulatory Compliance Center. If the subject and legal guardian are unable to read, the process for consenting illiterate subjects, as defined by the local IRB, should be followed.

4.5.3 The Division of AIDS has concluded that this protocol does NOT meet Federal requirements governing prisoner participation in clinical trials and should NOT be considered by local IRBs for the recruitment of prisoners.

4.5.4 Subjects who meet eligibility criteria for A5164 will be registered to the study according to standard ACTG Data Management Center procedures.

- - 1. Subjects on the deferred therapy arm (Arm B) who meet the entry criteria (sections 4.3 and 4.4) and are ready to initiate ART will be registered to Step 2 through SDAC/DMC and receive a new SID number. The pharmacist must receive a prescription with the Step 2 SID number in order to dispense Step 2 therapy.

4.5.6 All sites will be asked to maintain a record of the reasons prospective subjects do not enroll in the study. Sites will be asked to supply the team with monthly summaries of these data until the study closes to accrual. No individually identifiable information may be included in these monthly summaries.

### 4.6 Coenrollment Guidelines

Coenrollment into A5128 is strongly encouraged. Coenrollment into any other study must be approved by the A5164 chair/vice chair.

## 5.0 STUDY TREATMENT

### 5.1 Regimens, Administration, and Duration

5.1.1 Regimen

All subjects will enter Step 1 and be randomized to Arm A or Arm B.

Step 1: Arm A

Subjects randomized to Arm A in Step 1 should initiate ARV therapy within 48 hours after entry, and within 14 days after the start of treatment for the OI/BI, and will remain on ARV therapy through week 48. Subjects in Arm A will not enter Step 2.

In this study there will be administration of an effective FDA-approved three-drug or four-drug ARV regimen. The following are study-provided drugs that will be available for constructing an ARV regimen:

- Lopinavir/ritonavir (LPV/RTV)
- Emtricitabine/tenofovir disoproxil fumarate (FTC/TDF)
- Stavudine (d4T)

NOTE: All of the drugs listed above will be available for all subjects who enroll under Version 3.0. Subjects who enrolled prior to Version 3.0 (i.e., before FTC/TDF was available) may elect to receive FTC/TDF through A5164 at the time they consent to Version 3.0. A switch to FTC/TDF will not be considered a failure of the previous treatment regimen.

See section 5.2.1 for d4T dose adjustments based on creatinine clearance values.

LPV/RTV, FTC/TDF, and d4T will be offered as study-provided drugs. The choice of a third or fourth ARV drug (other than ddI or ZDV if used in combination with d4T) is at the discretion of the site investigator. **Either** 3TC **or** emtricitabine (FTC) is strongly recommended for use as the third drug in the LPV/RTV plus d4T regimen. For additional flexibility, and only as an option, TDF can be used as an alternative for either 3TC or d4T, and ZDV as an alternative for d4T, but neither 3TC, FTC, TDF, nor ZDV will be supplied by the study.

NOTE: Subjects <18 years of age cannot receive FTC/TDF in this study.

Step 1: Arm B

Subjects randomized to Arm B in Step 1 will not receive any study-provided drug until enrolled in Step 2.

Step 2: Arm B Subjects Only

Subjects in Arm A will not enter Step 2. Subjects randomized to Arm B will receive study-provided drug only after they enter Step 2. Subjects in Arm B will enter Step 2 only if they initiate ART between week 4 (day 28) and week 32 (day 224). Very strong consideration should be given to starting ART between week 6 (day 42) and week 12 (day 84). Although the site investigator may begin ART at any time believed to be in the best interest of the subject, if ART is initiated outside of the **week 4-32** timeframe, subjects in Arm B will not register to Step 2 and will not receive study-provided drugs, but will continue follow-up on Step 1 through week 48.

Subjects in Arm B must be re-registered to **enter** Step 2 according to the ACTG Data Management standard procedures, and a prescription with the new SID number must be written for the pharmacist to dispense study-provided drugs in Step 2. This registration should be done only **when** ART is expected to start within 48 hours.

The following are study-provided drugs that will be available for constructing an ARV regimen:

- Lopinavir/ritonavir (LPV/RTV)
- Emtricitabine/tenofovir disoproxil fumarate (FTC/TDF)
- Stavudine (d4T)

NOTE: All of the drugs listed above will be available for all subjects who enroll under Version 3.0. Subjects who enrolled prior to Version 3.0 (i.e., before FTC/TDF was available) may elect to receive FTC/TDF through A5164 either at the time they consent to Version 3.0 (if they are already receiving ART) or after consenting to Version 3.0 and at the time that they initiate ART. A switch to FTC/TDF will not be considered a failure of the previous treatment regimen.

See section 5.2.1 for d4T dose adjustments based on creatinine clearance values.

LPV/RTV**,** **FTC/TDF, and** d4T will be offered as study-provided drugs. The choice of a third or fourth ARV drug (other than ddI or ZDV if used in combination with d4T) is at the discretion of the site investigator. **Either** 3TC **or FTC is** strongly recommended for use as the third drug **in** **the LPV/RTV plus d4T** **regimen**. For additional flexibility, and only as an option, tenofovir can be used as an alternative for either 3TC or d4T, and ZDV as an alternative for d4T**, but 3TC, FTC, TDF, and ZDV will not be supplied by the study**.

**NOTE: Subjects <18 years of age cannot receive FTC/TDF in this study.**

- - 1. Administration

The following formulations of solid dosages and oral solutions are available for administration:

- LPV/RTV: 400 mg/100 mg will be administered as three 133 mg/33 mg capsules or as 5mL (LPV 80 mg/RTV 20 mg per mL) of oral solution, orally BID with food. The latest package insert for LPV/RTV should be consulted if LPV/RTV is to be used in combination with efavirenz, nevirapine, amprenavir or nelfinavir.
- FTC/TDF: one tablet (200 mg of FTC and 300 mg of TDF in each tablet) will be administered orally once daily with or without food.
- d4T**:** 40 mg will be administered orally BID for subjects weighing ≥ 60 kg or as 30 mg orally BID for those weighing < 60 kg, with or without food.

See section 5.2.1 for dose adjustments based on creatinine clearance values.

5.1.3 Duration

Subjects enrolled in Arm A will receive study-provided drugs for a maximum of 48 weeks. Subjects enrolled in Arm B may receive study-provided drugs from the time they enter Step 2 through study week 48. Therefore, subjects in Arm B will not receive study-provided drugs for a full 48 weeks (see section 6.1, Schedule of Events, for further clarification).

### 5.2 Dose Adjustments: Step 1, Arm A and Step 2 Arm B

Dosage adjustment for subjects with organ dysfunction will be at the discretion of the **site** investigator. Any other dosage adjustment will be left to the site investigator on the basis of current recommended practices (e.g., adjustments for body weight).

NOTE: Before making any dosage adjustments, the latest package insert should be consulted. To avoid drug interaction adverse events, package inserts of concomitant agents should be referred to whenever a concomitant medication is initiated or dose changed.

- - 1. Dose Adjustments

Stavudine and lamivudine, if used, will be adjusted for creatinine clearance (calculated) **as follows**:

Stavudine (d4T)

- **c**reatinine clearance 26 – 50 mL/min:

d4T 20 mg po BID for subjects ≥ 60 kg

**OR**

d4T 15 mg po BID for subjects < 60 kg.

Lamivudine (3TC)

- **c**reatinine clearance 30 – 49 mL/min**:**

3TC 150 mg po QD

- **c**reatinine clearance **15** – 29 mL/min**:**

3TC 150 mg on the first day, then 100 mg po QD thereafter.

Changes in ART will be allowed at the discretion of the site investigator.

5.2.2 Regimen Substitutions

Additional drugs and substitutions with FDA-approved drugs can be made in the ARV regimens for any of the study-provided drugs based on the judgment of the site investigator on a case-by-case basis. However, only LPV/RTV**,** FTC/TDF, and d4T will be provided by the study. Other ARV drugs such as TDF for either 3TC or d4T, or ZDV for d4T may be used at the discretion of the site investigator. If any of the study-provided drugs (LPV/RTV, FTC/TDF, or d4T**)** are not used in the initial regimen but are subsequently chosen for use as substitute drugs, they will be provided by the study.

5.2.3 ARV Salvage Regimens

If the treating physician does not consider the response to the initial ARV regimen adequate, ARV salvage regimens will be allowed on the basis of a genotype test. Real-time genotyping will be offered no earlier than 8 weeks after initiating ARV, and salvage therapy will be allowed after 10 weeks of the initial ARV regimen. Virologic failure that would trigger a genotype test and evaluation for salvage therapy is defined in section 6.3.13. Drug substitutions will be at the discretion of the site investigator.

### 5.3 Product Formulation and Preparation

5.3.1 Lopinavir/ritonavir (LPV/RTV, LPV/r; Kaletra): is coformulated to contain 133.3 mg of lopinavir and 33.3 mg of ritonavir in a soft-gelatin capsule and as 80 mg of lopinavir/20 mg of ritonavir per mL of oral solution. Store at 2° to 8°C (36° to 46°F) prior to dispensing. Avoid exposure to excessive heat. LPV/RTV should be kept refrigerated after it is dispensed to subjects. If LPV/RTV is stored at room temperature up to 25°C (77°F), the capsules or oral solution should be used within 2 months.

5.3.2 Emtricitabine 200 mg/tenofovir disoproxil fumerate 300 mg fixed-dose combination tablet (FTC/TDF, Truvada) is co-formulated to contain FTC 200 mg and TDF 300 mg in each tablet. Each bottle contains a silica gel desiccant canister that should remain in the original container to protect the product from humidity. Dispense in the original container. Store at 25°C (77°F); excursions permitted to 15° to 30°C (59° to 86°F).

5.3.3 Stavudine (d4T, Zerit) will be available in two dosage forms: 40 mg, 30 mg, 20 mg, or 15 mg capsules and a powder blend yielding a 1 mg/mL strength following reconstitution. Prior to reconstitution, stavudine powder should be stored at room temperature 15° to 30°C (59° to 86°F). Stavudine capsules should be stored at room temperature 15°to 30°C (59°to 86°F).

Reconstitute the powder blend with 202mL of Purified Water, USP to obtain a concentration of 1 mg/mL of stavudine. Dispense solution in the original container. After reconstitution, oral solution should be stored in refrigerator at 2° to 8°C (36° to 46°F). The reconstituted solution is stable for 30 days. Shake the container vigorously prior to measuring each dose. Discard any unused portion after 30 days.

### 5.4 Product Supply, Distribution, and Pharmacy

5.4.1 Study Product Acquisition

Lopinavir/ritonavir will be provided by Abbott Laboratories.

Emtrictabine/tenofovir DF will be provided by Gilead Sciences**.**

Stavudine will be provided by Bristol-Myers Squibb.

Lopinavir/ritonavir, **emtricitabine/tenofovir DF, and** stavudine will be available through the NIAID Clinical Research Products Management Center. The ACTU **or ICTU** pharmacist **may** obtain the study-provided drugs for this protocol by following the instructions in the manual, *Pharmacy Guidelines and Instructions for DAIDS Clinical Trials Networks,* in the section Study Product Control.

5.4.2 Study Product Accountability

The ACTU **or ICTU** pharmacist is required to maintain complete records of all study products received from the NIAID Clinical Research Products Management Center and subsequently dispensed. All unused study products must be returned to the NIAID Clinical Research Products Management Center after the study is completed or terminated. The procedures to be followed are provided in the manual, *Pharmacy Guidelines and Instructions for DAIDS Clinical Trials Networks,* in the section Study Product Control.

### 5.5 Concomitant Medications

Given the wide variety and large number of drugs that could potentially be used in these critically ill subjects, the latest package insert or Investigators Brochure of all antiretroviral medications to be taken by the subject should be consulted to determine whether there are potential drug-drug interactions.

5.5.1 Prohibited Drugs

The prohibited medications listed below apply to the study-provided drugs (LPV/RTV, **FTC/TDF, and** d4T) and the study-recommended drugs (3TC **and FTC**). Other medications may be contraindicated or prohibited based on the most recent package inserts for the other ARV drugs included in a subject’s regimen.

- Flecainide (Tambocor), propafenone (Rhythmol), bepridil (Vascor), cisapride (Propulsid), ergot derivatives, systemic ketoconazole (Nizoral), lovastatin (Mevacor), simvastatin (Zocor), pimozide (Orap), phenytoin (Dilantin), phenobarbital (Luminol), carbamazepine (Tegretol), rifampin (Rifadin), rifapentine (Priftin), triazolam (Halcion), St. John’s wort (Hypericum perforatum), terfenadine and astemizole (Hismanal)
- Itraconazole at dose > 200 mg/day. Itraconazole plasma levels should be monitored if clinically indicated for doses < 200 mg/day.
- Investigational ARVs
- Due to potential drug interactions, subjects should not receive any of the following agents within 7 days before beginning ART: phenobarbital (Luminal), phenytoin (Dilantin), carbamazepine (Tegretol), rifampin (Rifadin), rifapentine (Priftin), or St. John’s wort (Hypericum perforatum).

5.5.2 Precautionary Medications

The precautionary medications listed below apply to the study-provided drugs (LPV/RTV, **FTC/TDF, and** d4T) or the study-recommended drug**s** (3TC **and FTC**). Other medications may be contraindicated based on the most recent package inserts for the other ARV drugs included in a subject’s regimen.

- Non-ARV investigational (not FDA-approved) agents may be allowed at the site investigator’s discretion if thought to be potentially lifesaving. **Sites should n**otify the team within 72 hours after initiation of the agent. Based on available information and the judgment of the site investigator, investigational agents that may be used should have a low likelihood of a major PK or toxicity interaction with the ARV drugs being given to the subjects.
- Rifabutin should be dose reduced 75% (**to** one quarter) of the recommended dose of 300 mg QD when given with LPV/RTV. A maximum dose of 150 mg QOD or three times per week is recommended.
- LPV/RTV results in decreased plasma concentration of ethinyl estradiol (Ortho Novum®), and alternative or additional contraception measures should be used (see section 4.1.6) when using these drugs.
- The LPV/RTV formulation contains ethanol which may cause a reaction with co-administration of disulfiram and metronidazole.

For subjects on either arm while receiving any ART:

Medications requiring careful monitoring and/or dose adjustments include: rifabutin, atorvastatin (Lipitor), sildenafil (Viagra, Silagra, or other generic preparations) or vardenafil (Levitra), tadalafil (Cialis), quinidine (Cardioquin), systemic lidocaine, theophylline, warfarin (coumadin), and dihydropyridine calcium channel blockers. Midazolam (Versed) may be used only with appropriate monitoring of respiration and availability of ventilatory support. Amiodarone (Cordarone) will be allowed in emergency situations provided that there is no other alternative. After 24 hours of repeated dosing or continuous use of quinidine, amiodarone, or systemic lidocaine, study drugs must be held unless drug levels for the precautionary medication are monitored.

- The use of clarithromycin (Biaxin) is discouraged; azithromycin (Zithromax) should be used as an alternative.
- Corticosteroids: subjects who are started on corticosteroids for PCP should complete a full 21-day course.

For other OIs, if a subject experiences paradoxical worsening of disease associated with acute immune inflammatory syndrome, corticosteroid treatment should be considered **if** clinically indicated.

### 5.6 Adherence Assessment

The adherence assessment used in this study will be the ACTG Adherence Questionnaire at the noted visits. Adherence to all ARVs will be assessed using subject self-report.

## 6.0 CLINICAL AND LABORATORY EVALUATIONS

### 6.1 Schedule of Events

STEP 1 ARM A (IMMEDIATE TREATMENT) AND ARM B (DEFERRED TREATMENT) UNTIL STEP 2

| Evaluation | Screening | Pre**-**  Entry | Step 1  Entry | Wk 4 | Wk 8 | Wk 12 | | Wk 16 | | Wk 24 | Wk 32 | Wk 40 | Wk 48 | Time of Suspected IRIS | Premature Study  D/C | ARV Treatment Discontinuation |
| --- | --- | --- | --- | --- | --- | --- | --- | --- | --- | --- | --- | --- | --- | --- | --- | --- |
| Documentation of HIV | X |  |  |  |  |  | |  | |  |  |  |  |  |  |  |
| Medical History | X |  |  |  |  |  | |  | |  |  |  |  |  |  |  |
| Medication History and Updates | X |  | X | X | X | X | | X | | X | X | X | X | X | X | X |
| Nadir CD4 |  |  | X |  |  |  | |  | |  |  |  |  |  |  |  |
| Clinical Assessments | X |  | X | X | X | X | | X | | X | X | X | X | X | X | X |
| Hematology | X |  | X | X | X | X | | X | | X | X | X | X |  | X | X |
| Chemistry^1^ | X |  | X^1^ | X | X | X | | X | | X | X | X | X |  | X | X |
| Liver Function Tests | X |  | X | X | X | X | | X | | X | X | X | X |  | X | X |
| Non fasting lipids (triglycerides)^2^ |  |  | X |  |  | X | |  | | X |  |  | X |  | X | X |
| Pregnancy Testing | X |  | Any time pregnancy is suspected | | | | | | | | | | |  |  |  |
| CD4+/CD8+ | X |  | X | X | X | | X | | X | X | X | X | X | X | X | X |
| HIV-1 RNA (real time) |  | X**^4^** | X^3^ | X | X | | X | | X | X | X | X | X | X | X | X |
| Genotyping (real time **or** stored) **See section 6.3.13** |  |  | X^3^ | At time of confirmed virologic failure, or at site investigator’s request | | | | | | | | | |  |  |  |
| Stored Plasma/PBMC |  |  | X | X |  | | X | |  | X |  |  | X | X | X | X |
| Multi-Dimensional Health Status Questionnaire |  |  | X |  | X | |  | | X |  | X |  | X |  | X | X |
| Adherence/Resource Instrument Self Report |  |  | X |  | X | |  | | X |  | X |  | X |  | X | X |
| Subject Contact for Follow-up**^5^** |  |  | Between 2-4 days following study entry and then again between 14-21 days  following study entry and at weeks 20, 28, 36, 44 | | | | | | | | | | | | | |

^1^ At study entry (and entry into Step 2) only, perform the following additional tests: calcium, phosphate, lactate dehydrogenase (LDH), total protein, albumin, and calculated creatinine clearance.

^2^ If the triglyceride result is > 1000 mg/dL, a fasted evaluation is required within 2 weeks.

^3^ An aliquot of the entry sample should be stored at the site for possible genotypic testing later in the study.

**^4^ HIV-1 RNA value obtained within 28 days and at least 24 hours prior to Step 1 entry may be used as the pre-entry value.**

**^5^** See Appendix **II**. These data should be completed as described but will not be entered on the CRFs.

STEP 2 – ARM B SUBJECTS INITIATING AR**T** BETWEEN WEEK 4 AND WEEK 32

| Evaluation | Step 2 Screening  (-7 days) | Step 2  Entry | +4 wk | +8 wk | +12 wk | +16 wk | +24 wk^2^ | +32 wk^2^ | +40 wk^2^ | Time of Suspected IRIS | Premature  Study  D/C | Final Study  Visit^2^ | ARV Treatment D/C |
| --- | --- | --- | --- | --- | --- | --- | --- | --- | --- | --- | --- | --- | --- |
| Medication Updates | X | X | X | X | X | X | X | X | X | X | X | X | X |
| Clinical Assessments |  | X | X | X | X | X | X | X | X | X | X | X | X |
| Hematology | X | X^1^ | X | X | X | X | X | X | X |  | X | X | X |
| Chemistry | X | X^1, 3^ | X | X | X | X | X | X | X |  | X | X | X |
| Liver Function Tests | X | X^1^ | X | X | X | X | X | X | X |  | X | X | X |
| Nonfasting lipids (Triglycerides)^5^ |  | X |  |  | X |  | X |  |  |  | X | X | X |
| Pregnancy Testing | X | **Any time p**regnancy is suspected | | | | | | | | |  |  |  |
| CD4+/CD8+ |  | X | X | X | X | X | X | X | X | X | X | X | X |
| HIV-1 RNA (real time) | X^4^ | X | X | X | X | X | X | X | X | X | X | X | X |
| Genotyping (real time **or** stored) **See section 6.3.13** |  | At time of confirmed virologic failure, or at site investigator’s request | | | | | | | | |  |  |  |
| Stored Plasma/PBMC |  | X | X |  | X |  | X |  |  | X | X | X | X |
| Multi-Dimensional Health Status Questionnaire |  | X |  | X |  | X |  | X |  |  | X | X | X |
| Adherence/Resource Instrument Self Report |  | X |  | X |  | X |  | X |  |  | X | X | X |
| Subject Contact for Follow-up^6^ |  | Between 2-4 days following Step 2 entry and then again between 14-21 days  following Step 2 entry and at weeks +20, +28, and +36 | | | | | | | | | | | |

^1^ Laboratory values obtained within 7 days prior to entry into Step 2 can be used as the **Step 2** entry values.

^2^ If needed to complete 48 weeks on study. A final study visit is required 48 weeks after study entry following a study visit occurring between 41 and 45 weeks after study entry. The final visit may be at the regular scheduled visit if it occurs between 46 and 47 weeks after entry.

^3^ At entry into Step 2 only, perform the following additional tests: calcium, phosphate, lactate dehydrogenase (LDH), total protein, albumin and calculated creatinine clearance.

^4^ HIV-1 RNA values obtained within 4 weeks (28 days) before to entry into Step 2 can be used as the screening sample.

^5^ If the triglyceride result is > 1000 mg/dL, a fasted evaluation is required within 2 weeks.

^6^ See Appendix **II**. These data should be completed as described but will not be entered on the CRFs.

### 6.2 Timing of Evaluations

6.2.1 Prerandomization/Preregistration Evaluations

**These evaluations o**ccur prior to the subject’s taking any ART, treatments, or interventions.

Screening

Screening evaluations to determine eligibility must be completed within 7 days prior to study entry unless otherwise specified **(see sections 6.3.11 and 6.3.12)**. Screening and pre-entry evaluations may occur concurrently.

Because the team is monitoring enrollment during the study, all sites will be asked to maintain a record of the reasons prospective subjects do not enroll in A5164. Sites will be asked to supply the team with monthly summaries of these data until the study closes to accrual. No individually identifiable information should be included in the monthly summaries. The team will review these data on a regular basis to identify barriers to enrollment and make appropriate changes to the protocol when necessary.

Pre-Entry

Evaluation must be completed **within 28 days but** at least 24 hours prior to entry evaluations. If a subject meets entry criteria based on the screening **evaluations**, then the subject is randomized and entry evaluations take place.

6.2.2 On-Study Evaluations

Evaluations should occur after randomization. All regularly collected data should be obtained regardless of whether the subject starts ART, unless the subject was subsequently determined not to have HIV infection. Study-specific guidelines for case report form (CRF) completion are provided on the A5164 protocol-specific web page (PSWP) (<http://aactg.s-3.com/members/ ps/5164/ps5164.htm>) and should be consulted if this occurs. Subjects will be followed using the study’s schedule as closely as possible. If **a** subject refuses follow-up through week 48**,** the Premature Discontinuation evaluations should be done rather than those listed for the regularly scheduled visit.

Step 1 Entry

Evaluations must occur at least 24 hours after pre-entry evaluation. Subjects should begin ART as defined in the protocol based on randomized treatment assignment.

After study entry into Step 1 (Arm A)

If a subject clinically deteriorates after the laboratory eligibility evaluations for safety (hematology, chemistries, and LFTs) have been obtained but before AR**T** has been started, investigators are requested to confirm that the safety laboratory evaluations still meet the study eligibility criteria prior to starting ART. Where clinically indicated, additional safety laboratory evaluations should be obtained before initiation of ART. If these laboratory values do not meet study eligibility criteria, initiation of ART must be delayed until the values improve to within the levels allowed for study eligibility.

Step 2 Entry (Arm B)

Eligibility evaluations may be performed up to 7 days prior to Step 2 entry**, except as noted**. The laboratory values obtained for eligibility may also be used as Step 2 entry visit **values**, however, sites must note that additional blood chemistry values are required at entry to Step 2.

Additional monitoring will occur according to the Step 2 **schedule**. Step 2 evaluations will occur every 4 weeks for the first 16 weeks after initiating ART and then every 8 weeks through week 48. **It is possible that the interval between the final two visits will be less than 8 weeks to ensure that n**o subject will exceed a total of 48 weeks on study.

6.2.3 At Time of Suspected IRIS Diagnosis

In the event that a suspected diagnosis of IRIS is made between scheduled study visits, the subject should be asked to come to the clinic as soon as possible so that a CD4+/CD8+ cell count and a plasma HIV-I RNA level can be determined and so that a plasma sample can be collected.

6.2.4 Premature Study Discontinuation

Subjects who prematurely discontinue the study should be asked to come to the clinic for the evaluations listed under Premature Study Discontinuation. If the subject’s previous visit occurred within 2 weeks prior to the discontinuation visit, only the evaluations not performed at that previous visit are required.

6.2.5 At Time of ART Discontinuation

Subjects who discontinue all ART should be followed, when possible, off-treatment/on-study for the remainder of the 48 weeks of study duration. Subjects should be asked to come to the clinic for the evaluations listed under Treatment Discontinuation for each arm. If the subject’s previous visit occurred within 2 weeks prior to the treatment discontinuation visit, only the evaluations not performed at that previous visit are required.

6.2.6 Pregnancy

Women who become pregnant may be allowed to remain on study and may remain on the study regimen if approved by the site investigator and if a specific pregnancy consent is obtained. Investigators may initiate or modify the regimen if necessary to be optimal for the subject.

### 6.3 Special Instructions and Definitions of Evaluations

Study-specific guidelines for CRF completion are provided on the A5164 protocol-specific web page (PSWP) (<http://aactg.s-3.com/members/ps/5164/ps5164.htm>).

6.3.1 Documentation of HIV

Includes antigen, antibody, or other recognized methods of documenting HIV infection from subject’s source document.

Subjects who enter the study but whose HIV-1 status is not confirmed will go off study and have no further evaluations performed. Instructions for CRF completion for these subjects are provided on the A5164 PSWP (<http://aactg.s-3.com/members/ps/5164/ps5164.htm>).

6.3.2 Medical History

A baseline medical history must be present in source documents. These data should also be recorded in the CRFs. The medical history should include any previous HIV-related diagnoses and non-HIV-related diagnoses of major organ systems.

Any allergies to any medications and their formulations must be documented, but do not need to be recorded on CRFs.

6.3.3 Medication History and Updates

Medication History

A medication history must be present in source documents and recorded in appropriate CRFs and it must include:

- Complete HIV treatment history, including start and stop dates of any antiretroviral medication (estimated if the exact dates cannot be obtained), immune-based therapy, or HIV-related vaccines, including blinded study medications.
- Complete treatment history of any prescription medications taken for the treatment or prophylaxis of OIs, including actual or estimated start and stop dates.
- All prescription medications (in addition to those noted above) taken within 30 days prior to study entry, including actual or estimated start and stop dates.
- Non-prescription medications taken within 30 days prior to study entry, including actual or estimated start and stop dates.

Guidelines for completing CRFs for HIV, OI, and BI treatments as well as for prophylaxis history are provided on the A5164 PSWP (<http://aactg.s-3.com/ members/ps/5164/ps5164.htm>).

Concomitant Medications

After entry, all prescription drugs started or stopped since the last visit will be recorded in the source document and will be recorded on the appropriate CRFs at each visit. source document must include dose changes for any OI/BI treatment as well as start dates and stop dates for all prescription drugs (including those taken as OI/BI treatment).

NOTE: Prophylactic medications taken for OI/BI must be recorded as concomitant medications on the CRFs.

Treatment Modifications

All modifications to ART **or to** treatment related to the OI/BI and to any recurrent OI or BI, including initial doses, subject-initiated and/or protocol-mandated interruptions for greater than 48 hours, and reasons for the modifications, and permanent discontinuation of treatment will be recorded on the CRFs at each visit. Subject-initiated and protocol-mandated interruptions include both inadvertent and deliberate interruptions of AR**V** dose(s).

NOTE: Medications taken for acute OI/BI must be recorded as treatment modifications on the CRFs.

6.3.4 Nadir CD4+

The subject’s prior nadir CD4+ cell count (absolute value and date) should be documented when possible with a copy of the nadir CD4+ cell count report. If this documentation is not available, then subject recollection will suffice. For subjects who do not know the exact nadir value and for whom there is no source documentation, then recall of the categorical nadir (e.g., <50, <100, <200 cells/mm^3^) will suffice.

6.3.5 Clinical Assessments

Targeted Physical Exam

A targeted physical examination will be completed within 30 days prior to study entry, at entry, and at each study visit during the study. The examination should be driven by any signs or symptoms previously identified that the subjects has experienced within 30 days prior to study entry or since the last visit. If symptoms of CMV retinitis are observed, a rapid ophthalmologic assessment should be conducted.

Weight

**W**eight must be obtained for all subjects at entry and at weeks 24 and 48 (or last visit). Self-reported data is acceptable if weight cannot be measured at study entry. For subjects in Arm B, weight must also be obtained at Step 2 entry, and at Step 2 week +24. All weights must be record**ed** on CRFs.

Signs and Symptoms

At study entry and at entry to Step 2, all signs and symptoms regardless of grade that took place within 14 days prior to entry should be recorded. After entry and for post-baseline or Step 2 assessments, sign/symptoms should be recorded on the CRFs if one or more of the following apply:

- all Grade 3 or higher signs/symptoms;
- any toxicity that leads to a change in ARV, OI/BI treatment, or treatment of recurrent opportunistic or bacterial infections, regardless of the grade;
- any sign/symptom that results in the reporting of an SAE.

Sites must refer to the Division of AIDS Table for Grading Severity of Adult Adverse Experiences, August 1992, which can be found on the Regulatory Compliance Center (RCC) Web Site: <http://rcc.tech-res-intl.com/members /aactg.htm>.

Diagnoses

All probable, confirmed, recurrent, or new diagnoses and any diagnoses that result in a modification of AR**T**, OI/BI treatment, or treatment of recurrent OI or BI, or require reporting on SAEs made since the last visit will be recorded in the source documents and on the CRFs.

Though not usually required, this study requires that the following information about diagnoses be recorded as well:

- Record any dates of resolution for all OI/BIs as well as the dates of OI/BI relapses and recurrences.
- Record any ongoing progression of OI/BI despite therapy for the disease.
- Record any potential diagnosis of an immune reconstitution inflammatory syndrome (IRIS) event.

Vital Signs

Temperature, pulse, and blood pressure **must be** recorded at all visits in the source documents*.*

Hospitalization

Hospitalization information **must** be collected beginning with the onset of the hospitalization for the OI/BI. After study entry**,** information about admission and discharge for all hospitalizations must be recorded in the source documents and on the CRFs.

LABORATORY EVALUATIONS

Any laboratory toxicities that led to a change in AR**T** or OI/BI treatment, regardless of grade, must be recorded on the CRFs. Screening values should be recorded in source documents. At study entry, all laboratory values must be recorded on the CRFs. At entry to Step 2, all laboratory values must be recorded on the CRFs, including values obtained up to 7 days earlier that are being used as both screening and entry values. Hematology, blood chemistries, and liver function tests performed as part of the subject’s hospital care may be used as the entry visit values if they occur on the day of entry. For all other on-study assessments, all Grade ≥ 3 laboratory toxicities should be recorded on the CRFs.

Sites should refer to the Division of AIDS Table for Grading **Severity of** Adult Adverse Experiences**,** August 1992, which can be found on the Regulatory Compliance Center (RCC) Web Site: [http://rcc.tech-res-intl.com/members/ aactg.htm](http://rcc.tech-res-intl.com/members/aactg.htm).

6.3.6 Hematology

Hemoglobin, hematocrit, white blood cell count (WBC), differential WBC, absolute neutrophil count (ANC), and platelets.

6.3.7 Blood Chemistries

Determinations of serum lipase, glucose, creatine kinase, sodium, potassium, chloride, bicarbonate, and creatinine are to be made at every study visit indicated. In addition, calcium, phosphate, lactate dehydrogenase (LDH), total protein, albumin, and calculated creatinine clearance **(using the Cockcroft-Gault method)** values must be obtained **from all** subjects at study entry and **from Arm** B subject at entry into Step 2.

6.3.8 Liver Function Tests

Total bilirubin, AST (SGOT), ALT (SGPT), alkaline phosphatase, and indirect bilirubin.

6.3.9 Lipids

Nonfasting triglyceride **(TG)** levels must be obtained from all subjects at entry and at week 48 (or final visit). From Arm A subjects, nonfasting TG levels must also be obtained at weeks 12 and 24. **F**rom Arm B subjects**,** **n**onfasting TG **levels** **must also** be **obtained** at Step 2 entry and at the following “+” weeks: +12 weeks, +24 weeks. If the TG level is > 1000 mg/dL, a fasted test is required within 2 weeks.

Fasting is defined as no food or liquids except water with medications for 8 hours prior to evaluation.

6.3.10 Pregnancy Test

For women of reproductive potential: Serum or urine β-HCG (urine test must have a sensitivity of 50 mIU/mL).

NOTE: A pregnancy test should be performed within 7 days prior to registration to Step 2 to ensure that an appropriate ART is administered.

6.3.11 Immunologic Studies (See Appendix **III**)

CD4+/CD8+

The screening absolute CD4+/CD8+ count and percentages must be obtained within 30 days prior to entry from a laboratory that is CLIA-certified **(for ACTUs) or DAIDS-approved (for ICTUs).** During the study, all **ACTU** laboratories must possess a CLIA certification or its equivalent and must be certified by the DAIDS Immunology Quality Assurance (IQA) Program; **all ICTU laboratories must** **be** DAIDS-approved. Eligibility for study participation based on bacterial infection will be determined by the screening measurement.

Evaluations for CD4+ and CD8+ cell counts and subset percentage evaluations should be performed at the same laboratory, if possible, for baseline calculation and throughout the course of the study.

Because of the diurnal variation in CD4+ and CD8+ cell counts, determinations for individual subjects should be obtained consistently in either the morning or the afternoon throughout the study, if possible.

NOTE: Each time a CD4+/CD8+ measurement is obtained, the local laboratory must perform a WBC and differential from a sample obtained at the same time.

VIROLOGIC STUDIES (See Appendix **III**)

6.3.12 Plasma HIV-1 RNA

The material in this section applies only to ACT**Us**. **ICTUs** will follow site-specific instructions supplied to them by the study team.

Plasma for all **on-study** HIV-1 RNA tests will be collected and shipped in real time to John Hopkins Laboratory.

HIV-1 RNA for subjects entering Step 1 must have been obtained at least 24 hours and up to 28 days prior to study entry. If an Arm B subject has received a HIV-1 RNA test within the 4 weeks before entry into Step 2, this value can be used for the screening sample.

NOTE: The results of the pre-entry (or screening) viral loads are NOT necessary prior to study entry.

On-study HIV-1 RNA quantitation **must** be done in real time.

HIV-1 RNAs **must** also be performed within 2-4 weeks following a suspected virologic failure.

Virologic failure is defined as any of the following:

- - Failure to decrease 1 log by 8 weeks after initiation of AR**T**. (Confirmed within 2-4 weeks after the value obtained after 8 weeks of AR**T**.)

OR

- - - - An increase of 1 log from nadir at any time in the study after 8 weeks of AR**T**. (Confirmed within 2-4 weeks after the first elevated value.)

OR

- - - - Failure to achieve < 500 copies/mL after 24 weeks of AR**T**. (Confirmed within 2-4 weeks after the value obtained after 24 weeks of therapy.)

NOTE: In defining virologic failure, the procedure for calculating the mean baseline viral load can be found on the A5164 PSWP.

6.3.13 Genotyping

The material in this section applies only to ACT**Us**. **ICTUs** will follow site-specific instructions supplied to them by the study team.

At study entry, three additional 1-mL aliquots of plasma (taken from the stored samples drawn for Stored Plasma and PBMCs, section 6.3.14) must be stored at the site for possible shipment during the trial to the Stanford University Virology Support Laboratory (VSL) in the event of a confirmed virologic failure.

During the study, an additional plasma sample (for genotypic testing) must be drawn at the same time the second HIV RNA sample is obtained to confirm virologic failure (section 6.3.12). This should occur within 2-4 weeks after the first RNA sample meets the definition of potential virologic failure.

If virologic failure is confirmed by the second RNA, both the entry (stored) sample that was obtained for genotype testing and the repeat genotype sample taken with the second RNA sample to confirm virologic failure **should** then be shipped to Stanford University VSL by sites that intend to use the results of real-time genotyping. If a real-time genotype will not be requested, sites must store and ship samples as directed in the Laboratory Processing Chart.

NOTE: If an investigator feels that a subject should have his/her genotype tested even though virologic failure (as defined by this protocol) has not been observed, the testing may be performed with the permission of the study chair or vice chair. Extra specimens should be obtained for this purpose in addition to what is allocated for the testing required by the protocol.

6.3.14 Stored Plasma/PBMC

The material in this section applies only to ACT**Us**. ICTUs will follow site-specific instructions supplied to them by the study team.

Collection, processing, and shipping instructions are presented in Appendix **III**.

PBMCs and plasma must also be collected at the time of suspected diagnosis of IRIS.

- - 1. QUESTIONNAIRES
  - Multi-Dimensional Health Status Questionnaire

This questionnaire will take 5-10 minutes to complete.

- - Adherence Questionnaire

This questionnaire will take 5-10 minutes to complete. Adherence to all ARVs (not just study-provided ones) must be reported. Subjects who are not taking any ARVs should be instructed to answer ‘no’ to ARV questions.

NOTE: For Step 1 Arms A and B, the forms are required at weeks 0, 8, 16, 32, and 48. For Step 2 Arm B, the forms are required at entry to Step 2 and weeks +8, +16, +32, and at final study visit.

6.3.16 Subjects Contact for Follow-up

Sites are strongly encouraged to communicate with subjects (in person or by phone) at the time points noted in Schedule of Events, and also to use any tracking mechanism, outreach program, or case management program at any other time sites feel are necessary. Appendix **II** should be used as a guide.

### 6.4 Off-Study Drug Requirements

Additional safety monitoring and reporting of serious adverse experiences (SAEs) continues to be required upon completion or discontinuation of study-provided or study-recommended drugs regardless of whether a protocol follow-up period is scheduled to occur. As specified in the Division of AIDS *Serious Adverse Experience (SAE) Reporting Manual*, **dated August 28, 1998,** adverse experiences occurring during the immediate 8-week period after the last dose of study-provided or study-recommended drugs which meet SAE reporting requirements must be reported to DAIDS Safety Office through the Regulatory Compliance Center (RCC). Additionally, after 8 weeks OFF study-provided or study-recommended drugs, there are four types of events that must be reported to the Safety Office if the relationship to the study-provided or study-recommended drug is assessed by the site physician as definitely, possibly, or unable to judge: DEATHS, NEW ONSET CANCERS, CONGENITAL ANOMALIES, AND PERMANENT DISABILITIES.

## 7.0 GENERAL PRINCIPLES OF ANTIRETROVIRAL THERAPY MANAGEMENT

### 7.1 Immune Reconstitution Inflammatory Syndrome

Reports of an immune reconstitution inflammatory syndrome (IRIS) have been associated with the initiation of ART in patients with advanced immunodeficiency syndrome. The exact relationship and predictors of this syndrome are not well established, nor has the optimal management of the syndrome been established, but there is a growing consensus that ART should not be stopped in the face of what might be an IRIS unless the treating clinician feels that the condition is severe or potentially life-threatening. Use of anti-inflammatory agents and/or corticosteroids may be indicated and should be used at the discretion of the treating physician. If the subject has PCP and **is** taking steroids, **he/she** must complete the entire treatment.

Definition of IRIS to be used in A5164:

Clinical evaluations that lead to IRIS diagnoses **may** occur at either scheduled monthly visits or nonscheduled visits. IRIS **is** defined as follows **for this study**:

- Evidence of an increase in CD4+ cell count and/or a decrease in the HIV-1 viral load in response to starting ART

AND

- Symptoms that are consistent with an infectious/inflammatory condition and temporally related to initiation of ART

AND

- Symptoms that cannot be explained by a newly acquired infection, the expected clinical course of a previously recognized infectious agent, or the side effects of ART itself.

All diagnoses of IRIS based on this definition must be reported as an “Other Clinical Event” to facilitate a case-by-case review by the A5164 chair and/or vice chair prior to analysis.

### 7.2 General Guidelines for Toxicity Management

Only toxicities related to the study-provided drugs (LPV/RTV, **FTC/TDF, or** d4T) and the study-recommended drug**s** (3TC **and FTC**) (not supplied by study) will be considered in the toxicity management section. The grading system is located in the Division of AIDS Table for Grading **Severity of** Adult Adverse Experiences**,** **August 1992,** which can be found on the Division of AIDS Regulatory Compliance Center Web site: <http://rcc.tech-res-intl.com/members/aactg.htm>.

For the purposes of reporting adverse events (AE) for laboratory values of creatine kinase (CK), the following supplemental grading scale will be applied in this study:

Grade 1: 3 - < 6 x ULN

Grade 2: 6 - < 10 x ULN

Grade 3: 10 - < 20 x ULN

Grade 4: ≥ 20 x ULN

Because of the acute illnesses seen in subjects who will be enrolled in this **study**, the number of possible drug interactions, and the heterogeneity of this population, **sites should** adhere to the following general guidelines for toxicities possibly related to the specified antiretroviral regimen. Although an initial regimen is recommended in this study, substitution of components or the entire regimen is permitted. Suggestions of “first line” substitutions are EFV for LPV/RTV, ZDV for d4T, and tenofovir for 3TC or d4T or any other FDA-approved ARV at the discretion of the site investigator.

Actual management of each subject will be left to his/her primary physician’s discretion.

General Guidelines**:**

- Frequent stopping and starting of medication may lead to drug resistance. Therefore, every effort should be made to continue subject on ART as much as possible. The primary physician should rule out other possible causes of the symptoms before discontinuing ART. When possible, concomitant medications should be held first at the discretion of the primary physician if he/she suspects they are contributing to the toxicity. The subject should never be maintained on two or fewer ARV agents for more than 36 hours; this precaution is recommended to prevent drug resistance from developing.
- Recurrence of treatment-limiting toxicity on rechallenge will result in permanent discontinuation of the putative responsible agent.
- If a subject has had two changes in regimen during the initial 4-week period wherein more than one drug was changed, **the site should** notify the team if a third change in regimen is contemplated. This precaution is recommended to prevent subject from being exposed to too many different ARVs in a short period of time.
- Relevant clinical and laboratory tests will be repeated as necessary until there is final resolution or stabilization of the toxicity.

The following guidelines are provided for management of toxicities that may develop with the recommended regimen.

One or more of the following can occur:

1. the doses may be modified or
2. one or more of the ARV drugs may be held or
3. one or more of the ARV drugs may be switched.

### 7.3 Dose Modification Instructions

Dose reductions for d4T are indicated in the table below:

| DRUG | INITIAL DOSE | DAILY DOSE | REDUCED DOSE | DAILY REDUCED DOSE |
| --- | --- | --- | --- | --- |
| d4T (> 60 kg) | 40 mg q 12 hr | 80 mg | 20 mg q 12 hr | 40 mg |
| d4T (< 60 kg) | 30 mg q 12hr | 60 mg | 15 mg q 12 hr | 30 mg |

No dose reductions are allowed for LPV/RTV**, FTC/TDF, 3TC, or FTC** for toxicity management.

### 7.4 Toxicity Management Criteria and Procedures for Modification of Symptoms

The following are general guidelines to assist with toxicity management, but actual management of each subject will be left to the site investigator’s discretion.

7.4.1 Grade 1 or 2

Subjects experiencing Grade 1 or 2 **AEs** or toxicity may continue ARV drugs without alteration of the dosage except **in the case of** Grade 2 peripheral neuropathy (section 7.4.7) (d4T) and for nausea and vomiting, anemia, or neutropenia as outlined in sections 7.4.5 and 7.5.7. One or more drug substitutions may be made at the discretion of the site investigator.

7.4.2 Grade 3

Subjects who develop a Grade 3 **AE** or toxicity judged to be ARV drug-related, except for those outlined below, should have one or more of their ARV study drugs switched or the entire regimen held, at the investigator’s discretion. The subject should be reevaluated weekly until the AE returns to Grade ≤ 2, at which time the ARV drugs may be reintroduced at the discretion of the site investigator or according to standard practice.

If the same Grade 3 AE recurs, any ARV drugs must be permanently discontinued if the investigator considers the adverse event related to those specific ARV drugs.

Subjects experiencing Grade 3 **AEs** requiring permanent discontinuation of any ARV drug should be followed weekly until resolution of the AE in addition to their regular study visits. The treatment regimen should be modified, and subjects should be encouraged to remain in the study.

7.4.3 Grade 4

Subjects who develop a Grade 4 **AE** or toxicity will have the presumed causative ARV drug permanently discontinued and another drug will be substituted. If it is not possible for the investigator to discern the causative agent, then all ART must be discontinued. A new regimen may be chosen at the discretion of the site investigator. Subjects experiencing Grade 4 AEs requiring permanent discontinuation of ART should be followed weekly in addition to their regular study visits until resolution of the AE.

Subjects with Grade 4 asymptomatic laboratory abnormalities, e.g., CK elevation, may continue ART if the investigator has compelling evidence that the toxicity is NOT related to the ARV drugs.

7.4.4 Rash

Grade 1 or 2 Rash

Study treatment should continue without interruption. Subjects with a Grade 1 or 2 rash may be treated symptomatically with permitted antipyretic, antihistamine, and/or non-steroidal anti-inflammatory medications, but should be monitored closely by the site investigator.

Grade 3 or 4 Rash

ART should be held for any Grade 3 or 4 rash unless the rash is determined to be unrelated to study medications. Upon resolution to Grade ≤ 1, the site should communicate with the protocol chairs.

7.4.5 Nausea and Vomiting

Grade 1 or 2

ART should continue without interruption. Subjects with Grade 1 and 2 nausea or vomiting may be treated symptomatically with permitted oral antiemetic therapies or antiemetic suppositories. Subjects should be instructed to take medications with food. Persistent nausea or vomiting despite dose reduction **may** lead to discontinuation of the regimen**, if** appropriate.

Investigators should consider the diagnoses of pancreatitis or lactic acidosis syndrome and evaluate subjects for these events if clinically indicated.

Grade ≥ 3

Subjects with Grade ≥ 3 nausea and vomiting should interrupt all ARV drugs until the toxicity grade returns to Grade ≤ 2 or to baseline. Therapy should be resumed using the full doses of ARV. If Grade ≥ 3 nausea and vomiting recurs upon the resumption of ART, these should again be interrupted and alternative antiretroviral agents should be considered.

Grade ≥ 3 Associated with Other Signs and Symptoms of Pancreatitis or Elevations in Amylase or Lipase

Refer to sections 7.4.8 and 7.5.1.

7.4.6 Diarrhea

Grade 1 or 2

ART should continue without interruption. Subjects with diarrhea of any toxicity grade may be treated symptomatically with permitted antimotility agents.

Grade 3 or 4

Diarrhea that persists for > 7 days, is unresponsive to antimotility agents, and for which an alternative etiology (e.g., infectious diarrhea) is not established, all ART should be interrupted until resolution of diarrhea to Grade ≤ 2 or baseline. If Grade ≥ 3 diarrhea recurs upon the resumption of ART, all ART should be interrupted and alternative ART should be selected at the discretion of the site investigator.

7.4.7 Peripheral Neuropathy

Grade 1

Study medications may be continued.

Grade 2

d4T should be held until toxicity is reduced to Grade ≤1 and may be re-introduced at the reduced dose.

If Grade 2 toxicity recurs despite dose reduction, d4T will be permanently discontinued and another nucleoside should be substituted. If Grade 2 toxicity does not recur on the decreased dose, the d4T dose may be increased at the discretion of the site investigator. If Grade 2 toxicity then recurs, d4T will be permanently discontinued and another nucleoside should be substituted.

Grade ≥ 3

d4T should be permanently discontinued. Symptomatic treatment may be provided at the discretion of the site investigator.

7.4.8 Symptomatic Pancreatitis

A diagnosis of pancreatitis should be considered if clinical symptoms such as nausea, vomiting, or abdominal pain are present. Subjects with these signs and symptoms should have ART held and be evaluated for the presence of pancreatitis through the use of diagnostic testing, which should include serum lipase measurements and may include pancreatic imaging via abdominal ultrasound and/or CT scanning, as clinically warranted.

If a diagnosis of pancreatitis is confirmed, appropriate therapy should be initiated and d4T should be permanently discontinued. The investigator should not consider reintroduction of **ART** until clinical signs and symptoms of pancreatitis have resolved and pancreatic lipase values return to Grade ≤ 1. An alternative NRTI or TDF should be substituted for d4T, if possible.

7.4.9 Neuromuscular Weakness or Toxicity

Recent reports of ascending lower extremity weakness have been reported and appear to be associated with d4T use. This profound motor weakness is suggestive of a Guillain-Barre-like syndrome and appears to be associated with elevated serum lactic acid levels. In subjects experiencing any form of muscular weakness while on study drugs, a serum lactate level should be ordered and the study drugs should be discontinued. Reinitiation of ARV both in terms of the antiretroviral agents and timing will be left to the medical opinion of the treating physician. It is recommended that d4T not be reinstituted as part of the subject’s ART [34].

### 7.5 Toxicity Management for Laboratory Abnormalities

7.5.1 Asymptomatic Hyperlipasemia or Pancreatic Amylasemia

Grade 2 Asymptomatic

Subjects with Grade 2 asymptomatic elevations in lipase should be carefully evaluated and followed closely. Repeat measurement of lipase should be **performed** within 4 weeks. Persistent asymptomatic Grade 2 elevation of lipase should be discussed with the protocol team and should be followed closely with repeated levels every 2- 4 weeks. Subjects should be educated regarding the symptoms of pancreatitis. Asymptomatic elevation in total serum amylase in the absence of elevation of lipase does not require further evaluation.

Grade ≥ 3 Asymptomatic

Subjects with Grade ≥ 3 elevations in serum lipase without clinical symptoms of pancreatitis should have all ART held until toxicity returns to Grade ≤ 2 or baseline, then all ART may be restarted at full dose.

If Grade ≥ 3 toxicity persists for more than 14 days despite interruption of ART, or recurs after reintroduction of ART, **sites should** consider permanent discontinuation of all study medications, and a new potent ART should be selected at the discretion of the site investigator. Protocol chairs should be notified of new regimen by site investigator.

7.5.2 Symptomatic Hyperlactatemia

Symptomatic hyperlactatemia will be defined as new, otherwise unexplained, and persistent occurrence for >2 weeks of one or more of the following:

- nausea and vomiting
- abdominal pain or gastric discomfort
- abdominal distention
- increased LFTs
- unexplained fatigue
- dyspnea

plus a confirmed lactate level > 2 × ULN.

If the lactate value is > 2 × ULN, **sites should** obtain a confirmatory lactate value as soon as possible, preferably within 1 week. ART should be interrupted immediately if the confirmatory value remains > 2 × ULN or if the site is unable to obtain a confirmatory value within 1 week. Determine lactate levels every 4 weeks until the lactate value returns to normal, at which time a new ART may be constructed in consultation with the protocol team.

Management of a symptomatic lactate value ≤ 2 x ULN will proceed at the discretion of the site investigator. However, any modification of a subject’s antiretroviral regimen should be made in consultation with the protocol team. Since some of the symptoms of hyperlactatemia are vague and may be present in many subjects (e.g., fatigue), repeated lactate determinations are advised.

NOTE: See the ACTG Web site for guidelines for the collection of lactate specimens: http://aactg.s-3.com.

7.5.3 Asymptomatic Hyperlactatemia

If the lactate value is > 2 × ULN, a confirmatory lactate value must be obtained as soon as possible, preferably within 1 week. Subjects should be evaluated for any of the symptoms or findings listed in section 7.5.2. ART must be discontinued for all subjects with a confirmed lactate value of > 4 × ULN, whether or not the subject has any of the symptoms listed in section 7.5.2. Lactate levels should be obtained every 4 weeks until the level returns to **≤** 2 × ULN, at which time a new ART may be initiated in consultation with the protocol team.

If a lactate level of >2 - 4 × ULN is confirmed and the subject remains asymptomatic, the subject should be followed with repeat lactate levels every 4 weeks. Serial monitoring may be discontinued if two or more lactate measurements are ≤ 2 × ULN and the subject remains asymptomatic.

NOTE: See the ACTG Web site for guidelines for the collection of lactate specimens: [http://aactg.s-3.com](http://aactg.s-3.com/).

7.5.4 Hypertriglyceridemia

Subjects who experience asymptomatic triglyceride elevations may continue to receive study medications. Nonfasting triglyceride levels > 1000 mg/dL will require subjects to have confirmatory “fasting” test for toxicity. A confirmatory fasting triglyceride level should be obtained within 2 weeks and must be obtained prior to the institution of medical therapy for the hyperlipidemia.

Subjects with fasted asymptomatic triglyceride elevation should be counseled on dietary modification. Oral antihyperlipidemic agents should be considered at the discretion of the site investigator (and documented on the concomitant medication CRF). Guidelines for the treatment of hyperlipidemia are available on the ACTG Web site ([http://aactg.s-.com/members/download/ other/ metabolic/Chol.rtf](http://aactg.s-3.com/members/download/other/metabolic/Chol.rtf)). Resin-binding agents, such as cholestyramine and colestipol, should be avoided as they may interfere with absorption of PIs as well as the absorption of other medications and fat-soluble vitamins.

However, if there is Grade 4 hypertriglyceridemia and it persists despite medical intervention, discontinuation of the ARV drugs should be considered and a new regimen selected for introduction when the toxicity returns to < Grade 2.

7.5.5 Hyperglycemia

Fasting hyperglycemia of > 110 to 125 mg/dL is considered evidence of impaired glucose tolerance. A fasting blood glucose level above 126 mg/dL is highly suggestive of diabetes mellitus. Subjects with fasting hyperglycemia may continue **ART** at the discretion of the investigator. A confirmatory fasting glucose must be obtained within 4 weeks and prior to the institution of medical therapy. Hyperglycemia may be treated with oral hypoglycemic agents or insulin according to standard guidelines.

7.5.6 AST/ALT Elevations

Grade 3

Study medications may be continued for asymptomatic Grade 3 AST/ALT elevations at the discretion of the site investigator. Careful assessments should be done to rule out the use of alcohol, non-ARV medication-related drug toxicity, or viral hepatitis as the cause of the Grade 3 elevation. The possibility of lactic acidosis syndrome should also be explored.

Grade 4

All **ART** should be held for AST or ALT Grade 4 elevations until the toxicity grade returns to Grade ≤ 2. If Grade 4 elevation in AST or ALT recurs, alternative ART should be selected at the discretion of the site investigator.

7.5.7 Anemia/Neutropenia

Subjects with anemia or neutropenia may be treated with blood transfusion and/or erythropoietin or G-CSF (Neupogen®) at the discretion of the site investigator.

Further reductions or discontinuations of ARVs may be necessary. **These** will be at the discretion of the site investigator.

7.5.8 Hypophosphatemia (for participants on FTC/TDF)

For Grades 1 and 2 hypophosphatemia, phosphate should be repeated as soon as possible (within 2 weeks is optimal), and FTC/TDF may be continued without other signs of renal tubular acidosis at the discretion of the site investigator.

For Grades 3 and 4 hypophosphatemia, the phosphate should be repeated preferably within 1 week. Supplemental phosphate or foods high in phosphates should be given and other causes of low phosphate should be investigated. If Grade 3 or 4 hypophosphatemia persists discontinue FTC/TDF permanently.

7.5.9 Creatinine Clearance for Subjects Receiving FTC/TDF

Calculated creatinine clearance <50 mL/min

- Calculated creatinine clearance must be repeated as soon as possible (at most within 1 week) of a value of <50 mL/min, calculated using the following formula:

{([140 - age(yr)]x [weight(kg)]) ÷[72 x serum Cr(mg/dL)] } (x 0.85 for females)

Study treatment may be continued while repeating calculated creatinine clearance levels as long as the subject is asymptomatic.

- For confirmed level of <50 mL/min, FTC/TDF should be permanently discontinued and other antiretroviral agents held.
- Subjects should be followed as medically indicated until the calculated creatinine clearance level returns to ≥60 mL/min, after which ARV therapy may be resumed with ddI or ZDV substituted for TDF. FTC may be resumed, if available.

## 8.0 CRITERIA FOR PREMATURE TREATMENT OR STUDY DISCONTINUATION

### 8.1 Premature Discontinuation of ART

- Drug-related toxicity if described in section 7.0 (Toxicity Management)
- Breastfeeding
- Use of prohibited concomitant medications (see section 5.5.1) **if contraindicated for subject’s regimen.**
- Request of the primary care provider if s/he thinks the treatment is no longer in the best interest of the subject.
- **Request by the subject to discontinue ART.**
- Clinical reasons believed life-threatening by the physician, even if not addressed in the toxicity management of the protocol.
- Failure to confirm HIV diagnosis

### 8.2 **Premature** Study Discontinuation

- At the discretion of the ACTG, NIAID, investigator, or pharmaceutical supporter.
- Request by the subject to withdraw.
- Request of the primary care provider if s/he thinks the study is no longer in the best interest of the subject.
- Subject judged by the investigator to be at significant risk of failing to comply with the provisions of the protocol as to cause harm to self or seriously interfere with the validity of the study results.
- Failure to confirm HIV diagnosis.

## 9.0 STATISTICAL CONSIDERATIONS

### 9.1 General Design Issues

This is an open-label, randomized study comparing the strategy of immediate versus deferred initiation of antiretroviral therapy in HIV-infected subjects presenting with acute opportunistic infections or serious bacterial infections (OI/BI). The target sample size is 282 subjects, 141 per arm. The randomization will be stratified by CD4+ cell count and by OI/BI (PCP versus bacterial infections versus other OIs). We anticipate that accrual will be completed **by the end of 2006**.

### 9.2 Endpoints

- - 1. Primary

A composite endpoint incorporating:

- - Survival
  - Recurrence of presenting OI/BI or incidence of new AIDS-defining events
  - HIV-1 plasma viral load at 48 weeks
    1. Secondary
       1. HIV-1 plasma viral load at all time points up to and including week 48.
       2. CD4+ counts at all time points up to and including week 48.
       3. Changes in ARV regimen for lack of efficacy.
       4. Efficacy of treatment and clinical outcomes for specific OI/BI, including:
- Duration of treatment for OI/BI (median days, percent requiring extension of induction treatment)
- Complications of OI/BI treatment and course
- Incidence and duration of hospitalization for OI/BI (if required)
- Rate of relapse/recurrence of OI/BI
- Incidence of immune reconstitution inflammatory syndromes (IRIS) and impact on outcomes in the two arms
  - - 1. Safety and tolerability measured by:
- Grades 3 and 4 signs and symptoms and laboratory toxicities.
- AR**T** and OI/BI treatment changes and dose modifications due to toxicities.
- IRIS

9.2.2.6 HIV-1 drug resistance over time (genotype).

9.2.2.7 Health care resource use

Total inpatient days, ER visits will be compared in the two groups.

9.2.2.8 Quality of life and functional status outcomes

Overall self-reported QOL as well as functional status will be compared in the two groups at 48 weeks.

9.2.2.9 Adherence

Self-reported adherence to all ARVs over the study period will be assessed and examined for relationship with primary study outcomes including death, progression, and viral suppression.

### 9.3 Sample Size and Accrual

Primary objective: The primary endpoint of this study is an ordered categorical variable that combines survival, AIDS progression, and HIV-1 virologic control at week 48. The categories are: C1, death or alive with recurrence of the presenting OI/BI or a new AIDS-defining event; C2, alive with HIV-1 viral load of ≥ 50 copies/mL and without an OI/BI recurrence or a new AIDS-defining event; and C3, alive with viral load <50 cp/mL and without an OI/BI recurrence or a new AIDS-defining event. Based on the data as described in the introduction, we estimate the true C1 rate within the study time period either from the acute OI/BI or other causes among subjects on the deferred arm to be between 15 and 30%. We consider a drop by 1/3 to be clinically important to see in the immediate therapy arm. We estimate the true rate of subjects in the deferred arm in C3 to be 30-40%. Because of the difficulty in managing antiretroviral therapy during the immediate acute OI/BI period, we feel that a 20% improvement to 50 or 60% of subjects in the immediate therapy arm in C3 is clinically important. Power calculations were based on modeling C1, C2, and C3 as ordered categorical outcomes (StatXact-4), and assuming a 2-sided alpha = .05-level test. Under these assumptions and with endpoint information on all subjects, 110 subjectsper arm would provide at least 80% power for all combinations of C1, C2, and C3 outlined above. For example, if C1, C2, and C3 are .15, .45, and .40 in the deferred arm and .10, .30, and .60 in the immediate arm, the power is 81% with 110/arm. If the proportions in C1, C2, and C3 are .15, .55, and .30 versus .10, .40, and .50 in the two arms, respectively, the power is 83%. These calculations do not incorporate stratification so should represent a lower bound on power. A benefit of this design is that it has reduced power with a paradoxical result (e.g., increased death rate but increased viral control with immediate therapy), decreasing the likelihood of rejecting the null hypothesis should this be the true situation.

Subjects who discontinue antiretroviral treatment are to be followed to 48 weeks to provide as complete data as possible on the virologic, OI/BI, and other AIDS-defining endpoints. Nonetheless, prior experience indicates that some subject will completely discontinue the study before 48 weeks or confirmation of a C1 endpoint. Mortality should be completely ascertainable, and we feel it highly unlikely that subjects who drop out of the study will have HIV viral load <50 at week 48. Thus, subjects who drop out of the study entirely prior to week 48 and who provide no week 48 sample for viral load or no information on recurrences or other AIDS-defining events will be assigned to the C2 (intermediate) outcome category for the primary intent-to-treat analyses (hereafter termed “primary assignment method”). The primary assignment method penalizes each arm equally for subjects who drop out. OI/BIs and AIDS-defining events may occur in those who drop out that we do not record. An important confirmatory analysis will be performed. The “alternative assignment method” will be to code subjects who drop out as C1 in the “best” arm (i.e., the arm with highest C3) and as C2 in the “worst” arm. As noted above, we feel it highly unlikely that subjects who drop out of the study will have HIV viral loads of <50 at week 48. To calculate the power for the two reassignment strategies, we assume all losses are in C1 and C2. We will power the study to have adequate power with 10% dropout under both methods. With 141 subjects per arm (282 total), there will be around 77% power with 10% dropout in C1 and C2 under the alternative assignment method to detect a range of options for true underlying rates of C1, C2, and C3 that have a 20% absolute difference in C3. This sample size has over 90% power to detect these differences using the primary assignment method with 10% dropout in C1 and C2, and 82-84% power for the primary assignment method with 20% dropout in C1 and C2. The power is negligible with the alternative assignment method with 20% dropout in C1 and C2 because the method heavily penalizes the best arm, resulting in extremely paradoxical results. The sample size has been increased by 5% to account for a group sequential design. Subjects who enter the study and subsequently are found not to have HIV infection, will not be included in any analysis.

Secondary objectives: The Type I (false positive) error rate for statistical tests for secondary objectives will not be adjusted for multiple testing. Additionally, some **analyses** may be performed on subsets of the data. As such, these evaluations should be considered to be exploratory.

A number of the secondary objectives will be evaluated within subsets of the entries to A5164. For example, the comparison between arms of the efficacy of treatment for OI/BIs will be restricted to analyses within the most common, probably pneumonia and PCP. Similarly, the development of resistance mutations can only be evaluated in subjects with detectable HIV-1 RNA. For a range of sample sizes of subsets (assuming equal sample sizes in groups for two-sample tests), the following presents:

1. the largest difference between two proportions that would need to be true for there to be a minimum of 80% power (2-sided alpha=.05). The power is lowest when the proportions, given in [.41, .59], are near 50%. If the true proportions are closer to 0 or 1.00, there will be higher power to detect the same absolute difference.
2. the difference between the means of two normally distributed samples (e.g., CD4+) that can be detected with 80% power (2-sided alpha=.05). The difference is expressed as a proportion of the common standard deviation of the variable.
3. the widest width of a 95% confidence interval (CI) around an observed proportion for the given sample size in one group. This width is widest at an observed proportion of 0.50. If observed proportions are closer to 0 or 1.00, the width is narrower for the same sample size.

| N per group | 2-sample  binomial (1) | 2-sample  normal (2) | 1-sample  binomial (3) |
| --- | --- | --- | --- |
| 141 | .18[.41, .59] | .34X s.d | .17 |
| 130 | .18[.41, .59] | .35X s.d. | .18 |
| 120 | .20 [.40, .60] | .37 X s.d. | .19 |
| 90 | .23 [.39, .62] | .42 X s.d. | .22 |
| 60 | .27 [.36, .63] | .51 X s.d. | .27 |
| 30 | .39 [.31, .70] | .73 X s.d. | .38 |

Assuming as does A5001 that the SD of changes in the overall QOL score (measured on a 1-100 scale) is 20, this study will have 80% power to detect a difference of 8 points between the two arms.

### 9.4 Monitoring

This study will be reviewed at least annually by the NIAID Data Safety Monitoring Board (DSMB). We anticipate that accrual will be complete**d by the end of 2006**. The primary three-category endpoint is only ascertainable with 48 weeks of follow-up. There will be one formal interim analysis when approximately 50% of entries have 48 weeks of follow-up. Other annual reviews by the DSMB will be provided detailed information on safety (including mortality, regimen tolerability, compliance with visits, and specimen collection and retention).

The interim and final analyses of the primary endpoint will be evaluated with boundaries defined by an O’Brien-Fleming use function. The boundary will reflect the actual information at the interim, and the final analysis will be performed with a boundary adjusted for the sequential monitoring. Secondary objectives relating to OI/BI outcomes that are analyzed in conjunction with the primary endpoint will be evaluated at the interim analysis using the same boundary as the primary endpoint, and the final analyses of these endpoints will be evaluated using the adjusted boundary. A number of the secondary objectives will not be evaluated until the end of the study either because they are based on subgroups that are retrospectively identified or because they are not related to safety or the primary outcomes.

Reports on toxicity by arm will be provided to the Division of AIDS Medical Officer **following the standard schedule for DSMB reviewed studies**. In addition, periodic reports of data pooled over arm will be provided to the team and the RAC that detail accrual, subject status, delinquency of forms and specimens, and toxicity. The form and schedule of these will be detailed in the Monitoring Plan to be developed when the study is activated.

### 9.5 Analyses

This is a study of the strategy of attempting to provide ART during the initial treatment of the OI/BI vs. attempting to defer ART until the OI/BI has been treated. As such, all subjects appropriately enrolled in the study will be included in the primary analyses. Only subjects who are discontinued for not having an HIV infection on confirmatory testing will be excluded from these primary analyses.

The primary outcome variable (death, progression of AIDS, and alive with VL ≥ 50/alive with VL< 50), stratified by CD4+ count and OI/BI at entry, will be analyzed using methods for ordinal data. Techniques such as continuous-ratio logit models [35] are most appropriate as they do not make the assumption of a constant odds ratio between adjacent categories. As detailed elsewhere, subjects who are alive at week 48 (or were known to be alive when completely lost to follow-up) and provide no week 48 sample for viral load will be grouped with the middle category for the primary intent-to-treat analyses. Additionally, subjects who enter with a presumed OI/BI that is not confirmed by post-entry laboratory or other results will be included in the primary intent-to-treat analyses. This will most closely reflect clinical practice where implementation of the immediate therapy strategy would often have to be initiated before confirmatory cultures and other tests were completed. These will be evaluated with a boundary (p-value) appropriately adjusted for the sequential design as noted above.

Secondary supporting analyses of the primary outcome variable will include the alternative assignment method, described above. Other secondary analyses will exclude subjects with no week 48 viral load data, and others will exclude subjects who were shown not to have target OI/BIs based on post-entry laboratory results. Additional, secondary analyses will be done limited to subjects who implemented the assigned policy, i.e., started medication promptly in Arm A (include those who started as soon as possible after medical contraindications were resolved) and those who started between week 4 (day 28) and 32 (day 224) of date **after** enrollment in Arm B.

Secondary objectives will be evaluated with statistical techniques appropriate to the data type, including but not limited to logistic regression and GEE (dichotomous endpoints), and ANOVA, ANCOVA, and GEE (continuous endpoints). Some will consider change over early time points (weeks 4, 8, and 12) as well as between baseline and target time points such as weeks 24 and 48. Continuous variables may be dichotomized based on “conventional” cutpoints (e.g., CD4+ < 200) or at cutpoints suggested by residual analysis or Classification and Regression Tree (CART) analysis. Alternative parameterizations of the endpoints listed in section 9.2 may be evaluated (e.g., time to recurrent OI/BI, DAVG of the HIV-1 viral load). As noted above, except for select analyses of OI/BI endpoints that are reviewed by the DSMB, the alpha-level for these exploratory and hypothesis-generating analyses will not be adjusted for exploratory data analysis and multiple testing.

## 10.0 DATA COLLECTION AND MONITORING AND ADVERSE EXPERIENCE REPORTING

### 10.1 Records to Be Kept

Case report forms (CRF) will be provided for each subject. Subjects must not be identified by name on any CRFs. Subjects will be identified by the Patient Identification Number (PID) and Study Identification Number (SID) provided by the ACTG Data Management Center upon randomization.

### 10.2 Role of Data Management

10.2.1 Instructions concerning the recording of study data on CRFs will be provided by the ACTG Data Management Center. Each ACTU **or ICTU** is responsible for keying the data.

10.2.2 It is the responsibility of the ACTG Data Management Center to assure the quality of computerized data for each ACTG study. This role extends from protocol development to generation of the final study databases.

### 10.3 Clinical Site Monitoring and Record Availability

10.3.1 Site monitors under contract to the National Institute of Allergy and Infectious Diseases (NIAID) will visit participating clinical research sites to review the individual subject records, including consent forms, CRFs, supporting data, laboratory specimen records, and medical records (physicians’ progress notes, nurses’ notes, individuals’ hospital charts), to ensure protection of study subjects, compliance with the protocol, and accuracy and completeness of records. The monitors also will inspect sites’ regulatory files to ensure that regulatory requirements are being followed and sites’ pharmacies to review product storage and management.

10.3.2 The investigator will make study documents (e.g., consent forms, drug distribution forms, CRFs) and pertinent hospital or clinic records readily available for inspection by the local IRB, the site monitors, the NIAID, the Office for Human Research Protections (OHRP), and the pharmaceutical supporters or their designees for confirmation of the study data.

### 10.4 Serious Adverse Experience (SAE) Reporting

This protocol follows standard SAE reporting requirements.

Only study-provided (LPV/RTV, **FTC/TDF, and** d4T) or study-recommended (3TC **and FTC**) drugs, should be taken into account for serious adverse experience reporting. Serious adverse experiences must be documented on the Division of AIDS Serious Adverse Experience (SAE) Reporting Form and submitted to the Regulatory Compliance Center (RCC) Safety Office and are defined in the current Division of AIDS *Serious Adverse Experience (SAE) Reporting Manual*, **dated August 28, 1998***.*

All antiretroviral drugs that the subject was receiving at the time of onset of the AE must be addressed in the assessment of relationships to SAEs.

The Division of AIDS Table for Grading **Severity of** Adult Adverse Experiences**,** **August 1992,** can be found on the RCC Web site: <http://rcc.tech-res-intl.com/members/aactg.htm>

## 11.0 HUMAN SUBJECT**S**

### 11.1 Institutional Review Board (IRB) Review and Informed Consent

This protocol and the informed consent documents (Appendices **IV** and **V**) and any subsequent modifications will be reviewed and approved by the IRB or ethics committee responsible for oversight of the study. A signed consent form will be obtained from the subject (or parent, legal guardian, or person with power of attorney for subjects who cannot consent for themselves, such as those below the legal age **of consent**). The consent form will describe the purpose of the study, the procedures to be followed, and the risks and benefits of participation. A copy of the consent form will be given to the subject, parent, or legal guardian, and this fact will be documented in the subject’s record.

### 11.2 Subject Confidentiality

All laboratory specimens, evaluation forms, reports, and other records that leave the site will be identified by coded number only to maintain subject confidentiality. All records will be kept locked. All computer entry and networking programs will be done with coded numbers only. Clinical information will not be released without written permission of the subject, except as necessary for monitoring by IRB, the NIAID, the OHRP and the pharmaceutical supporters or their designees.

### 11.3 Study Discontinuation

The study may be discontinued at any time by the IRB, the pharmaceutical supporters, the NIAID or other government agencies as part of their duties to ensure that research subjects are protected.

## 12.0 PUBLICATION OF RESEARCH FINDINGS

Publication of the results of this trial will be governed by ACTG policies. Any presentation, abstract, or manuscript will be made available for review by the pharmaceutical supporters prior to submission.

## 13.0 BIOHAZARD CONTAINMENT

As the transmission of HIV and other blood-borne pathogens can occur through contact with contaminated needles, blood, and blood products, appropriate blood and secretion precautions will be employed by all personnel in the drawing of blood and shipping and handling of all specimens for this study, as currently recommended by the Centers for Disease Control and Prevention and the National Institute of Health.

All infectious specimens will be transported using packaging mandated in the Code of Federal Regulations, 42 CFR Part 72. Sites should also refer to individual carrier guidelines, e.g., FedEx, Airborne, for specific instructions.

## 14.0 REFERENCES

1. Hammer SM, Squire KE, Hughes MD et al. A controlled trial of two nucleoside analogues plus indinavir in persons with human immunodeficiency virus infection and CD4 cell counts of 200 per cubic millimeter or less. N Engl J Med 1997; 337:725-33.
2. Hirsch M, Steigbigel R, Staszewski S, et al. A randomized, controlled trial of indinavir, zidovudine, and lamivudine in adults with advanced immunodeficiency virus type 1 infection and prior antiretroviral therapy. JID 1999; 180: 659-665.
3. Center of Disease Control and Prevention Website, <http://www.cdc.gov/>
4. Sham HL, Kempf DJ, Molla A, et al. ABT-378, a highly potent inhibitor of the human immunodeficiency virus protease. Antimicrob Agents Chemother 1998; 42: 3218-24.
5. Kumar GN, Dykstra J, Roberts EM, et al. Potent inhibition of the cytochrome P-450 3A-mediated human liver microsomal metabolism of a novel HIV PI by ritonavir. Drug Metab Dispos 1999; 27(8): 902-8.

6. Clumek N, et al. ABT-378/ritonavir (ABT-378/r) and efavirenz: 16 week safety/efficacy evaluation in multiple PI experienced patients. [Abstract TuPeB3196] In: Abstracts of the XIII International AIDS Conference, Durban, South Africa, July 9-14, 2000.

7. Thompson M, et al. Analysis of duration of virologic response in two phase II studies of ABT-378/ritonavir (ABT-378/r) at 72 weeks. [Abstract TuPeB3197] In: Abstracts of the XIII International AIDS Conference, Durban, South Africa, July 9-14, 2000.

8. Zolopa A, et al. Predictors of response to ritonavir (RTV)/saquinavir (SQV) antiretroviral therapy (ART) in patients for whom nelfinavir (NFV) failed: a multicenter clinical cohort study. [Abstract WePeB4173] In: Abstracts of the XIII International AIDS Conference, Durban, South Africa, July 9-14, 2000.

9. Gulick RM, Hu XJ, Fiscus SA, et al. Randomized study of saquinavir with ritonavir or nelfinavir together with delaviridine, adefovir, or both in human immunodeficiency virus-infected adults with virologic failure on indinavir: AIDS Clinical Trials Group Study 359. J Infect Dis 2000; 182: 1375-84.

10. Kempf, et al. Identification of clinically relevant phenotypic and genotypic breakpoints for ABT-378/r in multiple PI-experienced, NNRTI-naïve patients. [Abstract 89] In: Abstracts of the 4^th^ International Workshop on HIV Drug Resistance and Treatment Strategies, Sitges, Spain, June 12-16, 2000.

1. Griffith BP, Brett-Smith H, Kim G, et al. Effect of stavudine on human immunodeficiency virus type 1 virus load as measured by quantitative mononuclear cell culture, plasma RNA, and immune complex-dissociated antigenemia. J Infect Dis 1996; 173(5):1252-5.
2. Anderson R, Meyer W, Balch F, et al. Anti-viral effects of stavudine (d4T) therapy. [Abstract WeB1010] In: Abstracts of the Eighth International Conference on AIDS/III STD World Congress, The Netherlands; 1993.
3. Murray HW, Squires KE, Weiss W, et al. Stavudine in patients with AIDS and AIDS-related complex: AIDS Clinical Trials Group 089. J Infect Dis 1995; 171(Suppl.2): S123-30.
4. Peterson EA, Ramirez-Ronda CH, Hardy WD, et al. Dose-related activity of stavudine in patients infected with human immunodeficiency virus. J Infect Dis 1995; 171(Suppl.2): S131-9.
5. Spruance SL, Pavia AT, Mellors JW, et al. Clinical efficacy of monotherapy with stavudine compared with zidovudine in HIV-infected, zidovudine-experienced patients. A randomized, double-blind, controlled trial. Bristol-Myers Squibb Stavudine/019 Study Group. Ann Intern Med 1997; 126(5): 355-63.
6. **Blum MR, Begley J, Zong J, et al. Lack of pharmacokinetic interaction between emtricitabine and tenofovir DF when co-administered to steady state in healthy volunteers [Abstract A-1621]. Presented at the 43^rd^ Interscience Conference on Antimicrobial Agents and Chemotherapy; September 14-17, 2003; Chicago, IL.**
7. **Kearney BP, Zong J, Begley J, et al. Bioequivalence of combination tenofovir DF/emtricitabine tablets for one-pill once daily administration [Poster Number 7.3]. 5^th^ International Workshop on Clinical Pharmacology of HIV Therapy; April 1-3, 2004; Rome, Italy.**
8. **Gazzard B, DeJesus E, Campo R et al. The combination of tenofovir DF (TDF), emtricitabine (FTC) and efavirenz (EFV) has significantly greater response vs. fixed dose zidovudine/lamivudine (CBV) and EFV in antiretroviral naïve patients: a 24 week preliminary analysis. [Oral Presentation]. Presented at the 44th Interscience Conference on Antimicrobial Agents and Chemotherapy; 2004 October 30-November 2; Washington DC, USA. Abstract H-1137c.**
9. **Gathe J, Podzamczer D, Johnson M, et al. Once-daily vs. twice-daily lopinavir/ritonavir in antiretroviral-naïve patients: 48-week results [Poster Number 570]. 11^th^ Conference on Retrovirus and Opportunistic Infections; 2004 February 8-11; San Francisco, CA.**
10. **Molina JM, Gathe J, Lim PL, et al. Comprehensive resistance testing in antiretroviral-naïve patients treated with once-daily lopinavir/ritonavir plus tenofovir DF and emtricitabine: 48-week results from Study 418 [Poster Number WePeB5701]. XV International AIDS Conference; 2004 July 11-16; Bangkok, Thailand.**
11. **Podzamczer D, Gathe J, Johnson M, et al. Study 418: Once-daily vs. twice-daily lopinavir/r in antiretroviral-naïve patients: 24-week results [Abstract]. 9^th^ European AIDS Conference; 2003 October 26-29; Warsaw, Poland.**
12. Moore KHP, Barrett JE, Shaw S, et al. The pharmacokinetics of lamivudine phosphorylation in peripheral blood mononuclear cells from patients infected with HIV-1. AIDS 1999; 13(16): 2239-50.
13. Yuen GJ, Lou Y, Bumgarner NT, et al. Equivalence of plasma and intracellular triphosphate lamivudine pharmacokinetics (PK) following lamivudine (3TC) 300 mg once daily compared to lamivudine 150 mg twice daily in healthy volunteers. [poster 269]. 5^th^ International Congress on Drug Therapy in HIV Infection, Glasgow, UK; October 22-26, 2000.
14. Staszewski S, Haberl A, Gute P, et al. Nevirapine/didanosine/lamivudine once daily in HIV-1 infected intravenous drug users. Antiviral Therapy 1998; 3 (Suppl. 4): 55-6.
15. Bowonwatanuwong C, Mootsikapun K, Supparatpinyo S, et al. [Poster 004].The 1^st^ IAS Conference on HIV Pathogenesis and Treatment, Buenos Aires, Argentina, July 8-11 2001.
16. Wislez M, Bergot E, Antoine M, et al. Acute respiratory failure following HAART introduction in patients treated for Pneumocystis carinii pneumonia, Am J Respir and Crit Care Med 2001; 164: 847-51.
17. Shelburne SA 3rd, Hamill RJ. The immune reconstitution inflammatory syndrome. AIDS Rev. 2003 Apr-Jun; 5(2): 67-79.
18. Hamill RJ. Immune restoration syndrome in AIDS and mycoses. Program and abstracts of the 41^st^ Interscience Conference on Antimicrobial Agents and Chemotherapy; December 16-19, 2001; Chicago, Illinois. Abstract 1272.
19. Benveniste O, Blanche P, et al. Paradoxical reactions due to immune restoration during Cryptococcus neoformans infection in AIDS. 41^st^ Interscience Conference on Antimicrobial Agents and Chemotherapy; December 16-19, 2001; Chicago, Illinois. Abstract J-674.
20. Dworkin M, Hanson DL, Navin TR. Survival of patients with AIDS after diagnosis of Pneumocystis carinii pneumonia in the United States. JID 2001; 183: 1409-12.
21. Bartlett JA, DeMasi R, Quinn J, et al. Overview of the effectiveness of triple combination therapy in antiretroviral-naïve HIV-1 infected adults. AIDS 2001; 15: 1369-77.
22. Murphy RL, Brun S, Hicks C**, et al**. ABT-378/ritonavir plus stavudine and lamivudine for the treatment of antiretroviral-naïve adults with HIV-1 infection: 48 week results. AIDS 2001; 15:1-9.
23. Cockcroft DW, Gault MH. Prediction of creatinine clearance from serum creatinine. Nephron 1976; 16: 31-41.
24. Marcus K, Truffa M, Boxwell D, Toerner J. Recently identified adverse events secondary to NRTI therapy in HIV-infected individuals: Cases from the FDA’s adverse event reporting system (AERS), 9^th^ Conference on Retroviruses and Opportunistic Infections, 2002, Late Breaker Abstract LB14.
25. Agresti A., Categorical Data Analysis. John Wiley and Sons, New York, 1990.

#### APPENDIX I: A5164 CRITERIA FOR AIDS-RELATED OPPORTUNISTIC INFECTIONS OR BACTERIAL INFECTIONS

IMPORTANT NOTE: The definitions below are only to be used for study eligibility, and are not to be used to define events after study entry. The ACTG Diagnosis Appendix 50 will be used to define events that occur on study.

(Confirmed and probable except as noted)

- *Pneumocystis carinii* pneumonia (PCP)
- Bacterial pneumonia (subjects must also have CD4^+^ count < 200 cells/mm^3^)
- Cryptococcal meningitis (confirmed only)
- Disseminated histoplasmosis
- Disseminated *Mycobacterium avium* complex
- Cytomegalovirus retinitis
- Cytomegalovirus encephalitis
- Toxoplasmic encephalitis
- Other atypical mycobacterial infections (non-tuberculous, non-MAC)
- Other serious, invasive bacterial infections (bacterial infection of deep tissue, body cavity, or other normally sterile site) (accompanied by CD4+ cell count < 200 cells/mm^3^)
- **Other serious OIs, including other AIDS-defining and AIDS-related OIs, for which appropriate therapy other than ART exists, with permission of the protocol chair or vice chair.**

**NOTE: TB alone is not a qualifying OI.**

A. *Pneumocystis carinii* pneumonia (confirmed)

1. A history (within the past 3 months) of shortness of breath, dyspnea on exertion, cough, or fever.

AND

2. Histological or cytological evidence of *P. carinii* on bronchoalveolar lavage, lung biopsy, or sputum specimen.

B. *Pneumocystis carinii* pneumonia (PCP) (probable)

1. A history (within the past 3 months) of shortness of breath, dyspnea on exertion, cough, or fever.

AND

2. Abnormal chest x-ray (or CT scan) or hypoxemic arterial blood gas PaO_2_ value less than 80 mm Hg or (A-a) DO_2_ greater than 15 mm Hg, on room air.

AND

3. Specific anti-pneumocystis therapy was initiated.

1. Bacterial pneumonia (confirmed) accompanied by CD4+ cell count < 200 cells/mm^3^

1. Chest radiographic examination shows new or progressive infiltrate, consolidation, or cavitation.

AND

2. At least one of the following:

- 1. Bacterial organism(s) cultured from blood with no alternative site of infection.
  2. Isolation of a bacterial pathogen(s) from a culture specimen obtained by transtracheal aspirate, protected bronchial brushing, or biopsy
  3. Histopathologic evidence of pneumonia with bacterial organism(s) demonstrated by Gram stain or culture of tissue specimen or positive Quellung test for pneumococcus.
  4. Demonstration of a predominant bacterial organism by positive culture or Gram stain of an adequate sputum specimen (fewer than 10 epithelial cells and greater than 25 PMNs per high-power field).
  5. Fluorescent antibody or other antigen detection method positive for *Legionella*, *Chlamydia*, or *Mycoplasma* spp. And no other pathogen identified.

1. Bacterial pneumonia (probable) accompanied by CD4+ cell count < 200 cells/mm^3^

1. Chest radiographic examination shows new or progressive infiltrate, consolidation, or cavitation.

AND

2. At least one of the following:

a. Fever and/or cough.

b. New onset of purulent sputum or change in character of sputum

c. Appropriately collected (acute and convalescent) serologic tests positive for *Legionella*, *Chlamydia*, or *Mycoplasma* and no other pathogen identified.

AND

3. Appropriate antibacterial therapy initiated.

E. Cryptococcal meningitis (confirmed only)

1. Identification of cryptococcus in CSF or CNS tissue by:

a. Positive culture

OR

b. Histopathology of cryptococcal organisms

OR

c. Positive CSF cryptococcal antigen and/or CSF India Ink preparation

F. Disseminated histoplasmosis (confirmed)

1. Identification of the fungal organism H. capsulatum by:

a. Positive culture

OR

b. Positive histopathology: Identification of characteristic appearance of organism within body tissue or fluids.

OR

c. Detection of positive histoplasma antigen > 1 unit obtained from body fluid.

NOTE: This definition of confirmed diagnosis is for A5164 only and applies if subjects do not have a prior diagnosis or treatment for disseminated histoplasmosis.

G. Disseminated histoplasmosis (probable)

1. Compatible clinical syndrome consisting of one or more signs or symptoms as follows: anemia, leukopenia, thrombocytopenia, elevated alkaline phosphatase, ALT, LDH, or bilirubin, enlarged lymph nodes, spleen and/or liver, skin lesions, or gastrointestinal ulcers.

AND

2. Specific antifungal therapy initiated.

H. Disseminated *Mycobacterium avium* complex (confirmed)

1. MAC cultured from blood, bone marrow, lymph node, liver, cerebrospinal fluid or other normally sterile body fluid, tissue, or organ.

I. Disseminated *Mycobacterium avium* complex (probable)

1. A clinical MAC syndrome consisting of one or more of the following: persistent fever ≥ 38 ºC for more than one week, night sweats, diarrhea, weight loss or wasting, hepatomegaly, splenomegaly, anemia (hemoglobin < 8.5 gm/dL), and alkaline phosphatase elevated to greater than twice the upper limit of normal.

AND

2. Treatment initiated for MAC.

J. Cytomegalovirus retinitis (confirmed)

1. Typical lesions including white areas with or without hemorrhages and/or gray-white areas of retinal necrosis with or without hemorrhages. Lesion(s) has/have irregular, dry-appearing granular border, with little or no overlying vitreous inflammation. Must be diagnosed by an experienced ophthalmologist using indirect ophthalmoscopy and documented by retinal photography that can be independently verified.

K. Cytomegalovirus retinitis (probable)

1. Typical lesions including white areas with or without hemorrhages and/or gray-white areas of retinal necrosis with or without hemorrhages. Lesion(s) has/have irregular, dry-appearing granular border, with little or no overlying vitreous inflammation. Must be diagnosed by an experienced ophthalmologist using indirect ophthalmoscopy, but is not documented by retinal photographs.

L. Cytomegalovirus encephalitis (confirmed)

1. Rapidly progressive cognitive impairment, progressive change in mental status or delirium, or signs and symptoms of brain stem injury.

AND

2. Detection of viral nucleic acids (e.g., PCR) in CSF or CSF CMV culture positive or brain biopsy demonstrating CMV by antigen, detection of viral nucleic acids (e.g., PCR) or characteristic cytopathic changes.

M. Cytomegalovirus encephalitis (probable)

1. Rapidly progressive cognitive impairment, progressive change in mental status or delirium, or signs and symptoms of brain stem injury.

AND

2. MRI or contrast CT scan performed which:

a. Excludes toxoplasmosis, lymphoma, PML, or other intracranial process.

AND

b. Demonstrates periventricular inflammation or meningeal enhancement.

AND

3. Other etiologies ruled out.

AND

4. CMV end-organ disease (e.g., retinitis, colitis) present.

AND

5. Specific therapy initiated.

N. Toxoplasmic Encephalitis (confirmed)

Histologic evidence of *Toxoplasma gondii* in tissue or fluid obtained by brain biopsy or aspirate.

OR

All of the following:

a. Compatible clinical syndrome consisting of headache, seizure, neurologic deficits, and/or fever.

b. Presence of characteristic mass lesion(s) on brain imaging study (CT or MRI.)

1. Positive PCR testing of CSF for toxoplasma

**To address safety concerns, subjects should not be entered if they have had toxoplasmic lesions at critical locations or with significant mass effect or intra-cranial hypertension that could lead to worsened neurological outcome or cerebral herniation if exacerbated by increased inflammation in response to early HAART introduction (IRIS).**

O. Toxoplasmic Encephalitis (probable)

All of the following:

a. Compatible clinical syndrome consisting of headache, seizure, neurologic deficits, and/or fever.

b. Presence of characteristic mass lesion(s) on brain imaging study (CT or MRI.)

c. Positive serology for toxoplasmosis

To address safety concerns, subjects should not be entered if they have had toxoplasmic lesions at critical locations or with significant mass effect or intra-cranial hypertension that could lead to worsened neurological outcome or cerebral herniation if exacerbated by increased inflammation in response to early HAART introduction (IRIS).

1. Mycobacterial infection, other non-tuberculous, non-MAC (confirmed)

Other mycobacterial species cultured from blood, bone marrow, lymph node, liver, cerebrospinal fluid, or any other normally sterile body fluid, tissue, or organ.

1. Mycobacterial infection, other non-tuberculous, non-MAC (probable)
2. Other mycobacterial species cultured from bronchopulmonary, gastrointestinal, urine, skin surface, or other non-sterile site(s)

AND

1. Clinical symptoms, signs, or radiograph/laboratory abnormalities compatible with mycobacterial infection consisting of one or more of the following: persistent fever ≥ 38°C for more than one week, night sweats, diarrhea, weight loss or wasting, radiographically documented pulmonary infiltrates, hepatomegaly, splenomegaly, anemia (hemaglobin < 8.5 gm/dL), and alkaline phosphatase elevated to greater than twice the upper limit of normal.

AND

1. No alternative pathogen(s) identified or symptoms/signs persist after treatment for and/or elimination of alternative pathogen(s).

AND

1. Treatment initiated or recommended for non-tuberculous, non-MAC mycobacteria.
2. Bacterial infection of deep tissue, body cavity, or other normally sterile site AND accompanied by a CD4+ cell count < 200 (confirmed)

This category includes, for example, organ parenchymal, deep soft tissue (including pyomyositis) or abdominal abscesses, empyema, purulent pericarditis, meningitis, and bone and joint infections.

1. Demonstration of bacterial pathogen(s) in deep tissue, viscera, body cavity, or other normally sterile site by one of the following methods:
   1. Isolation of a bacterial pathogen(s) from an aspirate or biopsy specimen.

- 1. Appropriate histopathology stain of a specimen.

- 1. Demonstration of bacterial pathogen(s) by appropriate Gram or microbiological stain or antigen detection from aspirate or biopsy specimen

1. Bacterial infection of deep tissue, body cavity, or other normally sterile site AND accompanied by a CD4+ cell count < 200 (probable)

1. Evidence of an infection in a deep tissue, body cavity, or other normally sterile site demonstrated by appropriate diagnostic sampling or imaging procedures such as fluid aspiration, biopsy, computerized tomography, ultrasonography, magnetic resonance imaging, radioisotope scanning, or plain radiograph.

AND

1. Clinical signs and symptoms compatible with the infection.

AND

1. Appropriate treatment initiated and response demonstrated. (Appropriate treatment may include drainage procedures and/or antibacterial therapy.)

**T. Other serious OIs, including other AIDS-defining and AIDS-related OIs, with approval from protocol chairs**

- 1. **Presence of a serious OI, including other AIDS-defining or AIDS-related OI (using the most current World Health Organization (WHO) or Centers for Disease Control and Prevention (CDC) criteria), that is not included in the list above, for which appropriate therapy other than ART exists. See the A5164 PSWP for more information.**

**NOTE: TB alone is not a qualifying OI.**

**AND**

- 1. **Review and approval by A5164 protocol chai**r **or vice chair**.

#### APPENDIX II: GUIDELINES FOR SUBJECT FOLLOW UP

To enhance subject retention, it is suggested that sites strongly consider contacting subjects after study entry (either by phone or in person) at the time points noted in the protocol. If a subject is in the hospital at the specific contact time point, a visit to the room is suggested.

I. Discussion of Follow up Contact with the Subject

We suggest that at entry site personnel obtain a phone number and/or address for each subject. Additionally, study site personnel should discuss with the subject best times to contact the subject by telephone or in person as well as explore confidentiality issues related to calling the subject (e.g., is it OK to call the subject at their residence, is it acceptable to leave messages for the subject with family, roommates, etc., if the subject is not in). Confidentiality of subjects’ information (phone number, diagnosis) should be strongly emphasized. Site personnel should also communicate to subjects that the phone calls or in-person contacts are not mandatory for study participation, but are done to provide additional support for subjects.

For subjects without phones (or even those with phones), partnership with community-based organizations (CBOs) or other agencies that can provide home visits, when FDA approved, should be strongly considered.

II. Content of Contact: Reviewing Medical Treatment

For Arm A, calls or visits should take place 2-4 days after study entry. A second contact should take place 14-21 days after study entry. For Arm A, the follow up contacts should take place at weeks 20, 28, 36 and 44. For Arm B subjects, contacts should also be performed 2-4 days and 14-21 days after study entry and after Step 2 entry. Also for Arm B, the follow up contacts should take place at weeks +20, +28, and +36.

The following issues are suggested to be covered:

1. Adverse drug effects and subject's tolerance of ART (Arm A only)
and other non-antiretroviral medications (e.g., antibiotics) (Arms A and B)

2. Review how subject is dosing medications (Arm A only)

1. Is subject taking the proper amount of pills at dosing times?
2. Is subject dosing medications at proper intervals (e.g., approximately every 12 hours for bid meds)?
3. Are food restrictions (if any) understood?

III. Additional Phone Call Topics: Case Management

Additionally, study site personnel may identify subjects with specific needs. We suggest that the following topics should be considered for discussion with subjects, especially if in the course of discussion of items in section II, specific needs are expressed or identified:

1. receipt (or lack thereof) of follow up appointments with physicians, nurses, etc.

2. receipt (or lack thereof) of medications

3. housing

4. substance abuse (referrals)

5. mental health (referrals)

6. food (e.g. vouchers)/dietician

7. benefits (including ADAP)

8. transportation

Subjects who have difficulties with the above should be referred by study site personnel to the appropriate agencies within the local site, if available. Additionally, study site personnel may wish to contact the subject’s provider to communicate the needs of the subject so that appropriate resources can be assembled and referrals made. Serious adverse drug effects or serious problems with adherence should be communicated to the subject's provider

For subjects without phone (or even those with phones), partnership with community-based organizations (CBOs) or other agencies that can provide home visits, if available, should strongly be considered.

IV. Subject follow up will be documented in the source document only, not in the CRFs.

#### APPENDIX III: PROCEDURES AND INSTRUCTIONS FOR VIROLOGIC AND IMMUNOLOGIC SPECIMENS

I. Overview

This appendix outlines the procedures for **on-study** real-time plasma HIV-1 RNA testing, scheduled storing of PBMC and plasma, and real-time genotyping at the time of virologic failures in A5164.

The material in the following sections applies only to ACTG sites located in the United States. Sites outside the United States will follow site-specific instruction supplied to them by the study team.

II. Sample processing

- 1. Plasma HIV-1 RNA testing will be done **as indicated in protocol section 6.1, Schedule of Events** (plus any confirmatory testing. This will involve drawing one purple top EDTA tube (minimum of 10 mL), separating plasma from PBMC, **cryopreserving** plasma, and**, for on-study samples,** sending it to Johns Hopkins Laboratory. Please follow the standard procedure for handling and shipping to the Johns Hopkins Laboratory as described on the ACTG webpage.
  2. The processing time for plasma HIV-1 RNA has been changed on the LPC from within 6-8 hours to within 30 hours to be more consistent with other ACTG protocols, and to prevent undue hardship at the collection sites. Although current ACTG guidelines recommend that processing still occur as soon as possible, the guidelines allow for up to 48 hours for processing. It is the recommendation of the A5164 team that processing should occur as soon as possible (e.g. within 8 hours) following collection, but that processing must occur within 30 hours of collection.
  3. Plasma and PBMCs should be stored **as indicated in protocol section 6.1, Schedule of Events**. Plasma and PBMCs should also be stored at the time of a suspected IRIS. This will involve drawing four 10-mL purple top tubes and following the instructions outlined in the sections IV-VI for processing and storing plasma and PBMCs.

III. Real-time genotyping at the time of virologic failure

Rationale and plan:

In subjects with virologic failure defined according to section 6.3.12, real-time HIV-1 genotyping will be performed at the Stanford University VSL. A genotype will be obtained on the plasma sample obtained at the time of confirmed virologic failure and on the baseline (entry) plasma sample from the same subject. This will be done to help physicians and study subjects understand the basis of their virologic failure by helping to differentiate between (a) drug resistance prior to study entry, (b) drug resistance developing on study, and (c) the absence of drug resistance. It is expected to also help subjects choose salvage therapy, if that becomes necessary. Having baseline and follow-up samples performed at the same time will also improve quality control because many aspects of the sequence should be unique to a subject despite the intervening treatment. **Please note that sites may choose not to have a real-time genotype performed at the time of virologic failure; a sample for genotyping must still be collected, however, and stored as directed in the A5164 Laboratory Processing Chart.**

Specimen collection and processing:

Subjects with an unconfirmed plasma HIV-1 RNA level indicative of virologic failure should have a repeat sample within 2-4 weeks. This repeat sample should be sent to Johns Hopkins laboratory for a confirmatory RNA level. If the repeat plasma HIV-1 RNA level is found to confirm the virologic failure, then a separate sample from that time point should be **collected for shipment** to the Stanford laboratory. The plasma HIV-1 RNA level of the sample should be noted on the case report form that accompanies the sample. When the Stanford laboratory receives the sample, the laboratory will sequence the confirmed failure sample as well as the baseline sample from the same person. Because of the requirement for a baseline sample in subjects experiencing a virologic failure, it is essential that sites maintain three cryopreserved 1-mL aliquots from study entry at their site (rather than forwarding all of their plasma from this time point to the central repository).

Sites collecting samples for resistance testing should collect blood in purple-top EDTA tubes, should separate plasma using standard ACTG virology procedures, and should cryopreserve five 1-mL aliquots. Three of these 1-mL aliquots should be shipped on dry ice to the Stanford VSL at the address shown below. Samples should be shipped on Mondays, Tuesdays, and Wednesdays.

Genotyping method:

The Stanford University VSL method uses standard commercially available reagents to extract RNA from plasma, to reverse transcribe extracted RNA to cDNA, and to PCR amplify (nested) cDNA to a quantity sufficient or dideoxynucleotide cycle sequencing. Dye-labeled terminators purchased from Applied Biosystem, Inc. (ABI) and the products of the sequencing reaction are separated on an ABI 377 sequencer. Standard physical precautions **should be taken** to prevent laboratory contamination as well as phylogenetic analyses to detect contamination and sample mix-ups.

The expected turnaround for real-time sequencing is 7-10 days. If a sequence needs to be repeated, the subject's physician will be contacted before the 10th day and informed that the results of sequencing may be delayed.

The Stanford University VSL is currently applying for CLIA certification.

Genotypic interpretation and reporting:

Because there is no standard for genotypic interpretation within the ACTG, each genotype will be accompanied by a rules-based interpretation used by the Stanford Diagnostic Virology laboratory. This interpretation is also available on line on the Stanford HIV RT and Protease Sequence Database (http://hivdb.stanford.edu). A detailed description of the interpretation system is described in the program's release notes. The genotype report will be faxed to the site designated to receive virology results. The genotypic data will be submitted to the ACTG Data Management Center.

Physicians with questions about the genotypic report can email or phone the protocol virologist who will be expected to respond within 2-3 working days. If the protocol virologist is not available, the protocol chair will answer questions pertaining to the genotype.

Protocol virologist / contact for question about genotype reports

Robert W. Shafer, M.D.

Tel: 650-725-2946

Email: rshafer@stanford.edu

Shipping address of **Stanford University** VSL:

Mark Winters, M.S.

Stanford University Medical Center

Division of Infectious Diseases

Room S-146

**300 Pasteur Drive**

Stanford, CA, 94305**-5107**

Phone: (650) 723-8291

Fax: (650) 725-2395

E-Mail: [mark.winters@stanford.edu](mailto:mark.winters@stanford.edu)

IV. Procedures for Processing Purple-Top EDTA Tubes

The purple-top EDTA tube should be at room temperature (18°-25°C) prior to use.

After collection of blood (fill the tube entirely), the tube should be held upright at room temperature until centrifugation. Blood samples should be centrifuged and plasma frozen within 30 hours of blood collection for best results.

SAMPLES SHOULD BE TRANSPORTED TO THE LABORATORY IMMEDIATELY.

The centrifugation step is critical for optimal cell yield. Centrifuge tube/blood sample at room temperature (18°-25°C) for 10 minutes at 400 x *g*.

After centrifugation, remove the separated plasma and transfer to a clean, conical centrifuge tube. Re-centrifuge the separated plasma at 800 x *g* for an additional 10 minutes to remove cell debris.

V. Procedures for Processing and Storing Plasma

Aliquots of plasma will be stored after the plasma is cleared of platelets and cell debris by a second centrifugation.

For a 10 mL draw capacity, expect a yield of roughly 10 mL of plasma when 2 tubes are drawn. Prepare 10 aliquots containing at least 1.0 mL of plasma (pooled from the 2 EDTA tubes) and freeze at −70°C.

VI. Procedure for Banking PBMCs at Clinical Site

Banking of PBMCs (on specimens from entry, week 12, week 24, and week 48 (or close-out))

After plasma removal, the remaining anticoagulated blood will be diluted and PBMCs separated by Ficoll-hypaque centrifugation performed according to the latest version of the ACTG Virology Manual. After the PBMCs are washed and precisely counted, cryopreserved according to the Immunology Consensus Method and nonviable PBMCs will be banked according to the most recent ACTG Virology Manual:

a) TWO aliquots of >5 x 10^6^ cells each in 1.0 mL DMSO freezing medium containing 10% sterile DMSO and 90% heat-inactivated fetal calf serum. This medium should be prepared freshly for each freezing procedure and cooled to 2°-8°C prior to use. Cool cryovials in the bottom of a **polystyrene** container in the bottom of a −70°C freezer for 2-24 hours prior to storing on vapor-phase liquid nitrogen (viably cryopreserved cells).

b) TWO aliquots of >2 x 10^6^ cells each spun in a sterile plastic microcentrifuge tube and dry cell pellets stored at −70°C (nonviable PBMCs).

c) All remaining PBMCs should be stored as viable cryopreserved cells of 5 x 10^6^ cells/aliquot in 1.0 mL of DMSO freezing medium containing 90% fetal calf serum and stored on liquid nitrogen.

Materials:

1. Anticoagulated blood (purple-top EDTA tubes)

2. Fetal calf serum (heat inactivated)

3. DMSO (ACS spectrophotometric grade [Aldrich, Milwaukee, WI; catalog #27,043-1]). DMSO must be fresh. Once the bottle is opened, the shelf-life of DMSO is <6 months.

4. Cryovials (2 mL, screw cap)

5. Nalgene “Mr. Frosty” cryovials (Curtis Matheson Scientific, Houston, TX; catalog #288-383).

6. Optional: cryotube holder that stabilizes tubes on ice (Corning workstation from Fisher Scientific; catalog #03-374-37A)

7. RPMI 1640 media + 10% fetal calf serum.

Freezing Procedure:

1. Label cryovials and chill at −20°C for 30 minutes.

2. Prepare freezing solution: 90% fetal calf serum + 10% DMSO; chill on ice.

3. Prepare PBMC by Ficoll-hypaque density centrifugation; wash twice at 4°C. Count cells, wash again at 4°C. (After Ficoll separation, keep cells chilled or on ice at all times).

4. Resuspend cells in freezing solution at 1 x 10^7^ cells/mL; aliquot 0.5 to 1.0 mL of the cell suspension per cryovial. Be sure cryovial caps are securely tightened.

5. Immediately place cryovials in “Mr. Frosty” cryovials and transfer to a −70°C freezer. Alternatively, place cryovials in a Cryomed® freezing chamber, lowering the temperature at −1°C per minute to −70°C.

6. Transfer cryovials to liquid nitrogen within 24 hours.

All specimens should be logged into the Laboratory Data Management System (LDMS) by the processing laboratory, and computer labels should be generated that have the LDMS specimen number, patient identification number (PID), protocol number, visit identification number (VID), date and time of draw, and the LDMS specimen code. ALWAYS include the virology tracking form and the shipping manifest from the LDMS in the shipping box.

APPENDIX IV

###### DIVISION OF AIDS

AIDS CLINICAL TRIALS GROUP (ACTG)

SAMPLE INFORMED CONSENT

For protocol:

A5164, FINAL Version **3.0** dated **08/30/05**, A Phase IV Study of Antiretroviral Therapy for HIV-Infected Adults Presenting with Acute Opportunistic Infections: Immediate versus Deferred Antiretroviral Treatment

SHORT TITLE FOR THE STUDY: A5164, FINAL Version **3.0** dated **08/30/05**, Immediate vs. Deferred HIV Treatment in Patients with Acute OIs.

##### INTRODUCTION

You are being asked to take part in this research study because it appears to your doctor that you are infected with HIV (the virus that causes AIDS) and that you have a serious “opportunistic infection” or “OI”, (an infection that you get because the HIV has lowered the strength of your immune system) or a serious bacterial infection (BI) that is related to HIV infection.

This study is sponsored by the National Institutes of Health (NIH). The doctor in charge of this study at this site is: (insert name of Principal Investigator). Before you decide if you want to be a part of this study, we want you to know about the study.

This is a consent form. It gives you information about this study. The study staff will talk with you about this information. You are free to ask questions about this study at any time. If you agree to take part in this study, you will be asked to sign this consent form. You will get a copy to keep.

WHY IS THIS STUDY BEING DONE?

The purpose of this study is to learn whether it is better to start drugs that fight HIV right away, while you are being treated for an OI or BI, or wait until after the OI or BI has been treated. It is not known whether the HIV drugs cause side effects that could worsen the problems for seriously ill people such as those with **an** OI or BI. Another purpose of this study is to compare the safety and effectiveness of the HIV drugs when started during or after an OI or BI.

All drugs used against HIV in this study **must** have been approved by the **U.S.** Food and Drug Administration (FDA) for the treatment of HIV infection. **If you join this study, you will not be able to participate at the same time in any other study if that study requires you to take any experimental (not FDA-approved) drugs.**

WHAT DO I HAVE TO DO IF I AM IN THIS STUDY?

Screening

After you have read and signed this consent form, you will have several evaluations done to make sure that you meet the requirements for joining the study. Unless you have already been tested and found to be HIV-positive, you will have about 1 teaspoon of blood drawn and tested to see if you are infected with HIV. You may be asked to sign a separate consent form required by your state or hospital when you are tested for HIV. You will be told the results of the HIV test as soon as it is available. If the test results show that you are HIV-positive, the hospital staff will give you HIV counseling. You will also have a medical history done and, if you are a woman who is able to become pregnant, you will have an extra teaspoon of blood taken or you will give a urine sample for a pregnancy test. You will get the result of the pregnancy test as soon as it is available. About 3 tablespoons of blood will be taken for routine blood tests, and for a CD4+ cell count (the number of white blood cells that fight infection).

Pre-entry

At least 24 hours before you enter the study, you will have about 1 teaspoon of blood drawn for an HIV viral load test (amount of HIV in your blood). **This sample could have been drawn up to 4 weeks before you enter the study.**

Entry

If the results from your screening evaluations allow you to take part in this study, you will be asked to enter the study. You will have a physical exam and have about 4-5 tablespoons of blood drawn for routine tests, HIV viral load, CD4+ cell count, and to see how much fat is in your blood. Throughout the study**,** you will be told your HIV viral load results and CD4+ cell counts as they become available. Some of your blood drawn at the entry visit will be stored (with usual protectors of identity) for possible future testing including a test to see if you have become resistant to any of the anti-HIV drugs (meaning that these drugs might not work against your HIV).

You will also be asked to complete a questionnaire to find out how you feel about your health. This questionnaire should take about 10 minutes.

You will be randomized (assigned by chance, like flipping a coin) to one of two groups (Arm A or Arm B). You have an equal (50/50) chance of being assigned to either of the two treatment groups. You will be told which group you are to be in.

Arm A

If you are in Arm A, you will receive a combination of anti-HIV drugs within 2 weeks after starting treatment for your OI or BI, and will continue to receive the usual care for your particular infection. You and your doctor will decide what drugs you will take for treatment against HIV.

The drugs **provided** for the treatment of HIV in this study **are:**

lopinavir/ritonavir **[LPV/RTV** (Kaletra™)**]**

**emtricitabine/tenofovir [FTC/TDF (Truvada®)]**

stavudine **[**d4T **(Zerit™**)**]**

**If you and your doctor choose Kaletra plus d4T, you should also take up to** one other drug chosen by your doctor. The other drug will not be supplied by the study. What you will receive will depend on your doctor’s advice. If you can start taking the drugs within the timeframe suggested, **Kaletra,** **Truvada, and/or** d4T will be provided by the study until you complete 48 weeks on the study. **Please note that if you entered A5164 before Truvada was provided and you would now like to switch to or add study-provided Truvada, you may do so, in consultation with your physician.**

OR

Arm B

If you are in Arm B, you will receive the standard medications for your particular OI or BI. Anti-HIV drugs **should be started** at least 4 weeks after you enter the study, but no later than 32 weeks after you enter the study. Depending on how fast you recover from your OI or BI, your doctor and you will decide when it is best to start HIV treatment. You and your doctor will decide what drugs you will take for treatment against HIV.

The drugs **provided** for the treatment of HIV in this study **are:**

lopinavir/ritonavir **[LPV/RTV** (Kaletra**®**)**]**

**emtricitabine/tenofovir [FTC/TDF (Truvada®)]**

stavudine **[**d4T **(Zerit™**)**]**

**If you and your doctor choose Kaletra plus d4T, you should also take up to** one other drug chosen by your doctor. The other drug will not be supplied by the study. What you will receive will depend on your doctor’s advice. If you can start taking the drugs in the time suggested, **Kaletra,** **Truvada, and/or** d4T will be provided by the study until you complete 48 weeks on the study. **You will not receive 48 weeks of anti-HIV treatment. Please note that if you entered A5164 before Truvada was provided and you would now like to switch to or add study-provided Truvada, you may do so, in consultation with your physician.**

For Both Arms A and B:

Besides lopinavir/ritonavir (Kaletra), **emtricitabine/tenofovir (FTC/TDF [Truvada]), and/or** stavudine (d4T)**,** other antiretroviral drug**s** that **are** recommended (but not required and not provided) for use in this study **are** lamivudine (3TC) **or emtricitabine (FTC)**. **3TC or FTC may be taken together with Kaletra and d4T, but should not be taken together with Truvada.**

During the Study:

You will have study visits every 4 weeks for 16 weeks and then every 8 weeks until you have been on the study for 48 weeks (about 1 year). You will have physical exams and routine blood tests to see how well you are recovering. At each of these times, you will have between 1 and 5 tablespoons of blood drawn (depending on the visit) for routine tests, HIV viral load, CD4+ cell count, and measuring the amount of fat in your blood. If the results of the tests for fat in your blood are above a specific level, you will be asked to return to the clinic within 2 weeks “fasting” (without eating or drinking for at least 8 hours before the visit) to be tested again.

You will also be asked at specific visits to complete some questionnaires to find out how well you are taking your drugs and how you feel about your health. These questionnaires should take up to about 20 minutes each time.

If you are a woman who can become pregnant a blood or urine pregnancy test will be done if it is suspected that you are pregnant. You will get the results of the pregnancy test as soon as it is available. If you become pregnant while on the study, and you want to continue on the study, you must sign a special informed consent.

If a HIV viral load test shows that your anti-HIV drugs are not working as well as expected, you will be asked to come back within 2-4 weeks and be retested to make sure the results are correct. You will also have about 1 teaspoon of blood drawn and tested to see if you have become resistant to any of the anti-HIV drugs.

At different times during the study, the people from the clinic may call you on the telephone to see how you are doing and to remind you of your upcoming clinic visits.

Some of your blood that is left over after all required study testing is done may be stored (with usual protectors of identity) and used for ACTG-approved HIV-related research in the future.

Premature Treatment Discontinuation or Premature Study Discontinuation

If there is a situation during the study where the doctor needs to stop your anti-HIV treatment, you will be asked to come in for a physical exam. You will have about 5 tablespoons of blood drawn for routine tests, HIV viral load, CD4+ cell count, and measuring the amount of fat in your blood. You will also be asked to complete the questionnaires on how you are taking your drugs, and how you feel about your health. These questionnaires should **take** about 20 minutes **to complete**. You will continue on study and have all the study visits up through week 48.

ARE THERE ANY SUBSTUDIES IN THE STUDY?

There **was** one substudy in the study**, but it is no longer open to enrollment**.

HOW MANY PEOPLE WILL TAKE PART IN THIS STUDY?

About 282 people will take part in this study with 141 people in each arm.

##### HOW LONG WILL I BE IN THIS STUDY?

You will be in this study for 48 weeks.

WHY WOULD THE DOCTOR TAKE ME OFF THIS STUDY EARLY?

The study doctor may need to take you off the study early without your permission if:

- the study is cancelled by the National Institutes of Health (NIH), the drug companies supporting this study, or the site’s Institutional Review Board (IRB). (An IRB is a committee that watches over the safety and rights of research subjects.)
- the Data Safety Monitoring Board (DSMB) recommends that the study be stopped early. (A DSMB is an outside group of experts who monitor the safety of the study.)
- your doctor feels that it is not in your best interest for you to participate.
- you are not able to attend the study visits as required by the study.
- your HIV test does not confirm that you are HIV positive.

If you decide that you do not want to participate in the study anymore, you may leave the study at any time.

The study doctor may also need to take you off the study drug(s) without your permission if:

- continuing the study drug(s) may be harmful to you.
- you need a treatment that you may not take while on the study.
- you are not able to take the study drug(s) as required by the study.
- you begin to breastfeed your baby.

If you must stop taking the study drug(s) before the study is over, the study doctor may ask you to continue to be part of the study and return for some study visits and procedures.

**WHAT IF I HAVE TO STOP TAKING THE STUDY DRUGS?**

**During the study:**

**If you must permanently stop taking study-provided drugs (LPV/RTV, FTC/TDF, and/or d4T) before your study participation is over, the study staff will discuss other options that may be of benefit to you.**

**After the study:**

**After you have completed your study participation, the study will not be able to continue to provide you with study-provided drugs (LPV/RTV, FTC/TDF, and/or d4T). If continuing to take these or similar drugs would be of benefit to you, the study staff will discuss how you may be able to obtain them.**

WHAT ARE THE RISKS OF THE STUDY?

Risks of Blood Draws:

- Discomfort
- Bruising
- Bleeding
- Swelling in the area where the needle enters the body
- Small risk of infection

Risk of Immune Reconstitution Inflammatory Syndrome

While being treated with anti-HIV drugs, your improving immune system's strong response to opportunistic infections may also cause illness (**which** is called immune reconstitution inflammatory syndrome**, or IRIS**). Usually, it causes a return or worsening of at least some of the symptoms of your OI.

Some examples of what could happen are that your lymph nodes (small organs in your body that help filter disease germs from the blood) could swell up, you could get a high fever, and if you are being treated for a lung infection, you might have worsened cough and shortness of breath. While some of these reactions can be serious, they usually last for a short time and can be treated without stopping the anti-HIV drugs. If this reaction happens to you, you will be treated for the problem and asked to have some additional blood drawn for testing of your immune system at that time.

Possible risks of starting HIV drugs during your current infection

The risks of taking anti-HIV drugs during treatment for an **OI** are not known. The risks of earlier treatment may include added side effects from the medications, either from the drug used against HIV or the drugs taken against the **OI**. More severe interactions between drugs may also cause trouble. Or there may be an increase in the chance of getting immune reconstitution inflammatory syndrome.

Drug Risks

There may be a risk of serious and/or life-threatening side effects when non-study medications are taken with the study drugs. For your safety, you must tell the study doctor or nurse about all medications you are taking before you start the study and also before starting any new medications while on the study. Also, you must tell the study doctor or nurse before enrolling in any other clinical trials while on this study.

The drugs used in this study may have side effects, some of which are listed below. Please note that these lists do not include all the side effects seen with these drugs. These lists include the more serious or common side effects with a known or possible relationship. If you have questions concerning the additional study drug side effects, please ask the medical staff at your site.

Protease Inhibitor

The use of potent antiretroviral drug combinations (which commonly include a protease inhibitor) may be associated with an abnormal placement of body fat and wasting (loss of weight and body fat). Some of the body changes include:

- Increase in fat around the waist and stomach area
- Increase in fat on the back of the neck
- Thinning of the face, legs, and arms
- Breast enlargement

The use of potent antiretroviral drug combinations (which commonly include a protease inhibitor) may also be associated with altered fat metabolism including elevated triglycerides and/or elevated cholesterol.

The use of protease inhibitors may be associated with the development of or the worsening of elevations in blood sugar and diabetes.

There have been reports of increased bleeding in HIV-infected persons with hemophilia who were treated with protease inhibitors. It is not known if protease inhibitors were the cause of these bleeding episodes.

Lopinavir/Ritonavir (KALETRA®)

The following side effects are also associated with the use of lopinavir/ritonavir:

- Gastrointestinal disturbances, including abdominal pain, upset stomach, vomiting, abnormal bowel movements (or stools), and loose or watery stools
- Large increases in the amount of fatty acid in the blood
- Large increases in cholesterol in the blood
- Abnormal liver function tests (increased liver enzymes) and worsening liver disease, possibly leading to death
- Elevation of pancreatic enzymes and inflammation of the pancreas (pancreatitis) which may result in death
- Feeling weak/tired
- Headache
- Rash (seen in children)

Nucleoside Analogue**s**

Lactic acidosis and severe hepatomegaly (enlarged liver) with steatosis (fatty liver) that may result in liver failure, other complications**,** and death have been reported with the use of antiretroviral nucleoside analogues alone or in combination. The liver complications and death have been seen more often in women on these drug regimens. Some nonspecific symptoms that might indicate lactic acidosis include: unexplained weight loss, stomach discomfort, nausea, vomiting, fatigue, weakness, and shortness of breath.

Stavudine**, d4T** (ZERIT®)

**The following side effects have been associated with the use of stavudine, d4T:**

- **Deaths from liver failure have been reported in pregnant women receiving the combination of stavudine and didanosine with other anti-HIV drugs.**
- **People who take stavudine together with didanosine, with or without hydroxyurea, may be at greater risk for pancreatitis or liver problems or both. These conditions may result in death.**
- Numbness, tingling, and pain in your hands or feet
- **Pancreatitis (inflammation of the pancreas), which may cause death. If you develop pancreatitis, you may have one or more of the following: stomach pain, nausea, and vomiting.**
- Rash
- Upset stomach, vomiting**,** **and loose or watery stools**
- Abdominal pain
- Abnormal liver function blood tests **or** abnormal pancreatic function blood tests
- Rare cases of muscle weakness, which may progress to paralysis and inability to breathe (This may be associated with elevation of lactic acid in the blood).

When stavudine is used with other medicines with similar side effects, these side effects may be seen more often, and may be more severe, than when stavudine is used alone.

Lamivudine, 3TC (EPIVIR®)

The following side effects have also been associated with use of lamivudine:

- Headache
- Feeling of vague overall discomfort
- Feeling tired
- Dizziness
- Depression
- Upset stomach
- Vomiting
- Loose or watery stools
- Decrease in appetite
- Abdominal cramps
- Sleeplessness
- Rash
- Numbness, tingling, and pain in the hands or feet
- Decrease in the number of white blood cells that help fight infection
- An increase in a substance in the blood (a type of a pancreatic enzyme) which could mean a problem with the pancreas
- Increased liver function tests, which could mean liver damage

The following side effect has been seen in children: Inflammation of the pancreas with abdominal pain.

People who are infected with both hepatitis B and HIV should be warned that their liver function tests may increase, and symptoms associated with hepatitis (an acute inflammation of the liver) may worsen after lamivudine has been stopped. Although most of these cases have resolved without treatment, some deaths have been reported.

**Emtricitabine, FTC (Emtriva™)**

**The following side effects have been associated with the use of emtricitabine, a nucleoside analogue:**

- - **Headache**
  - **Dizziness**
  - **Tiredness**
  - **Inability to sleep, unusual dreams**
  - **Loose or watery stools**
  - **Upset stomach (nausea) or vomiting**
  - **Abdominal pain**
  - **Rash, itching, which sometimes can be a sign of an allergic reaction**
  - **Skin darkening of the palms and/or soles**
  - **Increased cough**
  - **Runny nose**
  - **Abnormal liver function tests, which could mean liver damage**
  - **Increases in pancreatic enzyme (substances in the blood), which could mean a problem with the pancreas**
  - **Increased triglycerides**
  - **Increased creatine kinase (CK), which could mean muscle damage**

**People who are infected with both hepatitis B and HIV should be warned that their liver function tests may increase, and symptoms associated with hepatitis (an acute inflammation of the liver) may worsen if emtricitabine is stopped.**

**Emtricitabine, FTC/Tenofovir Disoproxil Fumarate, TDF (TRUVADA®)**

**No new or unexpected side effects are observed with the FTC 200 mg/TDF 300 mg combination tablet than those observed when each drug is given separately.**

**Nucleotide Analogue**

**Lactic acidosis and severe hepatomegaly (enlarged liver) with steatosis (fatty liver) that may result in liver failure, other complications, and death have been reported with the use of tenofovir, as well as antiretroviral nucleoside analogues alone or in combination. The liver complications and death have been seen more often in women on these drug regimens. Some nonspecific symptoms that might indicate lactic acidosis include: unexplained weight loss, stomach discomfort, nausea, vomiting, fatigue, weakness, and shortness of breath.**

**Tenofovir Disoproxil Fumarate (Tenofovir DF, TDF, VIREAD®)**

**The following side effects have been associated with the use of tenofovir:**

- **Upset stomach, vomiting, gas, loose or watery stools**
- **Dizziness**
- **Abdominal pain**
- **Lack of energy**
- **Kidney damage or failure**
- **Inflammation or swelling and possible damage to the pancreas**
- **Shortness of breath**
- **Rash**
- **Low phosphate, a chemical in the blood**
- **Higher than normal liver functions tests in children**
- **Allergic reaction, which may include fever, rash, upset stomach, vomiting, loose or watery stools, abdominal pain, achiness, shortness of breath or a general feeling of illness**
- **Changes in bone growth and strength were seen in study animals given tenofovir. It is unknown if taking tenofovir for a long time will cause bone abnormalities in adults. In children, some decrease in bone thickness (density) has been seen.**

**People who are infected with both hepatitis B and HIV should be warned that their liver function tests may increase, and symptoms associated with hepatitis (an acute inflammation of the liver) may worsen if tenofovir is stopped.**

**NOTE: Because there is only a small amount of information on tenofovir in pregnant women, tenofovir should be used during pregnancy only if clearly needed.**

ARE THERE RISKS RELATED TO PREGNANCY?

It is not known if the drug or drug combinations in this study harm unborn babies. Tests in pregnant animals do show some risk. The risks to unborn babies for each drug are listed in the section called "Drug Risks”. If you are having sex that could lead to pregnancy, you must agree not to become pregnant or make a woman pregnant.

Because of the risk involved, you and your partner must use **at least** one method of birth control that you discuss with the study staff. **If you take efavirenz during the study, you will be required to use two methods of birth control.** You must continue to use this method until 4 weeks after stopping **anti-HIV drugs**. You may choose **from** the birth control methods listed below:

- Male or female condoms with or without a cream or gel that kills sperm
- Diaphragm or cervical cap with a cream or gel that kills sperm
- Intrauterine device (IUD)

If you can become pregnant, you must have a pregnancy test before you enter this study. The test must be negative. If you think you may be pregnant at any time during the study, tell your study staff right away. The study staff will talk with you about your choices.

##### Breast-feeding

###### It is unknown whether the study drug combinations pass through the breast milk and may cause harm to your infant. Mothers who breast-feed cannot continue to receive drugs from the study.

ARE THERE BENEFITS TO TAKING PART IN THIS STUDY?

If you take part in this study, there may be a direct benefit to you, but no guarantee can be made. It is also possible that you may receive no benefit from being in this study. Information learned from this study may help others who have HIV.

The potential benefits to receiving early anti-HIV treatment includes the possibility of a better or faster recovery from the OI or BI you are being treated for, and earlier improvement in your immune system that could prevent you from getting the OI or BI again or getting new infections. There is also the possibility of making your anti-HIV drugs control your HIV infection better.

WHAT OTHER CHOICES DO I HAVE BESIDES THIS STUDY?

Instead of being in this study you have the choice of:

- treatment with prescription drugs available to you
- treatment with experimental drugs, if you qualify
- no treatment

Please talk to your doctor about these and other choices available to you. Your doctor will explain the risks and benefits of these choices.

WHAT ABOUT CONFIDENTIALITY?

We will do everything we can to protect your privacy. In addition to the efforts of the study staff to help keep your personal information private, we have obtained a Certificate of Confidentiality from the U.S. Federal Government. This certificate means that researchers cannot be forced to tell people who are not connected with this study, such as the court system, about your participation. Also, any publication of this study will not use your name or identify you personally.

People who may review your records include: (insert name of site) IRB, National Institutes of Health (NIH), study staff, study monitors, drug companies supporting this study, and their designees. Having a Certificate of Confidentiality does not prevent you from releasing information about yourself and your participation in the study.

Even with the Certificate of Confidentiality, if the study staff learns of possible child abuse and/or neglect or a risk of harm to yourself or others, we will be required to tell the proper authorities.

WHAT ARE THE COSTS TO ME?

Taking part in this study may lead to added costs to you and your insurance company. In some cases it is possible that your insurance company will not pay for these costs because you are taking part in a research study. Some of your anti-HIV drugs **may** not be supplied by the study, and therefore will be your responsibility to obtain. The drugs that will be supplied by the study for your possible use are lopinavir/ritonavir **(Kaletra),** **emtricitabine/tenofovir (Truvada), and** stavudine **(d4T)**.

WHAT HAPPENS IF I AM INJURED?

If you are injured as a result of being in this study, you will be given immediate treatment for your injuries. The cost for this treatment will be charged to you or your insurance company. There is no program for compensation either through this institution or the National Institutes of Health (NIH). You will not be giving up any of your legal rights by signing this consent form.

WHAT ARE MY RIGHTS AS A RESEARCH SUBJECT?

Taking part in this study is completely voluntary. You may choose not to take part in this study or leave this study at any time. You will be treated the same no matter what you decide.

We will tell you about new information from this or other studies that may affect your health, welfare, or willingness to stay in this study. If you want the results of the study, let the study staff know.

WHAT DO I DO IF I HAVE QUESTIONS OR PROBLEMS?

For questions about this study or a research-related injury, contact:

- name of the investigator or other study staff
- telephone number of above

For questions about your rights as a research subject, contact:

- name or title of person on the Institutional Review Board (IRB) or other organization appropriate for the site
- telephone number of above

SIGNATURE PAGE – A5164

If you have read this consent form (or had it explained to you), all your questions have been answered and you agree to take part in this study, please sign your name below.

Participant’s Name (print) Participant’s Signature and Date

Participant’s Legal Guardian (print) Legal Guardian’s Signature and Date

(As appropriate)

Study Staff Conducting Study Staff Signature and Date

Consent Discussion (print)

Witness’ Name (print) Witness’ Signature and Date

(As appropriate)

APPENDIX V

DIVISION OF AIDS SAMPLE INFORMED CONSENT

For

WOMEN WHO BECOME PREGNANT WHILE ON STUDY

For protocol:

A5164, FINAL Version **3.0** dated **08/30/05**, A Phase IV Study of Antiretroviral Therapy for HIV-Infected Adults Presenting with Acute Opportunistic Infections: Immediate versus Deferred Antiretroviral Treatment

SHORT TITLE FOR THE STUDY: A5164, FINAL Version **3.0** dated **08/30/05**, Immediate vs. Deferred HIV Treatment in Patients with Acute OIs

INTRODUCTION

Because you are now pregnant, you are being asked if you want to continue taking part in this research study. This study was designed so that women who were pregnant could not join the study. However, because you were already in the study when you became pregnant, you will be allowed to stay in the study whether or not you continue study drugs during your pregnancy.

This is a consent form. It gives you more information about this study and how it may affect your pregnancy and your baby. The study staff will talk with you about this information. You may also talk with your own doctor about what is best for you and your baby, if you should remain on study drugs or choose other anti-HIV drugs. If you agree to stay in this study, you will be asked to sign this consent form. You will get a copy to keep. You are free to ask questions of the study staff at any time.

WHAT DO I HAVE TO DO IF I STAY IN THIS STUDY?

If you decide to carry the baby to birth, you may have to go off some or all of the study drugs, but you will remain on study and continue to have the regular visits and all tests in the main study (A5164).

This study will not provide care related to your pregnancy, the delivery of your baby, or the care of your baby. You must arrange for your care and your baby’s care outside of this study.

Long-term follow-up is recommended for a baby whose mother takes anti-HIV drugs during pregnancy. The study staff will talk with you about long-term follow-up and the possibility of enrolling your baby in a long-term study.

WHAT ARE THE RISKS RELATED TO STAYING IN THE STUDY?

Now that you are pregnant, there are some possible risks you should know. These possible risks to you and your baby are in addition to the risks that are described in the study consent you already signed.

If you are taking efavirenz as one of your anti-HIV drugs on this study, you must stop taking it while you are pregnant. You may start the drug again, once your pregnancy is over.

Risks to You if Staying on Study Drug(s):

1. Different side effects or more severe side effects may occur in pregnant women taking drugs. This may make it more difficult for you to take your study drug(s). Not taking anti-HIV study drug(s) as directed may cause the drug(s) not to work on the HIV in your blood.
2. The amount of drug in the blood may change during pregnancy. This means that possibly your level of anti-HIV drugs may decrease and not work as well or cause the HIV to become resistant to drugs.
3. It is not known if risks of pregnancy might be made worse by study drug(s) and may result in death.

Risks to Your Baby if Staying on Study Drug(s):

1. It is not known if some study drug(s) may cause you to have a baby that is born early or dead.
2. It is not known if some study drug(s) may cause your baby to be sick or have birth defects. Not all birth defects are seen at birth. Some birth defects are seen later as the baby grows.
3. In the U.S., only Zidovudine (ZDV, Retrovir) is approved by the Food and Drug Administration (FDA) to decrease the risk of passing HIV from mother to baby. The U.S. Public Health Service recommends that women discuss with their doctor the use of ZDV alone and with other anti-HIV drugs to decrease the risk of passing HIV to their babies.
4. Please look at the main study’s informed consent (A5164) that you read and signed relating to the specific risks of the drugs you are taking.

BREAST-FEEDING

If you decide to breast-feed your baby you may not continue to take study drugs, but will be asked to continue study visits. Researchers know that HIV can pass through breast-milk and taking study drug(s) has not been proven to decrease the chance of passing HIV through your breast milk to your baby.

ARE THERE BENEFITS TO STAYING IN THIS STUDY?

If you continue to take part in this study, there may be a benefit to you and your baby, but no guarantee can be made. It is also possible that you and your baby will receive no benefit from continuing in this study. Information learned from this study may help others who have HIV.

WHAT OTHER CHOICES DO I HAVE BESIDES STAYING ON STUDY DRUGS?

Instead of staying on the study drugs you have the choice of:

- treatment with prescription drugs available to you
- treatment with experimental drugs being studied for use during pregnancy, if you qualify
- no treatment

Please talk to your doctor about these and other choices available to you. Your doctor will explain the risks and benefits of these choices.

WHAT ABOUT CONFIDENTIALITY?

We will do everything we can to protect your privacy. In addition to the efforts of the study staff to help keep your personal information private, we have gotten a Certificate of Confidentiality from the U.S. Federal Government. This certificate means that researchers cannot be forced to tell people who are not connected with this study, such as the court system, about your participation. Also, any publication of this study will not use your name or identify you personally.

People who may review your records include: (insert name of site) IRB, National Institutes of Health (NIH), study staff, study monitors, drug companies supporting this study, and their designees. Having a Certificate of Confidentiality does not prevent you from releasing information about yourself and your participation in the study.

Even with the Certificate of Confidentiality, if the study staff learns of possible child abuse and/or neglect or a risk of harm to yourself or others, we will be required to tell the proper authorities.

WHAT ARE THE COSTS TO ME?

In addition to any costs that are described in the study consent you already signed, this study will not cover any cost related to your pregnancy, delivery of your baby, or care of your baby.

WHAT HAPPENS IF MY BABY OR I ARE INJURED?

If your baby or you are injured as a result of being in this study, you will both be given immediate treatment for your injuries. The cost for this treatment will be charged to you or your insurance company. There is no program for compensation either through this institution or the National Institutes of Health (NIH). You will not be giving up any of your legal rights by signing this consent form.

WHAT ARE MY RIGHTS AS A RESEARCH SUBJECT?

Continuing to take part in this study is completely voluntary. You may choose not to continue in this study or leave this study at any time. You will be treated the same no matter what you decide.

We will tell you about new information from this or other studies that may affect your health, welfare, or willingness to stay in this study. If you want the results of the study, let the study staff know.

WHAT DO I DO IF I HAVE QUESTIONS OR PROBLEMS?

For questions about this study or a research-related injury, contact:

- site insert name of the investigator or other study staff
- site insert telephone number of above

For questions about your rights as a research subject, contact:

- site insert name or title of person on the Institutional Review Board (IRB) or other organization appropriate for the site
- site insert telephone number of above

SIGNATURE PAGE (Pregnancy consent – A5164)

If you have read this consent form (or had it explained to you), all your questions have been answered and you agree to take part in this study, please sign your name below.

Participant’s Name (print) Participant’s Signature and Date

Participant’s Legal Guardian (print) Legal Guardian’s Signature and Date

(As appropriate)

Study Staff Conducting Study Staff Signature and Date

Consent Discussion (print)

Witness’ Name (print) Witness’ Signature and Date

(As appropriate)
